# Supplementary material for: TSCCA: A tensor sparse CCA method for detecting microRNA-gene patterns from multiple cancers
Source: PLoS Comput Biol. 2021 Jun 1;17(6):e1009044. doi: 10.1371/journal.pcbi.1009044 (PMC8195367; doi:10.1371/journal.pcbi.1009044)
Supplement: S1 Text — (DOCX) [file pcbi.1009044.s001.docx]

**Supporting Information for**

TSCCA: A tensor sparse CCA method for detecting microRNA-gene patterns from multiple cancers

**Contents**

1 Proof of the Proposition 1…………………………………………………………2

2 Stopping condition of TSCCA Algorithm…………………………………………2

3 Convergence Analysis………………………………………….…………………2

4 Computational complexity……..……………………………….…………………3

5 Parameter selection……..………..…………………………….…………………4

6 TSCCA extracts multiple sparse factors/modules………………………………4

7 Overlap analysis of any two modules using permutation test…………………5

8 Statistical analysis of modularity scores using permutation test……..….……5

9 Statistical analysis of correlation of genes/miRNAs within module………..…5

10 Hypergeometric test p-value……..………………………………….….………6

11 BH adjusted p-value……..……………………………………..…….….………6

12 Fold Enrichment (FE) …..……………….……………………..…….….………6

13 Known gene set enrichment analysis of modules…………...…….….………6

14 Cancer genes/miRNA set enrichment analysis of modules…….….…………6

15 Gene-gene interaction set enrichment analysis of modules….….….………7

16 miRNA-gene interaction set enrichment analysis of modules.….……..……8

17 Statistical significance analysis…………………………..….……..………..…8

18 Survival analysis of modules..……………….……………..…….….….………9

19 A modularity-based simulated annealing (Modularity_SA) method for discovering cancer-miRNA-gene modules……..…………………..….….………9

20 Evaluation metrics…..……………………………...……………..….….……..10

21 More details about simulation study…..……………...……………..……..…11

22 More details about comparison of TSCCA with other methods…..…………14

23 Results of gene-gene and miRNA-gene interaction set enrichment….……16

24 What are specific and shared modules? …..……………...………….………19

25 Statistical analysis and software…..……………...……………..….….……..19

26 Supplementary Figures and Tables……………...……………..….….……20

**1 Proof of the Proposition 1**

For its any feasible solution of (7) $\boldsymbol{u}$, let $I_{u}=\left\{ i | u_{i}>0, i=1,2,\cdots,p \right\}$ and $\boldsymbol{u}_{k}={\{u_{i}\}}_{i\in I_{u}}\in R^{|I_{u}|}$ and $\boldsymbol{z}_{k}={\{z_{i}\}}_{i\in I_{u}}\in R^{|I_{u}|}$, where $|I_{u}|$ is the number of elements in $I_{u}$ (Note that $|I_{u}|\leq k$). Then the objective function of (7) meets

$$\boldsymbol{|z}^{T}\boldsymbol{u|=}\left| {\boldsymbol{z}_{k}^{T}\boldsymbol{u}}_{k} \right|\boldsymbol{\leq}\left\| \boldsymbol{u}_{k} \right\|\left\| \boldsymbol{z}_{k} \right\|\boldsymbol{\leq}\left\| \boldsymbol{z}_{k} \right\|\boldsymbol{\leq}\left\| \Pi(\boldsymbol{z},k) \right\|$$

where $\left\| \Pi(\boldsymbol{z},k) \right\|$ is defined in (7). Obviously, the objective function of (7) gets the minimum value $-\left\| \Pi(\boldsymbol{z},k) \right\|$, when $I_{u}=\mathrm{support}(\boldsymbol{z},k)$ and $\boldsymbol{u}_{k}=\frac{\boldsymbol{z}_{k}}{\left\| \boldsymbol{z}_{k} \right\|}$. So, we prove that suppose $\mathbf{z}$ is a non-zero vector, then the solution of (7) is $\boldsymbol{u}^{*}=\frac{\Pi(\boldsymbol{z},k)}{\left\| \Pi(\boldsymbol{z},k) \right\|}$.

**2 Stopping condition of TSCCA Algorithm**

we develop a block-coordinate descent algorithm to solve (5). The details of this algorithm are shown in Algorithm 1. Note that $\bar{\boldsymbol{A}}$ means mathematical font ***A*** (or \mathcal{A}) for convenience in the word office software.

**Algorithm 1** TSCCA algorithm solves (5)

**Require:** $\boldsymbol{X}^{i}\in R^{n_{i}\times p}$ (gene expression data) and $\boldsymbol{Y}^{i}\in R^{n_{i}\times q}$ (miRNA expression data) for $i-1,\cdots,M$ (cancer types); Parameters: $k_{u}, k_{v}, {\mathrm{and} k}_{w}.$

**Ensure:** $\boldsymbol{u},\boldsymbol{v},\boldsymbol{w}$ and singular value$\text{ }d$.

1: Compute ${\bar{\boldsymbol{A}}}_{i}=\frac{1}{n_{i}}X^{i^{T}}Y^{i},\text{ }i=1,\cdots,M$

2: Initialize $\boldsymbol{w}={[\frac{1}{\sqrt{M}},\frac{1}{\sqrt{M}},\cdots,\frac{1}{\sqrt{M}}]}^{T}$ to ensure $\left\| w \right\|_{\text{2}}^{\text{2}}=1$

3: Initialize $\boldsymbol{u},\text{ }\boldsymbol{v}$ using the principal left and right singular vectors of $\sum_{i=1}^{M} w_{i}{\bar{\boldsymbol{A}}}_{i}$

4: **Repeat**

5: Compute $\boldsymbol{C}=\sum_{i=1}^{M} w_{i}{\bar{\boldsymbol{A}}}_{i}$

6: Let $z_{u}=\boldsymbol{Cv}$

7: $\boldsymbol{u}\leftarrow\frac{\Pi(\boldsymbol{z}_{u},k_{u})}{\left\| \Pi(\boldsymbol{z}_{u},k_{u}) \right\|}$

8: Let $\boldsymbol{z}_{v}=\boldsymbol{C}^{T}\boldsymbol{u}$

9: $\boldsymbol{v}\leftarrow\frac{\Pi(\boldsymbol{z}_{v},k_{v})}{\left\| \Pi(\boldsymbol{z}_{v},k_{v}) \right\|}$

10: Let $\boldsymbol{z}_{w}={[\boldsymbol{u}^{T}{\bar{\boldsymbol{A}}}_{1}\boldsymbol{v},\cdots,\boldsymbol{u}^{T}{\bar{\boldsymbol{A}}}_{M}\boldsymbol{v}]}^{T}$

11: $\boldsymbol{w}\leftarrow\frac{\Pi(z_{w},k_{w})}{\left\| \Pi(z_{w},k_{w}) \right\|}$

12: **Until** convergence of $\boldsymbol{u}$,$\boldsymbol{v}$ and $\boldsymbol{w}$

13: $d=\boldsymbol{w}^{T}\boldsymbol{z}_{w}$

14: **Return**$\boldsymbol{u},\boldsymbol{v},\boldsymbol{w}$ and singular value$\text{ }d$.

The stopping condition in the step 12 of Algorithm 1 is

$$\frac{\left\| \boldsymbol{u}^{i}-\boldsymbol{u}^{i-1} \right\|}{\left\| \boldsymbol{u}^{i-1} \right\|}+\frac{\left\| \boldsymbol{v}^{i}-\boldsymbol{v}^{i-1} \right\|}{\left\| \boldsymbol{v}^{i-1} \right\|}+\frac{\left\| \boldsymbol{w}^{i}-\boldsymbol{w}^{i-1} \right\|}{\left\| \boldsymbol{w}^{i-1} \right\|}<{10}^{-4}$$

where ($\boldsymbol{u}^{i}$, $\boldsymbol{v}^{i}$ and $\boldsymbol{w}^{i}$) and ($\boldsymbol{u}^{i-1}$, $\boldsymbol{v}^{i-1}$ and $\boldsymbol{w}^{i-1}$) represent the $i$th and ($i-1$)th iterations of ($\boldsymbol{u}$, $\boldsymbol{v}$ and $\boldsymbol{w}$), respectively.

**3 Convergence Analysis**

In the section, we discuss the convergence of the proposed Algorithm 1.

**Proposition 2.** Algorithm 1 decreases the objective value of (5) in each iteration.

**Proof:** Let $T_{0}=\max_{ijk}\left| \bar{A}_{ijk} \right|$. Note that $\bar{\boldsymbol{A}}$ means mathematical font ***A*** (or \mathcal{A}) for convenience in the word office software. Mathematically, the objective function of (5) can be written as follows:

$$f\left( \boldsymbol{u},\boldsymbol{v},\boldsymbol{w} \right)=-\bar{\boldsymbol{A}}\times_{1}\boldsymbol{u}\times_{2}\boldsymbol{v}\times_{3}\boldsymbol{w}\geq-\sum_{ijk} \left| \bar{A}_{ijk} \right|\cdot\left| u_{i} \right|\cdot\left| v_{j} \right|\cdot\left| w_{k} \right|$$

$\geq-\sum_{ijk} \left| {\bar{\boldsymbol{A}}}_{ijk} \right|\geq-p\cdot q\cdot M\cdot T_{0}>-\infty$.

Thus, we prove that the objective function has a lower bound. Since Eq. (7) corresponds to three simple linear problems with $\mathcal{l}_{0}$-norm constrain, we can easily get their optimal solution (**Proposition 1**). This ensures that the objective function value decreases at each iteration of Algorithm 1. In addition, **Proposition 3** can get better results for convergence Analysis.

**Proposition 3.** Let $\left\{ (u^{k},v^{k},w^{k}) \right\}$ be a sequence generated by Algorithm 1. Then the objective function value of (5) is non-increasing and $\left\{ (u^{k},v^{k},w^{k}) \right\}$ converges to a stationary point of (5).

**Proof:** Block-coordinate descent is the most popular strategy to solve Eq. (5) problem. In particular, the block prox-linear (BPL) algorithm [1] has been recognized as a computationally efficient method. The algorithm updates one block of variables at a time by minimizing a certain proximal linear projection problem. Here, we first prove Algorithm 1 is also a BPL Algorithm.

**Observation 1** The projection of a point $\boldsymbol{z}$, onto a set $R_{u}=\left\{ \boldsymbol{u} \right| \left\| \boldsymbol{u} \right\|=1, \left\| \boldsymbol{u} \right\|_{0}\leq k \}$ is defined as

$$P_{R_{u}}\left( \boldsymbol{z} \right)=\mathrm{argmin}_{u\in R_{u}}\left\| \boldsymbol{u}-\boldsymbol{z} \right\|_{2}^{2}=\frac{\Pi(\boldsymbol{z},k)}{\left\| \Pi(\boldsymbol{z},k) \right\|}$$

By apply block-coordinate descent algorithm to solve problem (5), the algorithm alternatively performs proximal gradient descent steps with respect to $\boldsymbol{u}$, $\boldsymbol{v}$ and $\boldsymbol{w}$:

$$\boldsymbol{u}^{k+1}\boldsymbol{:=}P_{R_{u}}\left( \boldsymbol{u}^{k}\boldsymbol{-}\frac{1}{L_{u}}\nabla_{u}f(\boldsymbol{u}^{k},\boldsymbol{v}^{k},\boldsymbol{w}^{k}) \right)$$

$$\boldsymbol{v}^{k+1}\boldsymbol{:=}P_{R_{u}}\left( \boldsymbol{u}^{k}\boldsymbol{-}\frac{1}{L_{v}}\nabla_{v}f(\boldsymbol{u}^{k+1},\boldsymbol{v}^{k},\boldsymbol{w}^{k}) \right)$$

$$\boldsymbol{w}^{k+1}\boldsymbol{:=}P_{R_{u}}\left( \boldsymbol{u}^{k}\boldsymbol{-}\frac{1}{L_{w}}\nabla_{w}f(\boldsymbol{u}^{k+1},\boldsymbol{v}^{k+1},\boldsymbol{w}^{k}) \right)$$

where $f\left( \boldsymbol{u},\boldsymbol{v},\boldsymbol{w} \right)=-\bar{\boldsymbol{A}}\times_{1}\boldsymbol{u}\times_{2}\boldsymbol{v}\times_{3}\boldsymbol{w}$. $L_{u}$ is the Lipschitz constant of $\nabla_{u}f\left( \boldsymbol{u}^{k},\boldsymbol{v}^{k},\boldsymbol{w}^{k} \right)=-\bar{\boldsymbol{A}}\times_{2}\boldsymbol{v}^{k}\times_{3}\boldsymbol{w}^{k}$. Since $\nabla_{u}^{2}f=0$, $L_{u}$ can be any constant greater than zero. When $L_{u}\to0$**,** based on the above Observation 1, we thus have

$$\boldsymbol{u}^{k+1}\boldsymbol{:=}P_{R_{u}}\left( \boldsymbol{u}^{k}\boldsymbol{-}\frac{1}{L_{u}}\nabla_{u}f\left( \boldsymbol{u}^{k},\boldsymbol{v}^{k},\boldsymbol{w}^{k} \right) \right)=P_{R_{u}}\left( -\bar{\boldsymbol{A}}\times_{2}\boldsymbol{v}^{k}\times_{3}\boldsymbol{w}^{k} \right)=\frac{\Pi(\boldsymbol{z}_{u},k_{u})}{\left\| \Pi(\boldsymbol{z}_{u},k_{u}) \right\|}$$

Where $\boldsymbol{z}_{u}\boldsymbol{=}\bar{\boldsymbol{A}}\boldsymbol{\times}_{\boldsymbol{2}}\boldsymbol{v}^{k}\boldsymbol{\times}_{\boldsymbol{3}}\boldsymbol{w}^{k}$**.** Similarly, we have $\boldsymbol{v}^{k+1}\boldsymbol{:=}\frac{\Pi(\boldsymbol{z}_{v},k_{v})}{\left\| \Pi(\boldsymbol{z}_{v},k_{v}) \right\|}$ and $\boldsymbol{w}^{k+1}\boldsymbol{=}\frac{\Pi(z_{w},k_{w})}{\left\| \Pi(z_{w},k_{w}) \right\|}.$ The details of this algorithm is shown in Algorithm 1. So, Algorithm 1 is also a BPL Algorithm.

Through checking the assumptions of **Theorem 2 in ref. [1]** and the above Proposition 2, we thus can obtain the conclusion of Proposition 3.

**4 Computational complexity**

We first get the complexity of the matrix multiplication on the two given matrices. The complexity of matrix multiplication with one $n \times p$ matrix and another one $n \times q$ is $O(npq)$. Thus, the computational complexity of Algorithm 1 is $O\left( pqm \right)$ at each iteration (from step 5 to 11). We set the default maximum number of iterations of algorithm 1 is 200. In fact, the convergence of TSCCA algorithm was very fast when it was applied to the TCGA dataset (S1 Fig).

**5 Parameter selection**

In TSCCA, a key issue is how to determine the parameters $k_{u}$ (i.e. the number of genes) and $k_{v}$ (i.e. the number of miRNAs) and $k_{w}$ (i.e., the number of cancer types) rank $r$ (i.e. the number of modules). In fact, how to determine these parameters of TSCCA is still an open problem.

Since mathematically maximizing the objective function of TSCCA in Eq. (5) is equivalent to minimizing $\left\| \bar{\boldsymbol{A}} - d\cdot\boldsymbol{u}\circ\boldsymbol{v}\circ\boldsymbol{w} \right\|$. Inspired by previous studies [2], $k_{u}$, $k_{v}$ and $k_{w}$ can always be chosen by minimizing the following Bayesian Information Criterion (BIC):

$$BIC (k_{u},k_{v},k_{w}) =\log\left( \frac{\left\| \bar{\boldsymbol{A}} - d\cdot\boldsymbol{u}\circ\boldsymbol{v}\circ\boldsymbol{w} \right\|}{pqM} \right)+\frac{\log(pqM)}{pqM}\{\left\| \boldsymbol{u} \right\|_{0}+\left\| \boldsymbol{v} \right\|_{0}+\left\| \boldsymbol{w} \right\|_{0}\}$$

where $\bar{\boldsymbol{A}}$ means mathematical font ***A*** (or \mathcal{A}) for convenience in the word office software. Though the parameters of Algorithm 1 can be chosen using BIC, it may not be necessary for certain biological applications. For example, we might simply fix these parameters to restrict an identified module to contain a specific number of genes, miRNAs and cancer types. In this study, based on literature review [3-5], we empirically set $k_{u}=100$, $k_{v}=10$ and $k_{w}=20$ as default values in Algorithm 1, which makes $\boldsymbol{u}$ contain about 100 non-zero genes and $\boldsymbol{v}$ contain about 10 non-zero miRNAs and $\boldsymbol{w}$ contain 20 cancer types**.** Furthermore, since we focus on identifying pan-cancer miRNA-gene modules, we expect that all identified modules cover about 1000 to 2000 genes, which is roughly the same as the number of cancer genes collected. To this end, we used **Algorithm 2**, which is detailed in the 6th section of Supplementary Materials, to identify 50 pan-cancer miRNA-gene modules which covers 1793 genes.

**6 TSCCA extracts multiple sparse factors/modules**

Because maximizing the objective function of TSCCA in Eq. (5) is equivalent to minimizing $\left\| \bar{\boldsymbol{A}} - d\cdot\boldsymbol{u}\circ\boldsymbol{v}\circ\boldsymbol{w} \right\|$. Mathematically, TSCCA model is equivalent to the rank-one sparse high-order singular value decomposition (SVD) [2]. Inspired by higher-order SVD, we also use a one-by-one way to identify multiple sparse factors. Briefly, we update the input tensor with $\bar{\boldsymbol{A}}=\bar{\boldsymbol{A}}-\boldsymbol{d}_{j}\boldsymbol{u}\circ\boldsymbol{v}\circ\boldsymbol{w}$ and re-use Algorithm 1 to identify the next module in Algorithm 2. In this study, we have applied Algorithm 2 to identify 50 cancer-miRNA-gene modules on the TCGA data (see “Parameter selection” for more detailed description).

**Algorithm 2** TSCCA extracts $r$ sparse factors/modules

**Require:** $\boldsymbol{X}^{i}\in R^{n_{i}\times p}$ (gene expression data) and $\boldsymbol{Y}^{i}\in R^{n_{i}\times q}$ (miRNA expression data) for $i-1,\cdots,M$ (cancer types); Parameters: $k_{u}, k_{v}, k_{w},\mathrm{and} r.$

**Ensure:** $\boldsymbol{U},\boldsymbol{V},\boldsymbol{W}\text{ and }\boldsymbol{d}$

1: Compute ${\bar{\boldsymbol{A}}}_{i}=\frac{1}{n_{i}}X^{i^{T}}Y^{i},\text{ }i=1,\cdots,M$

2: Let $\boldsymbol{U}\in R^{p\times r},\boldsymbol{V}\in R^{q\times r},\boldsymbol{W}\in R^{n\times r}\text{ and }\boldsymbol{d}\in R^{r\times1}$

3: **for** $j=1\text{ to }r\text{ do}$

4: Initialize $\boldsymbol{w}={[\frac{1}{\sqrt{M}},\frac{1}{\sqrt{M}},\cdots,\frac{1}{\sqrt{M}}]}^{T}$ to ensure $\left\| w \right\|_{\text{2}}^{\text{2}}=1$

5: Initialize $\boldsymbol{u},\text{ }\boldsymbol{v}$ using the principal left and right singular vectors of $\sum_{i=1}^{M} w_{i}{\bar{\boldsymbol{A}}}_{i}$

6: **Repeat**

7: Compute $\boldsymbol{C}=\sum_{i=1}^{M} w_{i}{\bar{\boldsymbol{A}}}_{i}$

8: Let $z_{u}=\boldsymbol{Cv}$

9: $\boldsymbol{u}\leftarrow\frac{\Pi(\boldsymbol{z}_{u},k_{u})}{\left\| \Pi(\boldsymbol{z}_{u},k_{u}) \right\|}$

10: Let $\boldsymbol{z}_{v}=\boldsymbol{C}^{T}\boldsymbol{u}$

11: $\boldsymbol{v}\leftarrow\frac{\Pi(\boldsymbol{z}_{v},k_{v})}{\left\| \Pi(\boldsymbol{z}_{v},k_{v}) \right\|}$

12: Let $\boldsymbol{z}_{w}={[\boldsymbol{u}^{T}{\bar{\boldsymbol{A}}}_{1}\boldsymbol{v},\cdots,\boldsymbol{u}^{T}{\bar{\boldsymbol{A}}}_{M}\boldsymbol{v}]}^{T}$

13: $\boldsymbol{w}\leftarrow\frac{\Pi(z_{w},k_{w})}{\left\| \Pi(z_{w},k_{w}) \right\|}$

14: **Until** convergence of $\boldsymbol{u}$,$\boldsymbol{v}$ and $\boldsymbol{w}$

15: $\boldsymbol{d}_{j}=\boldsymbol{w}^{T}\boldsymbol{z}_{w}$

16: $\boldsymbol{U}\left[ ,j \right]=\boldsymbol{u}, \boldsymbol{V}\left[ ,j \right]=\boldsymbol{v}, \boldsymbol{W}[,j]=\boldsymbol{w}$

17: $\bar{\boldsymbol{A}}=\bar{\boldsymbol{A}}-\boldsymbol{d}_{j}\boldsymbol{u}\circ\boldsymbol{v}\circ\boldsymbol{w}$

18: **end for**

19: **Return** $\boldsymbol{U},\boldsymbol{V},\boldsymbol{W}\text{ }\text{and }\boldsymbol{d}$

**7 Overlap analysis of any two modules using permutation test**

Since each cancer-miRNA-gene module identified by TSCCA corresponds to a sub-tensor (Fig 1C). To assess the overlap level of the two modules, we first define the overlapping elements between two modules/sub-tensors as follows: If there are two modules $\left( I_{1},J_{1},K_{1} \right)=\left\{ \left( i,j,k \right) \right| i\in I_{1},j\in J_{1}, i\in k_{1}\}$ and $\left( I_{2},J_{2},K_{2} \right)=\left\{ \left( i,j,k \right) \right| i\in I_{2},j\in J_{2}, i\in k_{2}\}$, then we defined their overlapping elements as:

$$\left( I_{1},J_{1},K_{1} \right)\bigcap\left( I_{2},J_{2},K_{2} \right)=\left\{ \left( i,j,k \right) \right| \left( i,j,k \right)\in\left( I_{1},J_{1},K_{1} \right) \mathrm{and}\left( i,j,k \right)\in\left( I_{2},J_{2},K_{2} \right) \}$$

We then define the number of overlapping elements between the module $\left( I_{1},J_{1},K_{1} \right)$ and the module $\left( I_{2},J_{2},K_{2} \right)$ as the number of the elements of the intersection $\left( I_{1},J_{1},K_{1} \right)\bigcap\left( I_{2},J_{2},K_{2} \right)$.

Based on the above definition, we adopt a permutation test method to assess the number of overlapping elements for a given pair of two identified modules $\left( I_{1},J_{1},K_{1} \right)$ and $\left( I_{2},J_{2},K_{2} \right)$:

Step 1. We first generate 1000 pairs of random modules from the original cancer-cancer-miRNA-gene correlation tensor with the same size of the given pair of modules.

Step 2. We then compute the number of overlapping elements between the given pair of modules, denoted by $b_{0}$. Moreover, we compute the number of overlapping elements between any pair of random modules, denoted by $b_{1}, \cdots,b_{1000}$.

Step 3. For the pair of two identified modules $\left( I_{1},J_{1},K_{1} \right)$ and $\left( I_{2},J_{2},K_{2} \right)$, a *p*-value is computed by using the formula:

$p-value =\frac{\sum_{t=1}^{1000} 1(b_{0} \geq b_{t})}{1000}$.

**8 Statistical analysis of modularity scores using permutation test**

For a given cancer-miRNA-gene module, we use a method based on permutation test to compute a *p*-value to evaluate the significance level of its modularity score:

Step 1. Compute its modularity score, m0, using the definition of “Modularity” in (9).

Step 2. Generate 1000 random modules from the original cancer-miRNA-gene tensor (Figure 1B) with the same number of miRNAs, genes and cancer types of the module, and their modularity values, m1, m2, …, m1000, are also calculated using (9).

**Step 3.** Combine m0 and m1, …, m1000, and compute the p-value of the module using the following formula:

$p-value =\frac{\sum_{i=1}^{1000} 1\left( m_{i} \geq m_{0} \right)+1}{1000+1}$*.*

where adding 1 to both numerator and denominator to avoid p-value of zero.

**9 Statistical analysis of correlation of genes/miRNAs within module**

For a given module, we use a permutation test method to assess the correlation of genes/miRNAs within the module as follows:

Step 1. Compute an average absolute value of the gene-gene Pearson correlation coefficients (PCCs) for the module, denoted m0;

Step 2. Generate 1000 random modules with the same number of miRNAs, genes and cancer types of the module, and compute the average absolute values of gene-gene PCCs for these random modules, denoted m1, …, m1000;

Step 3. Combine m0 and m1, …, m1000, and compute the p-value of the module using the following formula:

$p-value =\frac{\sum_{i=1}^{1000} 1\left( m_{i} \geq m_{0} \right)+1}{1000+1}$*.*

where adding 1 to both numerator and denominator to avoid p-value of zero.

Similarly, we also apply the above procedure to assess the co-expressed level between these miRNAs within a given module.

**10 Hypergeometric test *p*-value**

The right-tailed hypergeometric test has been used several times for assessing overlapping level between two sets in this study. We consider a big set S and its two sub-sets S1 and S2 with N, n1 and n2 (number of draws) elements respectively. We use a one-tailed Hypergeometric test to calculate the *p*-value of *k* overlapping elements between S1 and S2 by the following formula:

$$p\text{(}k\text{)}=1-\sum_{i=0}^{k-1} p(x=i)$$

where the probability that there are *k* overlapping elements is $p(x=k)=\frac{\left( \begin{aligned} n1 \\ x \end{aligned} \right)\left( \begin{aligned} N-n1 \\ n2-x \end{aligned} \right)}{\left( \begin{aligned} N \\ n2 \end{aligned} \right)}$,

and $\left( \begin{aligned} n \\ k \end{aligned} \right)=n!/(k!\left( n-k \right)!)$ is a binomial coefficient.

**11 BH adjusted *p*-value**

The Benjamini-Hochberg (BH) procedure is a powerful tool that decreases the false discovery rate in the statistical multiple hypothesis testing. The resulting *p*-values are corrected for multiple testing using the BH method [6].

**12 Fold enrichment (FE)**

FE = A/B, where A is the observed value and B is the expected value. It is used several times in S10 Table.

**13 Known gene set enrichment analysis of modules**

For a gene set in a given module, we perform functional enrichment analysis for each known KEGG and GOBP gene set using the right-tailed hypergeometric test (10th section of Supplementary Materials), which is implemented via “phyper” function in R software. After multiple testing correction via the BH method, we obtain the BH adjusted *p*-values. Finally, only those known KEGG and GOBP terms with a **BH adjusted *p*-value** < 0.05 are selected as significant ones.

**14 Cancer genes/miRNA set enrichment analysis of modules**

We employ the right-tailed hypergeometric test (See section 10 in S1 Text) to compute a significance level to measures whether these genes from the same module are significantly overlapped with the cancer genes. Similarly, we can compute a significance level of genes within a module.

**15 Gene-gene interaction set enrichment analysis of modules**

**15.1 Permutation test**

For a given module $i$, suppose it contains $n_{0}$ genes and $m_{0}$ validated gene interactions/edges and the sum of degrees of genes within the module in the original gene-gene interaction network is $d_{0}$.

Step 1. We first randomly generate 1000 modules by permuting gene names only between genes with the same degree, so as to eliminate the influence of the vertex degree on the result.

Step 2. We then computed the number of validated gene interactions of these random modules, denoted as $m_{1}, m_{2}, \ldots, m_{1000}$.

Step 3. For the given module $i$ and its *p*-value was computed by using the formula:

$p-value =\frac{\sum_{i=1}^{1000} 1\left( m_{i} \geq m_{0} \right)+1}{1000+1}$.

where adding 1 to both numerator and denominator to avoid *p*-value of zero.

**15.2 Hypergeometric test**

For a given module $i$, suppose it contains $n_{i}$ genes and $m_{i}$ validated gene interactions or edges. We employ the right-tailed hypergeometric test (See section 10 in S1 Text) for gene interaction set enrichment analysis of the module with the following steps:

Step 1. Define the following variables:

- $N=\left( \begin{aligned} n \\ 2 \end{aligned} \right)$ denotes the number of all possible gene-gene pairs for all considered genes;
- $n1$denotes the number of gene-gene interactions in the gene interaction network;
- $n2=\left( \begin{aligned} n_{i} \\ 2 \end{aligned} \right)$denotes the number of all possible gene-gene pairs for the module $i$.

Step 2. Compute a *p*-value for the given module by the following formula:

$p-value\text{(}k=m_{i}\text{)}=1-\sum_{i=0}^{k-1} p(x=i)$ where $p(x=k)=\frac{\left( \begin{aligned} n1 \\ x \end{aligned} \right)\left( \begin{aligned} N-n1 \\ n2-x \end{aligned} \right)}{\left( \begin{aligned} N \\ n2 \end{aligned} \right)}$.

**16 miRNA-gene interaction set enrichment analysis of modules**

**16.1 Permutation test**

For a given module $i$, suppose it contains $n_{0}$ genes and $m_{0}$ validated miRNA-gene interactions/edges. And the sum of degrees of miRNAs within the module in the original miRNA-gene interaction network is $d_{0}$.

Step 1. We first randomly generate 1000 miRNA-gene modules by permuting gene/miRNA names only between genes/miRNAs with the same degree, so as to eliminate the influence of the degree on the result.

Step 2. We then compute the number of validated miRNA-gene interactions of these random modules, denoted as $m_{1}, m_{2}, \ldots, m_{1000}$*.*

Step 3. For the given module $i$ and its p-value is computed by using the formula:

$p-value =\frac{\sum_{i=1}^{1000} 1\left( m_{i} \geq m_{0} \right)+1}{1000+1}$*.*

where adding 1 to both numerator and denominator to avoid p-value of zero.

**16.2 Hypergeometric test**

For a given module $i$, suppose it contains $p_{i}$ miRNAs, $q_{i}$ genes and $k_{i}$ experimentally validated miRNA-gene interactions. We also employ the right-tailed hypergeometric test for miRNA-gene interaction set enrichment analysis of the module with the following steps:

Step 1. Define the following variables:

- $N= p*q$: where $p$ ($q$*)* are the number of all considered genes (miRNAs);
- $n1$denotes the number of the experimentally validated miRNA-gene interactions from miRTarBase database;
- ${n2=p}_{i}\cdot q_{i}$ denotes the number of all possible miRNA-gene pairs for the module $i$;

Step 2. Compute a *p*-value by the following formula:

$p-\mathrm{value}\text{(}k=k_{i}\text{)}=1-\sum_{i=0}^{k-1} p\left( x=i \right),$ where $p(x=k)=\frac{\left( \begin{aligned} n1 \\ x \end{aligned} \right)\left( \begin{aligned} N-n1 \\ n2-x \end{aligned} \right)}{\left( \begin{aligned} N \\ n2 \end{aligned} \right)}$.

**17 Statistical significance analysis**

For a given module set {M1, M2, …, M50}, we adopt a permutation test method to evaluate the significance of the number of modules from the module set with at least two miRNAs from the same family.

Step 1. Compute the number of modules, $n_{0}$, from the given module set with at least two miRNAs from the same family.

Step 2. We generate 1000 random module sets with the same size of the given set {M1, M2, …, M50} from the original cancer-miRNA-gene correlation tensor.

Step 3. For a random module set $i \left( i=1,2,\ldots,1000 \right)$, compute the number of modules, $n_{i}$, from the given module set with at least two miRNAs from the same family.

Step 4. Combine $n_{0}$ and $n_{1}, \ldots, n_{1000}$*,* and compute the p-value of the given module set using the following formula:

$p-value =\frac{\sum_{i=1}^{1000} 1\left( n_{i} \geq n_{0} \right)+1}{1000+1}$*.*

where adding 1 to both numerator and denominator to avoid *p*-values of zero.

Similarly, we also apply the above procedure to evaluate the significance of 70% of the modules, where each module has at least 3 miRNAs participating in a three-layer network.

**18 Survival analysis of modules**

**18.1 Survival analysis of module expression**

To assess whether the expression of both miRNAs and genes within the module is related to the patient survival time, we develop a procedure for each cancer type within the module as follows:

Step1. Obtain an expression matrix of miRNAs/genes in the module, namely, a module expression matrix.

Step 2. Extract the first principal component (PC1) as the prognostic scores based on the expression data of these genes and miRNAs for each cancer within the module.

Step 3. Divide the cancer samples into two groups based the median value of the prognostic scores and log-rank test is used for the two groups and a *p*-value is computed and a Kaplan-Meier (KM) curve is draw for visualization.

Step 4. These resulting *p*-values are corrected using the BH method.

**18.2 Survival analysis of single miRNA expression**

Additionally, we also consider the expression of each miRNA in a module as the prognostic scores. Similarly, the *p*-values and BH adjusted *p*-values are computed using the above procedure.

**19 A modularity-based simulated annealing (Modularity_SA) method for discovering cancer-miRNA-gene modules**

Inspired by previous studies [5, 7], we develop a modularity-based simulated annealing method (modularity_SA) to identified modules. The method uses a simulated annealing algorithm to maximize the modularity index (9) for extracting a cancer-miRNA-gene module. The R code of Modularity_SA function is showed as follows:

SA.modularity = function(Tensor, ku, kv, kw, niter=10000, seed0=1){

dat.dim = dim(Tensor) # miRNAs*genes*cancers

# Initialization: miR.x gene.x cancer.x

set.seed(seed0)

x1 = sample(1:dat.dim[1], ku, replace=F)

x2 = sample(1:dat.dim[2], kv, replace=F)

x3 = sample(1:dat.dim[3], kw, replace=F)

obj = c()

for(k in 1:niter){

T0 = niter/k # Temperature

# get.SA.newSolution

# Randomly choose to update two miRNAs, or two genes or tow cancers

set.seed(seed0*k) # set random seed

if(kw<dat.dim[3]){

flg = sample(c(1,2,3),1)

}else{flg = sample(c(1,2),1)}

Threshold.p = runif(1,0,1)

if(flg==1){

i = sample(x1,1)

xx = 1:dat.dim[1]

j = sample(xx[-c(x1)],1) # update point

deta.E = sum(abs(Tensor[j,x2,x3])) - sum(abs(Tensor[i,x2,x3]))

if(exp(deta.E/T0)>=Threshold.p){

x1 = c(x1,j)

x1 = x1[-which(x1==i)]}

}else if(flg==2){

i = sample(x2,1)

xx = 1:dat.dim[2]

j = sample(xx[-c(x2)],1)

deta.E = sum(abs(Tensor[x1,j,x3])) - sum(abs(Tensor[x1,i,x3]))

if(exp(deta.E/T0)>=Threshold.p){

x2 = c(x2,j)

x2 = x2[-which(x2==i)]}

}else{

i = sample(x3,1)

xx = 1:dat.dim[3]

j = sample(xx[-c(x3)],1)

deta.E = sum(abs(Tensor[x1,x2,j])) - sum(abs(Tensor[x1,x2,i]))

if(exp(deta.E/T0)>=Threshold.p){

x3 = c(x3,j)

x3 = x3[-which(x3==i)]

}

}

obj = c(obj,mean(abs(Tensor[x1,x2,x3])))

}

return(list(u=sort(x1), v=sort(x2), w=sort(x3),obj=obj))

}

**20 Evaluation metrics**

In the section, we use two evaluation metrics to assess module or subspace clusters, including recovery score [8] and clustering error (CE) score [9]. We also extend the “Recovery score” to evaluate the similarity of two tri-clusters, bi-clusters, and clusters. Their ranges are between 0 (low quality) and 1 (perfect).

**20.1 Recovery score**

Let $M^{True}$ and $M^{Pre}$ be two sets of modules, where $M^{True}$ denotes the set of the $k$ true modules and $M^{Pre}$ denotes the set of the $l$ predicted modules.

(i) 3D Recovery score for two tri-clusters

$$3D Recovery =\frac{1}{k}\cdot\sum_{i=1}^{k} \max_{1\leq j\leq l} Jaccard\left( M_{i}^{True},M_{j}^{Pre} \right)$$

where $M_{i}^{True}$and $M_{j}^{Pre}$ are two subtensors/tri-clusters and $Jaccard$ is defined as

$$Jaccard\left( M_{i}^{True},M_{j}^{Pre} \right)=\frac{\left| \left\{ C_{ijk} | C_{ijk}\in M_{i}^{True} \mathrm{and} C_{ijk}\in M_{j}^{Pre} \right\} \right|}{\left| \left\{ C_{ijk} | C_{ijk}\in M_{i}^{True} \mathrm{or} C_{ijk}\in M_{j}^{Pre} \right\} \right|}$$

(ii) 2D Recovery score for two bi-clusters

$$2D Recovery =\frac{1}{k}\cdot\sum_{i=1}^{k} \max_{1\leq j\leq l} Jaccard\left( M_{i}^{True},M_{j}^{Pre} \right)$$

where $M_{i}^{True}$and $M_{j}^{Pre}$ are two matrices/bi-clusters and Jaccard index is defined as

$$Jaccard\left( M_{i}^{True},M_{j}^{Pre} \right)=\frac{\left| \left\{ C_{ij} | C_{ij}\in M_{i}^{True} \mathrm{and} C_{ij}\in M_{j}^{Pre} \right\} \right|}{\left| \left\{ C_{ij} | C_{ij}\in M_{i}^{True} \mathrm{or} C_{ij}\in M_{j}^{Pre} \right\} \right|}$$

(iii) 1D Recovery score for two clusters

$$1D Recovery =\frac{1}{k}\cdot\sum_{i=1}^{k} \max_{1\leq j\leq l} Jaccard\left( M_{i}^{True},M_{j}^{Pre} \right)$$

where $M_{i}^{True} \mathrm{and} M_{j}^{Pre}$are two clusters and Jaccard index is defined as

$$Jaccard\left( M_{i}^{True},M_{j}^{Pre} \right)=\frac{\left| \left\{ C_{i} | C_{i}\in M_{i}^{True} \mathrm{and} C_{i}\in M_{j}^{Pre} \right\} \right|}{\left| \left\{ C_{i} | C_{i}\in M_{i}^{True} \mathrm{or} C_{i}\in M_{j}^{Pre} \right\} \right|}$$

**20.2 Clustering error (CE) score**

An intuitive way to compare the clustering results is to calculate the clustering error (CE). It is the proportion of points which are clustered differently in $M^{True}$ and $M^{Pre}$ after an optimal matching of clusters [9]. Let $\left\{ (t_{i},y_{i}) \right\}_{i=1}^{min\{k, l\}}$ be a unique relation that maximizes $d_{max}\triangleq\sum_{i=1}^{min\{k, l\}} |M_{t_{i}}^{True}\cap M_{y_{i}}^{Pre}|$. Then, the CE score is given by

$$\mathrm{CE}=1-\frac{d_{max}}{|U|}$$

where $\left| U \right|=|\{\bigcup_{i=1}^{k} M_{i}^{True}\}\cup\{\bigcup_{j=1}^{l} M_{j}^{pre}\}|$. Similarly, we also extend the “CE score” to evaluate the similarity of two tri-clusters, bi-clusters or clusters.

(i) 3D CE score for two tri-clusters

$$3D\mathrm{CE} =1-\frac{d_{max}}{|U|}$$

where $\left| U \right|=|\{\bigcup_{i=1}^{k} M_{i}^{True}\}\cup\{\bigcup_{j=1}^{l} M_{j}^{pre}\}|$ and $M_{i}^{True}$and $M_{j}^{Pre}$ are two subtensors/tri-clusters.

(ii) 2D CE score for two bi-clusters

$$2D\mathrm{CE} =1-\frac{d_{max}}{|U|}$$

where $\left| U \right|=|\{\bigcup_{i=1}^{k} M_{i}^{True}\}\cup\{\bigcup_{j=1}^{l} M_{j}^{pre}\}|$ and $M_{i}^{True}$and $M_{j}^{Pre}$ are two matrices/bi-clusters.

(iii) 1D CE score for two clusters

$$1D\mathrm{CE} =1-\frac{d_{max}}{|U|}$$

where $\left| U \right|=|\{\bigcup_{i=1}^{k} M_{i}^{True}\}\cup\{\bigcup_{j=1}^{l} M_{j}^{pre}\}|$ and $M_{i}^{True}$and $M_{j}^{Pre}$ are two clusters.

**2****1 More details about simulation study**

In this section, we compared TSCCA with SCCA and Modularity_SA on a set of simulated data. Fig 8B in the main text have already showed some results and more details are given in the S20 and S21 Tables. These results show that TSCCA is superior to other methods in terms of CE and recovery scores.

Table S20. Comparison (in terms of CE ± std) on the simulated data. Since SCCA cannot select cancer types, we assumed each module identified by SCCA contained all cancer types for computing 3D-CE and 3D-recovery score.

| Method | 1D-CE ± std  (gene) | 1D-CE ± std  (miR) | 2D-CE ± std | 3D-CE ±  std |
| --- | --- | --- | --- | --- |
| SCCA + cancer1 | 0.777±0.013 | 0.775±0.041 | 0.575±0.012 | 0.327±0.005 |
| SCCA + cancer2 | 0.777±0.012 | 0.781±0.052 | 0.576±0.014 | 0.328±0.006 |
| SCCA + cancer3 | 0.779±0.014 | 0.787±0.042 | 0.579±0.014 | 0.329±0.005 |
| SCCA + cancer4 | 0.363±0.015 | 0.421±0.039 | 0.090±0.009 | 0.058±0.006 |
| SCCA + JointData | 0.370±0.015 | 0.429±0.039 | 0.090±0.009 | 0.058±0.006 |
| Modularity_SA | 0.596±0.159 | 0.653±0.202 | 0.506±0.153 | 0.506±0.153 |
| TSCCA | **0.928**±**0.106** | **0.929**±**0.106** | **0.864**±**0.200** | **0.848**±**0.225** |

Table S21. Comparison (in terms of Recovery ± std) on the simulated data.

| Method | 1D-recovery ±std (gene) | 1D-recovery  ±std (miR) | 2D-recovery  ±std | 3D-recovery  ±std |
| --- | --- | --- | --- | --- |
| SCCA + cancer1 | 0.745±0.006 | 0.765±0.019 | 0.695±0.006 | 0.351±0.003 |
| SCCA + cancer2 | 0.744±0.007 | 0.775±0.027 | 0.696±0.006 | 0.352±0.004 |
| SCCA + cancer3 | 0.744±0.005 | 0.770±0.026 | 0.696±0.007 | 0.352±0.004 |
| SCCA + cancer4 | 0.229±0.010 | 0.300±0.038 | 0.086±0.010 | 0.055±0.006 |
| SCCA + JointData | 0.232±0.009 | 0.300±0.032 | 0.084±0.008 | 0.055±0.005 |
| Modularity_SA | 0.770±0.019 | 1.000±0.000 | 0.770±0.019 | 0.770±0.019 |
| TSCCA | **0.918**±**0.120** | **0.926**±**0.110** | **0.903**±**0.143** | **0.898**±**0.151** |

Next, we first review the process of generating simulation data. We generated a synthetic miRNA-gene correlation tensor $\bar{\boldsymbol{A}}\in R^{300\times30\times4}$with 300 genes and 30 miRNAs and 4 cancers, where


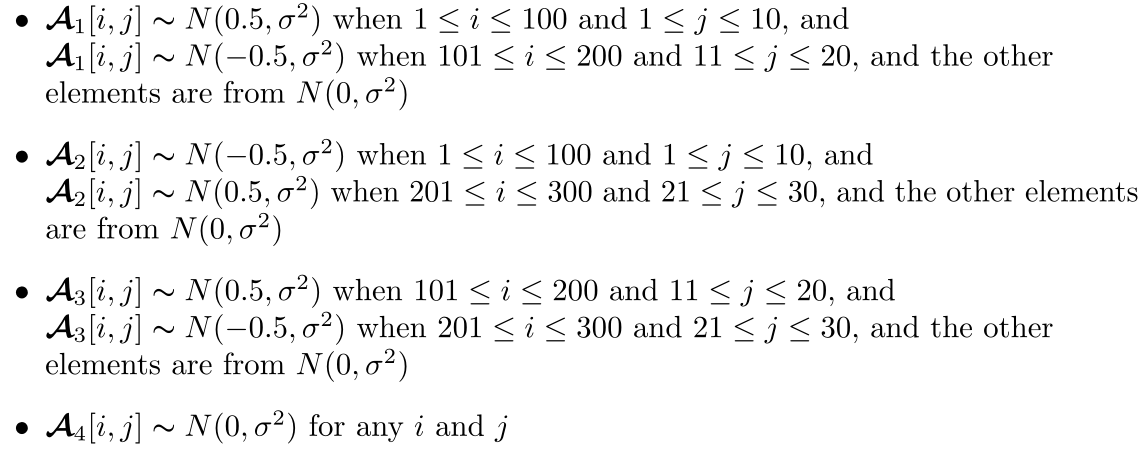


Moreover, we repeatedly generated 50 tensors ($\bar{\boldsymbol{A}}$s) with give variance $\sigma^{2}$.

Further, we considered the variance ($\sigma^{2}$) with different values including 0.1, 0.3, 0.5 and 1. We then re-applied TSCCA and SCCA to these simulated data with these different variances. Similarly, we found that TSCCA is superior to other methods in terms of CE and recovery scores (S22 and S23 Tables).

Table S22. Comparison (in terms of CE ± std) on the simulated data with different variances. Since SCCA cannot select cancer types, we assumed each module identified by SCCA contained all cancer types for computing 3D-CE.

| variance = 0.1 | 1D-CE (gene) | 1D-CE (miR) | 2D-CE | 3D-CE |
| --- | --- | --- | --- | --- |
| cancer1 | 0.769±0.013 | 0.779±0.041 | 0.567±0.014 | 0.323±0.005 |
| cancer2 | 0.768±0.011 | 0.778±0.052 | 0.565±0.015 | 0.322±0.006 |
| cancer3 | 0.770±0.014 | 0.786±0.044 | 0.569±0.015 | 0.324±0.006 |
| cancer4 | 0.363±0.015 | 0.421±0.039 | 0.090±0.009 | 0.058±0.006 |
| Sum | 0.370±0.015 | 0.429±0.039 | 0.090±0.009 | 0.058±0.006 |
| Modularity_SA | 0.582±0.142 | 0.667±0.202 | 0.468±0.137 | 0.468±0.137 |
| TSCCA | **0.897±0.113** | **0.898±0.115** | **0.805±0.214** | **0.785±0.236** |
| variance = 0.3 | **1D-CE (gene)** | **1D-CE (miR)** | **2D-CE** | **3D-CE** |
| cancer1 | 0.682±0.018 | 0.777±0.041 | 0.466±0.014 | 0.273±0.007 |
| cancer2 | 0.676±0.041 | 0.768±0.062 | 0.458±0.042 | 0.269±0.023 |
| cancer3 | 0.680±0.017 | 0.784±0.045 | 0.465±0.018 | 0.272±0.009 |
| cancer4 | 0.363±0.015 | 0.421±0.039 | 0.090±0.009 | 0.058±0.006 |
| Sum | 0.370±0.015 | 0.429±0.039 | 0.090±0.009 | 0.058±0.006 |
| Modularity_SA | 0.482±0.085 | 0.625±0.184 | 0.307±0.085 | 0.306±0.086 |
| TSCCA | **0.802±0.098** | **0.832±0.109** | **0.637±0.175** | **0.609±0.193** |
| variance = 0.5 | **1D-CE (gene)** | **1D-CE (miR)** | **2D-CE** | **3D-CE** |
| cancer1 | 0.595±0.050 | 0.753±0.083 | 0.372±0.061 | 0.222±0.035 |
| cancer2 | 0.597±0.039 | 0.761±0.074 | 0.374±0.046 | 0.224±0.025 |
| cancer3 | 0.603±0.040 | 0.765±0.071 | 0.380±0.045 | 0.227±0.025 |
| cancer4 | 0.363±0.015 | 0.421±0.039 | 0.090±0.009 | 0.058±0.006 |
| Sum | 0.370±0.015 | 0.429±0.039 | 0.090±0.009 | 0.058±0.006 |
| Modularity_SA | 0.434±0.053 | 0.575±0.122 | 0.207±0.051 | 0.197±0.053 |
| TSCCA | **0.688±0.076** | **0.752±0.094** | **0.467±0.108** | **0.434±0.116** |
| variance = 1.0 | **1D-CE (gene)** | **1D-CE (miR)** | **2D-CE** | **3D-CE** |
| cancer1 | 0.463±0.046 | 0.633±0.104 | 0.215±0.059 | 0.134±0.035 |
| cancer2 | 0.463±0.042 | 0.637±0.111 | 0.214±0.057 | 0.134±0.034 |
| cancer3 | 0.470±0.037 | 0.654±0.081 | 0.222±0.043 | 0.138±0.026 |
| cancer4 | 0.363±0.015 | 0.421±0.039 | 0.090±0.009 | 0.058±0.006 |
| Sum | 0.370±0.015 | 0.429±0.039 | 0.090±0.009 | 0.058±0.006 |
| Modularity_SA | 0.373±0.017 | 0.455±0.051 | 0.113±0.018 | 0.090±0.023 |
| TSCCA | 0.424±0.078 | 0.507±0.114 | 0.155±0.089 | 0.106±0.095 |

Table S23. Comparison of the (in terms of recovery ± std) on the simulated data with different variables. Since SCCA cannot select cancer types, we assumed each module identified by SCCA contained all cancer types for computing 3D-recovery.

| variance = 0.1 | 1D-recovery (gene) | 1D-recovery (miR) | 2D-recovery | 3D-recovery |
| --- | --- | --- | --- | --- |
| cancer1 | 0.728±0.009 | 0.768±0.022 | 0.680±0.009 | 0.346±0.005 |
| cancer2 | 0.726±0.010 | 0.773±0.026 | 0.679±0.009 | 0.346±0.005 |
| cancer3 | 0.728±0.009 | 0.770±0.026 | 0.681±0.011 | 0.347±0.005 |
| cancer4 | 0.229±0.010 | 0.300±0.038 | 0.086±0.010 | 0.055±0.006 |
| Sum | 0.232±0.009 | 0.300±0.032 | 0.084±0.008 | 0.055±0.005 |
| Modularity_SA | 0.694±0.021 | 1.000±0.000 | 0.694±0.021 | 0.694±0.021 |
| TSCCA | **0.882±0.130** | **0.894±0.117** | **0.860±0.153** | **0.854±0.160** |
| variance = 0.3 | **1D-recovery (gene)** | **1D-recovery (miR)** | **2D-recovery** | **3D-recovery** |
| cancer1 | 0.578±0.019 | 0.766±0.021 | 0.530±0.018 | 0.285±0.008 |
| cancer2 | 0.572±0.033 | 0.766±0.047 | 0.522±0.047 | 0.282±0.023 |
| cancer3 | 0.572±0.019 | 0.770±0.026 | 0.525±0.021 | 0.284±0.010 |
| cancer4 | 0.229±0.010 | 0.300±0.038 | 0.086±0.010 | 0.055±0.006 |
| Sum | 0.232±0.009 | 0.300±0.032 | 0.084±0.008 | 0.055±0.005 |
| Modularity_SA | 0.481±0.022 | 0.948±0.062 | 0.463±0.037 | 0.463±0.037 |
| TSCCA | **0.748±0.110** | **0.823±0.113** | **0.711±0.133** | **0.702±0.139** |
| variance = 0.5 | **1D-recovery (gene)** | **1D-recovery (miR)** | **2D-recovery** | **3D-recovery** |
| cancer1 | 0.467±0.033 | 0.739±0.078 | 0.408±0.059 | 0.230±0.030 |
| cancer2 | 0.468±0.025 | 0.752±0.063 | 0.412±0.046 | 0.233±0.022 |
| cancer3 | 0.471±0.032 | 0.751±0.051 | 0.416±0.044 | 0.235±0.022 |
| cancer4 | 0.229±0.010 | 0.300±0.038 | 0.086±0.010 | 0.055±0.006 |
| Sum | 0.232±0.009 | 0.300±0.032 | 0.084±0.008 | 0.055±0.005 |
| Modularity_SA | 0.358±0.030 | 0.680±0.111 | 0.274±0.051 | 0.274±0.051 |
| TSCCA | **0.592±0.081** | **0.743±0.097** | **0.536±0.102** | **0.524±0.108** |
| variance = 1.0 | **1D-recovery (gene)** | **1D-recovery (miR)** | **2D-recovery** | **3D-recovery** |
| cancer1 | 0.322±0.039 | 0.560±0.119 | 0.220±0.062 | 0.134±0.035 |
| cancer2 | 0.321±0.037 | 0.563±0.119 | 0.219±0.059 | 0.133±0.033 |
| cancer3 | 0.329±0.028 | 0.579±0.093 | 0.228±0.045 | 0.139±0.025 |
| cancer4 | 0.229±0.010 | 0.300±0.038 | 0.086±0.010 | 0.055±0.006 |
| Sum | 0.232±0.009 | 0.300±0.032 | 0.084±0.008 | 0.055±0.005 |
| Modularity_SA | 0.247±0.017 | 0.363±0.064 | 0.114±0.023 | 0.105±0.030 |
| TSCCA | 0.292±0.076 | 0.407±0.144 | 0.163±0.104 | 0.124±0.114 |

22 **More details about** comparison of TSCCA with other methods

**22.1 Comparison of TSCCA with SCCA**

For comparison with TSCCA, we first applied SCCA to identify 50 modules on each cancer data set (S24 Table). Based on the results in S24 Table, compared with SCCA when applying to each cancer data, TSCCA has some advantages in multiple biological indicators, such as the average number of cancer miRNAs, cancer genes, gene edges and miRNA-gene edges. On the other hand, for a single cancer data, SCCA did ensure that the expression of miRNAs and genes within the identified module is correlated/co-expressed in the specific cancer data (Eighth column of S24 Table), but it failed to ensure that the miRNAs and genes with the identified module are correlated in most cancer types (Seventh column of S24 Table). In short, the average modularity and multiple biological indicators of the modules identified by TSCCA are better than these of SCCA.

Table S24. Performance comparison of TSCCA and SCCA, where we used SCCA to identify 50 modules on each cancer data set. “#cancer miR”, “#cancer gene”, “#gene edge” and “#miR-gene edge” denote the average of the number of cancer miRNAs, cancer genes, gene edges and miRNA-gene edges on all the identified modules. Since SCCA cannot select cancer types when applying to single cancer data, we assumed “Modularity” of SCCA is computed on all 33 cancer types, while “Single cancer modularity” of SCCA is computed on a cancer type.

| Data | Method | #cancer  miR | #cancer  gene | #gene edge | #miR-gene edge | modularity | single cancer  modularity |
| --- | --- | --- | --- | --- | --- | --- | --- |
| 33 cancers | **TSCCA** | **5.98** | **19.56** | **106.10** | **22.70** | **0.30** |  |
| ACC | SCCA | 5.10 | 17.96 | 74.30 | 14.72 | 0.15 | 0.43 |
| BLCA | SCCA | 5.92 | 20.54 | 62.84 | 13.22 | 0.21 | 0.43 |
| BRCA | SCCA | 4.44 | 18.36 | 87.86 | 21.22 | 0.19 | 0.40 |
| CESC | SCCA | 5.00 | 18.42 | 48.90 | 14.20 | 0.18 | 0.33 |
| CHOL | SCCA | 5.26 | 19.04 | 53.14 | 14.38 | 0.16 | 0.44 |
| COAD | SCCA | 5.66 | 17.70 | 43.12 | 15.30 | 0.20 | 0.49 |
| COADREAD | SCCA | 5.54 | 17.60 | 44.32 | 14.92 | 0.20 | 0.47 |
| DLBC | SCCA | 5.04 | 16.52 | 55.70 | 16.96 | 0.14 | 0.47 |
| ESCA | SCCA | 5.46 | 16.52 | 48.36 | 15.14 | 0.18 | 0.47 |
| HNSC | SCCA | 5.94 | 18.24 | 80.16 | 20.32 | 0.19 | 0.36 |
| KICH | SCCA | 5.80 | 17.72 | 46.24 | 11.22 | 0.16 | 0.52 |
| KIPAN | SCCA | 6.26 | 17.96 | 40.62 | 15.32 | 0.17 | 0.53 |
| KIRC | SCCA | 6.42 | 19.42 | 56.20 | 17.68 | 0.18 | 0.35 |
| KIRP | SCCA | 5.86 | 17.98 | 51.22 | 13.48 | 0.18 | 0.39 |
| LGG | SCCA | 5.54 | 13.40 | 54.74 | 10.42 | 0.14 | 0.53 |
| LIHC | SCCA | 5.54 | 20.60 | 78.40 | 15.42 | 0.18 | 0.43 |
| LUAD | SCCA | 5.58 | 18.24 | 100.68 | 21.16 | 0.19 | 0.35 |
| LUSC | SCCA | 6.44 | 21.64 | 72.36 | 20.62 | 0.20 | 0.34 |
| MESO | SCCA | 6.02 | 19.60 | 61.96 | 12.40 | 0.16 | 0.39 |
| OV | SCCA | 5.66 | 19.16 | 63.58 | 19.12 | 0.18 | 0.29 |
| PAAD | SCCA | 6.22 | 20.12 | 55.64 | 13.54 | 0.16 | 0.46 |
| PCPG | SCCA | 6.10 | 18.72 | 54.20 | 15.18 | 0.17 | 0.44 |
| PRAD | SCCA | 6.96 | 17.04 | 49.34 | 21.14 | 0.19 | 0.40 |
| READ | SCCA | 5.52 | 17.52 | 43.70 | 16.32 | 0.19 | 0.46 |
| SARC | SCCA | 5.48 | 15.76 | 54.42 | 12.36 | 0.16 | 0.47 |
| SKCM | SCCA | 5.58 | 19.92 | 61.96 | 13.86 | 0.18 | 0.34 |
| STAD | SCCA | 5.68 | 17.26 | 58.28 | 19.38 | 0.22 | 0.53 |
| STES | SCCA | 5.66 | 17.28 | 65.94 | 18.28 | 0.22 | 0.47 |
| TGCT | SCCA | 5.36 | 18.30 | 37.84 | 12.86 | 0.16 | 0.66 |
| THCA | SCCA | 6.28 | 19.40 | 46.04 | 14.52 | 0.17 | 0.46 |
| UCEC | SCCA | 5.16 | 19.36 | 88.20 | 19.50 | 0.19 | 0.32 |
| UCS | SCCA | 6.20 | 15.92 | 42.22 | 10.32 | 0.15 | 0.46 |
| UVM | SCCA | 6.06 | 16.76 | 41.72 | 11.34 | 0.15 | 0.48 |

**22.2 Comparison of TSCCA with tri-clustering methods**

In this subsection, we compared TSCCA with multiple tri-clustering methods including modularity_SA and Sparse Canonical Polyadic decomposition (SCP) which uses l1 regularization to force sparse [10], and two merit-function based methods including “Variance” and “Mean squared residue (MSR)” [11]. In this study, these two merit-functions are optimized by using annealing algorithm.

The comparison results are given in the S25 Table and show that TSCCA is superior to the other methods on multiple biological indicators. Due to the definition of MSR, the MSR_SA method is very consuming time. We found that MSR_SA takes an hour to identify a module, while Var _SA only takes 5 seconds on a personal computer. Compared with the TSCCA and Modularity_ SA, the sub-tensors corresponding to the modules identified by these two methods tend to be close to zero (S6 Fig). In short, the TSCCA method is superior to other triclustering methods in multiple biological indicators.

Table S25. Performance comparison of TSCCA and the triclustering methods. “#cancer miR”, “#cancer gene”, “#gene edge” and “#miR-gene edge” denote the average of the number of cancer miRNAs, cancer genes, gene edges and miRNA-gene edges on all the identified modules.

| Method | #cancer_miR | #cancer_gene | #gene edge | #miR-gene edge | Modularity |
| --- | --- | --- | --- | --- | --- |
| TSCCA | 5.98 | 19.56 | 106.10 | 22.70 | 0.30 |
| SCP | 5.80 | 12.54 | 67.98 | 14.92 | 0.29 |
| Modularity_SA | 8.08 | 21.14 | 47.52 | 11.62 | 0.29 |
| Var_SA | 3.40 | 13.58 | 32.04 | 4.94 | 0.04 |
| MSR_SA | 3.48 | 12.30 | 32.22 | 7.14 | 0.06 |

**22.3 Comparison between TSCCA and Modularity_SA on the TCGA data in terms of modularity score**

Based on the TCGA data, we generated a joint gene expression and miRNA expression data: $X = \left[ X_{1},X_{2},\ldots，X_{33} \right]\in R^{p\times\left( n1+\cdots+n33 \right)}$ and $Y = \left[ Y_{1},Y_{2},\ldots，Y_{33} \right]\in R^{q\times\left( n1+\cdots+n33 \right)}$ (referred to as “JointData” data). Since we don't know the true modules on TCGA data, we cannot rely on the CE or Recovery scores to judge the superiority of different methods. We used the modularity score (Eq. 9) to judge the quality of the tested methods. We applied TSCCA and Modularity_SA to identify the first module on the TCGA data with 50 different initializations. To make a fair comparison of TSCCA and SCCA, we also applied SCCA to each single cancer data ($X^{i}$ and $Y^{i}$) and the “JointData” data ($X$ and $Y$). The parameters of SCCA and Modularity_SA are consistent with the parameters of TSCCA with$k_{u}=200$, $k_{v}=10$ and $k_{w}=20$. Compared with SCCA and Modularity_SA, TSCCA obtained higher modularity scores (S7A Fig). We also compared the running time of different methods on a personal laptop. TSCCA took about 12 seconds to identify a module on average, while SCCA took the least time (S7B Fig).

**22.4 Comparison of TSCCA with PCA**

In this subsection, we compared TSCCA with PCA. To this end, we first obtained the joint gene expression and miRNA expression data: $X = [X_{1},X_{2},\ldots，X_{33}]\in R^{p\times(n1+\cdots+n33)}$ and $Y = [Y_{1},Y_{2},\ldots，Y_{33}]\in R^{q\times(n1+\cdots+n33)}$. We then extracted the 1ST PC (denoted as ***u***) using the gene expression $X$ and the 1ST PC (denoted as ***v***) using the miRNA expression $Y$. For comparison with TSCCA, the to 100 genes with the largest absolute values of ***u*** and the top 10 miRNAs with the largest absolute values of ***v*** are considered as a module, denoted as pcModule.

We then analyzed the biological functionality for pcModule from multiple biological perspectives (S26 Table). We first found that the miRNAs and genes within the module are not strongly related to most cancers (S8A Fig). In the pcModule, there are five cancer miRNAs including hsa-miR-324-5p, hsa-miR-484, hsa-miR-186-5p, hsa-miR-590-5p, and hsa-miR-423-3p and the cancer miRNA set enrichment analysis shows the result is not significant. In addition, there are 15 cancer genes within the module, including IL6ST, GADD45GIP1, NAA10, AFF4, BRMS1, NDUFA13, CHD9, RUVBL2, LNPEP, STAG1, ETV3, ARHGEF12, ATRX, PPTC7, PPP1R14B. Cancer gene set enrichment analysis shows the result is also not significant. More results of pcModule are shown in S26 Table. We also compared the difference of modularity scores between the pcModule and TSCCA modules, and we found that the modularity value of pcModule is significantly smaller than that of all TSCCA modules (Wilcoxon rank-sum test *P* < 0.05, S8B Fig).

Table S26. Results of pcModule. “#cancer miR”, “#cancer gene”, “#gene edge” and “#miR-gene edge” denote the number of cancer miRNAs, cancer genes, gene edges and miRNA-gene edges. “*” stands for *p*-value < 0.05.

|  | #cancer  miR | #cancer  gene | #gene  edge | #miR-gene  edge | modularity |
| --- | --- | --- | --- | --- | --- |
| pcModule | 5 | 15 | 63* | 32 | 0.15 |

**23** **Results of gene-gene and miRNA-gene interaction set enrichment**

We developed a statistical method based on permutation test, which is used to calculate gene-gene and miRNA-gene interaction set enrichment by permuting genes/miRNAs conditioned on their degree in the networks (see sections 15 and 16 in S1 Text for more details). In S27 Table, we show the results of gene-gene interaction set enrichment analysis results of each identified module by TSCCA. Similarly, we also show the results of miRNA-gene interaction set enrichment analysis results of each identified module by TSCCA (S28 Table).

**Table S27.** Gene-gene interaction set enrichment for the identified modules by TSCCA on the TCGA dataset.

| Module | #gene  edge | sum of gene degree | *p*-value by geometric test | *p*-value by permutation test |
| --- | --- | --- | --- | --- |
| 1 | 505 | 7835 | 0.00E+00 | 0.001 |
| 2 | 95 | 5144 | 1.13E-17 | 0.001 |
| 3 | 57 | 4605 | 2.47E-04 | 0.001 |
| 4 | 253 | 7738 | 1.03E-128 | 0.001 |
| 5 | 57 | 4672 | 2.47E-04 | 0.001 |
| 6 | 72 | 6906 | 1.31E-08 | 0.010 |
| 7 | 21 | 3721 | 9.94E-01 | 0.045 |
| 8 | 55 | 4608 | 7.03E-04 | 0.001 |
| 9 | 27 | 4363 | 9.16E-01 | 0.081 |
| 10 | 536 | 8688 | 0.00E+00 | 0.001 |
| 11 | 172 | 8282 | 1.88E-63 | 0.001 |
| 12 | 88 | 8939 | 1.25E-14 | 0.440 |
| 13 | 34 | 4175 | 5.51E-01 | 0.001 |
| 14 | 111 | 6268 | 1.88E-25 | 0.001 |
| 15 | 64 | 6008 | 3.80E-06 | 0.004 |
| 16 | 28 | 3524 | 8.84E-01 | 0.008 |
| 17 | 32 | 4389 | 6.83E-01 | 0.005 |
| 18 | 44 | 4302 | 6.41E-02 | 0.001 |
| 19 | 263 | 10223 | 1.12E-137 | 0.001 |
| 20 | 60 | 4532 | 4.53E-05 | 0.001 |
| 21 | 20 | 3441 | 9.97E-01 | 0.012 |
| 22 | 79 | 7991 | 4.49E-11 | 0.140 |
| 23 | 54 | 6407 | 1.16E-03 | 0.044 |
| 24 | 517 | 7827 | 0.00E+00 | 0.001 |
| 25 | 36 | 4679 | 4.15E-01 | 0.004 |
| 26 | 82 | 4869 | 3.26E-12 | 0.001 |
| 27 | 40 | 4868 | 1.90E-01 | 0.011 |
| 28 | 470 | 8471 | 0.00E+00 | 0.001 |
| 29 | 77 | 7808 | 2.43E-10 | 0.041 |
| 30 | 207 | 7845 | 6.50E-90 | 0.001 |
| 31 | 42 | 4373 | 1.15E-01 | 0.001 |
| 32 | 33 | 4370 | 6.18E-01 | 0.012 |
| 33 | 28 | 4632 | 8.84E-01 | 0.255 |
| 34 | 22 | 4293 | 9.90E-01 | 0.401 |
| 35 | 61 | 3748 | 2.49E-05 | 0.001 |
| 36 | 38 | 4624 | 2.91E-01 | 0.004 |
| 37 | 29 | 3999 | 8.44E-01 | 0.010 |
| 38 | 48 | 3926 | 1.60E-02 | 0.001 |
| 39 | 54 | 4586 | 1.16E-03 | 0.001 |
| 40 | 156 | 7338 | 2.20E-52 | 0.001 |
| 41 | 142 | 4267 | 2.69E-43 | 0.001 |
| 42 | 58 | 4965 | 1.42E-04 | 0.001 |
| 43 | 56 | 6146 | 4.20E-04 | 0.037 |
| 44 | 34 | 4509 | 5.51E-01 | 0.011 |
| 45 | 90 | 6367 | 1.78E-15 | 0.001 |
| 46 | 20 | 3682 | 9.97E-01 | 0.195 |
| 47 | 85 | 5536 | 2.12E-13 | 0.001 |
| 48 | 48 | 5003 | 1.60E-02 | 0.003 |
| 49 | 68 | 5283 | 2.50E-07 | 0.001 |
| 50 | 37 | 4779 | 3.51E-01 | 0.003 |

**Table S28.** miRNA-gene interaction set enrichment for the identified modules by TSCCA on the TCGA dataset.

| Module | #miR-gene edge | sum of miR degree | *p*-value by geometric test | *p*-value by permutation test |
| --- | --- | --- | --- | --- |
| 1 | 33 | 2363 | 2.55E-06 | 0.053 |
| 2 | 13 | 1084 | 5.56E-01 | 0.346 |
| 3 | 12 | 2465 | 6.65E-01 | 0.782 |
| 4 | 57 | 2363 | 1.46E-19 | 0.001 |
| 5 | 25 | 1909 | 2.18E-03 | 0.016 |
| 6 | 63 | 3711 | 1.13E-23 | 0.001 |
| 7 | 10 | 1031 | 8.47E-01 | 0.594 |
| 8 | 16 | 721 | 2.50E-01 | 0.193 |
| 9 | 42 | 2964 | 1.33E-10 | 0.001 |
| 10 | 44 | 2533 | 1.12E-11 | 0.003 |
| 11 | 36 | 2490 | 1.21E-07 | 0.019 |
| 12 | 65 | 3711 | 4.19E-25 | 0.001 |
| 13 | 12 | 1407 | 6.65E-01 | 0.458 |
| 14 | 33 | 1954 | 2.55E-06 | 0.006 |
| 15 | 10 | 721 | 8.47E-01 | 0.640 |
| 16 | 18 | 1295 | 1.17E-01 | 0.091 |
| 17 | 30 | 3714 | 4.10E-05 | 0.066 |
| 18 | 5 | 1031 | 9.97E-01 | 0.926 |
| 19 | 38 | 3081 | 1.39E-08 | 0.026 |
| 20 | 17 | 1750 | 1.75E-01 | 0.381 |
| 21 | 7 | 1122 | 9.77E-01 | 0.798 |
| 22 | 16 | 1018 | 2.50E-01 | 0.313 |
| 23 | 73 | 3701 | 4.25E-31 | 0.001 |
| 24 | 13 | 1192 | 5.56E-01 | 0.500 |
| 25 | 14 | 1909 | 4.45E-01 | 0.380 |
| 26 | 11 | 1073 | 7.64E-01 | 0.505 |
| 27 | 4 | 1295 | 9.99E-01 | 0.964 |
| 28 | 23 | 912 | 8.33E-03 | 0.095 |
| 29 | 55 | 3050 | 2.97E-18 | 0.001 |
| 30 | 56 | 2457 | 6.65E-19 | 0.001 |
| 31 | 18 | 1051 | 1.17E-01 | 0.105 |
| 32 | 25 | 1730 | 2.18E-03 | 0.070 |
| 33 | 8 | 1302 | 9.52E-01 | 0.723 |
| 34 | 9 | 918 | 9.09E-01 | 0.684 |
| 35 | 8 | 679 | 9.52E-01 | 0.710 |
| 36 | 12 | 678 | 6.65E-01 | 0.494 |
| 37 | 15 | 2464 | 3.41E-01 | 0.487 |
| 38 | 18 | 2097 | 1.17E-01 | 0.202 |
| 39 | 13 | 1002 | 5.56E-01 | 0.347 |
| 40 | 16 | 1228 | 2.50E-01 | 0.514 |
| 41 | 5 | 503 | 9.97E-01 | 0.938 |
| 42 | 2 | 957 | 1.00E+00 | 0.997 |
| 43 | 31 | 2261 | 1.68E-05 | 0.017 |
| 44 | 11 | 1055 | 7.64E-01 | 0.544 |
| 45 | 8 | 900 | 9.52E-01 | 0.788 |
| 46 | 43 | 3711 | 3.91E-11 | 0.002 |
| 47 | 15 | 750 | 3.41E-01 | 0.338 |
| 48 | 20 | 2128 | 4.62E-02 | 0.199 |
| 49 | 5 | 475 | 9.97E-01 | 0.940 |
| 50 | 2 | 669 | 1.00E+00 | 0.992 |

**24 What are specific and shared modules?**

So-called the shared miRNA-gene co-expressed module means that the expression of the genes and miRNAs in this module are strongly are strongly correlated in expression on most cancers, and it is a relative definition in our study. For example, the miRNAs and genes within modules 1, 4, and 10 have a strong positive correlation on all the cancer types (S9A Fig). The miRNAs and genes within modules 5, 8, and 9 have a strong negative correlation on all the selected cancer types (S9B Fig). We call these modules shared modules.

The so-called cancer-specific module means that the correlation pattern between the genes and miRNAs in the specific cancer is different from the correlation patterns between the genes and miRNAs in other cancer types. For example, module 31 is a TGCT-cancer-specific miRNA-gene co-expressed module (S9C Fig).

**25 Statistical analysis and software**

Most of statistical experiments were executed on R Studio, and also some were on Cytoscape software.

**Reference**

1. Xu, Yangyang, and Wotao Yin (2017). A globally convergent algorithm for nonconvex optimization based on block coordinate update. *Journal of Scientific Computing* **72**,700-734.
2. Allen, G. (2012) Sparse higher-order principal components analysis. In *AISTATS*, pp.27-36.
3. Zhang S, Li Q, Liu J, *et al.* (2011) A novel computational framework for simultaneous integration of multiple types of genomic data to identify microRNA-gene regulatory modules. *Bioinformatics*, **27**, i401-i409.
4. Li, Y., Liang, C., Wong, K.-C., Luo, J., and Zhang, Z. (2014) Mirsynergy: detecting synergistic miRNA regulatory modules by overlapping neighbourhood expansion. *Bioinformatics*, **30**, 2627–2635.
5. Terrile M, Bray I M, *et al.* Discovery and visualization of miRNA–mRNA functional modules within integrated data using bicluster analysis. *Nucleic Acids Res.*, 2013, **42**, e17.
6. Benjamini, Y., Hochberg, Y. (1995) Controlling the false discovery rate: a practical and powerful approach to multiple testing. *J. R. STAT. SOC. B.*, **57**: 289-300.
7. Bentham R B, Bryson K, Szabadkai G. MCbiclust (2017) A novel algorithm to discover large-scale functionally related gene sets from massive transcriptomics data collections. *Nucleic Acids Res.*, **45**: 8712-8730.
8. Prelić, Amela, *et al.* (2016) A systematic comparison and evaluation of biclustering methods for gene expression data. *Bioinformatics*, **22**, 1122-1129.
9. Patrikainen, *et al.* (2006) Comparing subspace clusterings. *IEEE Trans. Knowl. Data Eng.*, **18**, 902-916.
10. G. Allen. (2012) Sparse higher-order principal components analysis. *Artificial Intelligence and Statistics*, pp. 27–36.
11. R. Henriques and S. C. Madeira. (2018) Triclustering algorithms for three-dimensional data analysis: a comprehensive survey. *ACM Computing Surveys*, **51**: 1–43.

**26 Supplementary Figures and Tables**

**S1 Fig. Convergence analysis of 50 modules identified by TSCCA on the TCGA dataset across 33 cancer types.**

**S2 Fig. Heatmap of cancer-miRNA-gene modules identified by TSCCA in the TCGA dataset.** Each subfigure corresponds to an identified module and a random module. In each subfigure, the top half corresponds to the identified module (row corresponds to gene, column corresponds to miRNA) and the lower part of is a random module for comparison. (A) Showing the Heatmap of modules 1 to 5. (B) Showing the Heatmap of modules 6 to 10. (C) Showing the Heatmap of modules 11 to 15. (D) Showing the Heatmap of modules 16 to 20. (E) Showing the Heatmap of modules 21 to 25. (F) Showing the Heatmap of modules 26 to 30. (G) Showing the Heatmap of modules 31 to 35. (H) Showing the Heatmap of modules 36 to 40. (I) Showing the Heatmap of modules 41 to 45. (J) Showing the Heatmap of modules 46 to 50.

**S3 Fig.** **Characteristics of modules in different cancers.** (A) Heatmap showing the output matrix W of Algorithm 2, when it was applied to the TCGA data. Each column corresponds to a module and each row corresponds to a cancer type and $W_{ij}$ reflects the co-expressed intensity between the genes and the miRNAs within the module $j$ on the cancer $i$. A hierarchical clustering method was used to cluster the rows (cancer types) into four clusters. (B) Scatter plot for elements of the W matrix. There are three negative elements/pairs in W, where (Module 31, TGCT) is -0.145, (Module 49,TGCT) is -0.23 and (Module 49, UCS) is -0.138 and (C) Their heatmaps shown in the blue frame.

**S4 Fig. Application of the TSCCA onto the subset of TCGA cancer data from the cluster 3 in Fig 4 and extract 50 modules.** We first extracted a subset of cancers (A) and then re-used TSCCA to extract 50 modules on the subset of the previous data, and we found some new modules with significant modularity scores (B). Finally, we show the heatmap of the corresponding W matrix (C).

**S5 Fig.** **miRNA-gene regulatory network analysis of modules.** (A) For each identified module, a produce is developed to identify a largest connected subgraph, i.e., a three-layer miRNA-gene regulatory network, where the miRNA-gene interactions are from miRTarBase network and the gene-gene interactions are from the gene interaction network, and miRNAs regulate genes and these genes regulate the other genes with three-layer network. (B) A miRNA-gene network contains 3619 experimentally verified miRNA-gene interactions from miRTarBase network via combing all genes and miRNAs of modules identified by TSCCA (Hypergeometric test *P* = 3.5e-43).

**S6 Fig. Heatmap of cancer-miRNA-gene modules identified by different methods in the TCGA dataset.** The top half of each heatmap corresponds to the module 1 (row corresponds to gene, column corresponds to miRNA) and the lower part is a random module for comparison.

**S7 Fig. Comparison of different methods on the TCGA data in terms of Modularity score (A) and time (B).** We also compared the running time of different methods on a personal laptop. Box-plots show results in terms of modularity scores and running time of algorithm based on 50 different initializations of each method.

**S8 Fig. Results of pcModule.** (A) Heatmap of pcModule. The top half of each heatmap corresponds to the module 1 (row corresponds to gene, column corresponds to miRNA) and the lower part is a random module for comparison. (B) Comparison of modularity scores of pcModule and TSCCA modules.

**S9 Fig. Heatmap of some modules identified by TSCCA in the TCGA dataset.** (A) Heatmap of modules 1, 4 and 10. (B) Heatmap of modules 5, 8 and 9. (C) Heatmap of cancer-miRNA-gene module 31 identified by TSCCA in the TCGA dataset. Module 31 is a TGCT-cancer-specific miRNA-gene co-expressed module.

**S1 Table. The list of 7889 significant different expression genes with BH adjusted *P* < 0.05 in at least 15 cancer types.**

**S2 Table. Summary of the TCGA data.**

**S3 Table. Objective function values (Singular values) of modules identified by TSCCA.**

**S4 Table. Cancer types and weights of modules identified by TSCCA.**

**S5 Table. MiRNA members and weights of modules identified by TSCCA.**

**S6 Table. Gene members and weights of modules identified by TSCCA.**

**S7 Table. Summary of modules concerning gene names, miRNA names and cancer type names.**

**S8 Table. Significant overlap between two miRNA-gene-cancer modules/subtensors in a binary form.**

**S9 Table. Modularity values for different cancer types.**

**S10 Table. Enrichment analysis of modules in terms of cancer miRNAs, cancer genes, PPIs and miRNA-gene interactions.**

**S11 Table. Number of significant terms.**

**S12 Table. Significant GOBP terms.**

**S13 Table. Significant KEGG terms.**

**S14 Table. Significant Reactome terms.**

**S15 Table. Module miRNAs are cooperative within miRNA families.**

**S16 Table. Largest connected subnetwork (LCS) of modules where each edge is from verified miRNA-gene and gene-gene interactions.**

**S17 Table. Prognostic miRNA-gene module biomarkers in multiple cancer types.**

**S18 Table. Prognostic miRNA biomarkers in multiple cancer types.**

**S19 Table. Biological functional analysis of selected cancer-miRNA-gene modules.**

**S20 Table. Comparison (in terms of CE ± std) on the simulated data.**

**S21 Table. Comparison (in terms of Recovery ± std) on the simulated data.**

**S22 Table. Comparison (in terms of CE ± std) on the simulated data with different variances.**

**S23 Table. Comparison of the (in terms of Recovery ± std) on the simulated data with different variables.**

**S24 Table. Performance comparison of TSCCA and SCCA, where we applied SCCA to identify 50 modules on each cancer data set.**

**S25 Table. Performance comparison of TSCCA and the triclustering methods.**

**S26 Table. Results of pcModule.**

**S27 Table. Gene-gene interaction set enrichment for the identified modules by TSCCA on the TCGA data.**

**S28 Table. miRNA-gene interaction set enrichment for the identified modules by TSCCA on the TCGA data.**
