# Supplementary figures and images for: TSCCA: A tensor sparse CCA method for detecting microRNA-gene patterns from multiple cancers
Source: PLoS Comput Biol. 2021 Jun 1;17(6):e1009044. doi: 10.1371/journal.pcbi.1009044 (PMC8195367; doi:10.1371/journal.pcbi.1009044)

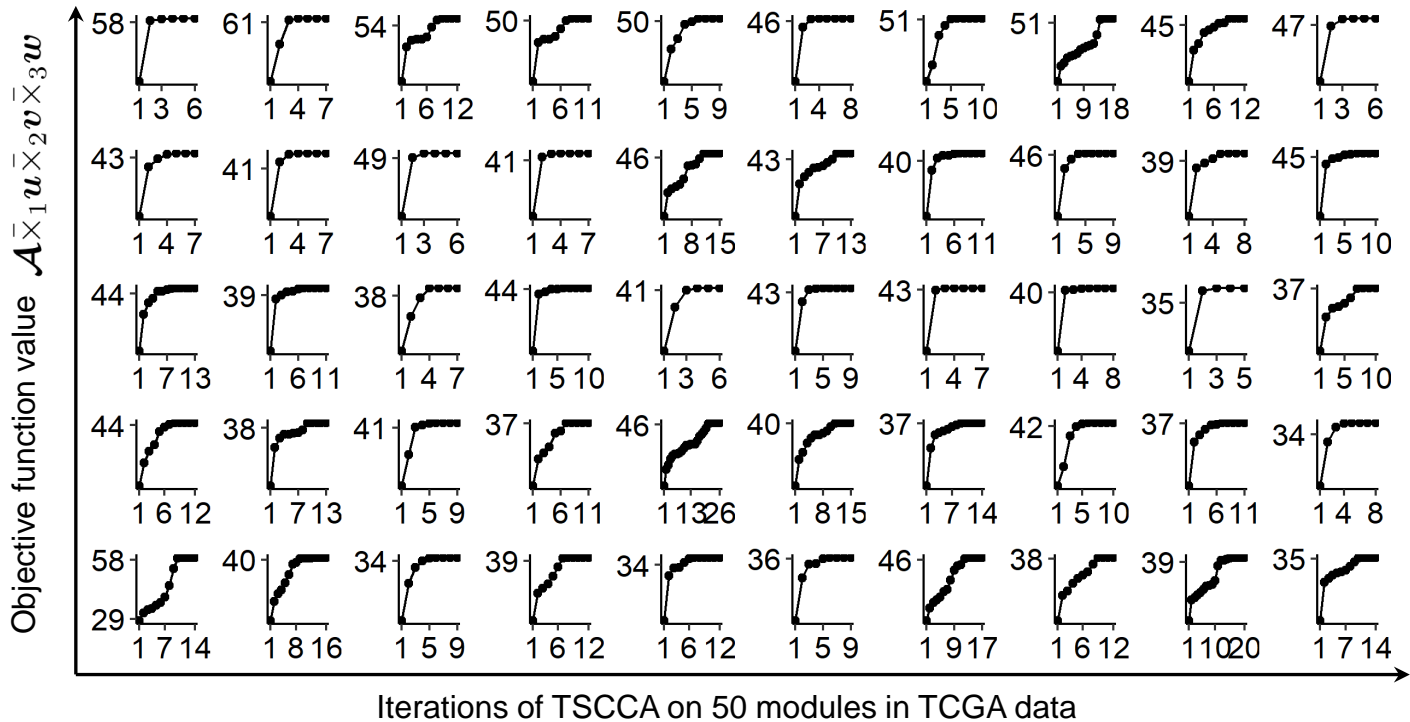

Supplement: S1 Fig — (PDF) [file pcbi.1009044.s002.pdf]

(A)

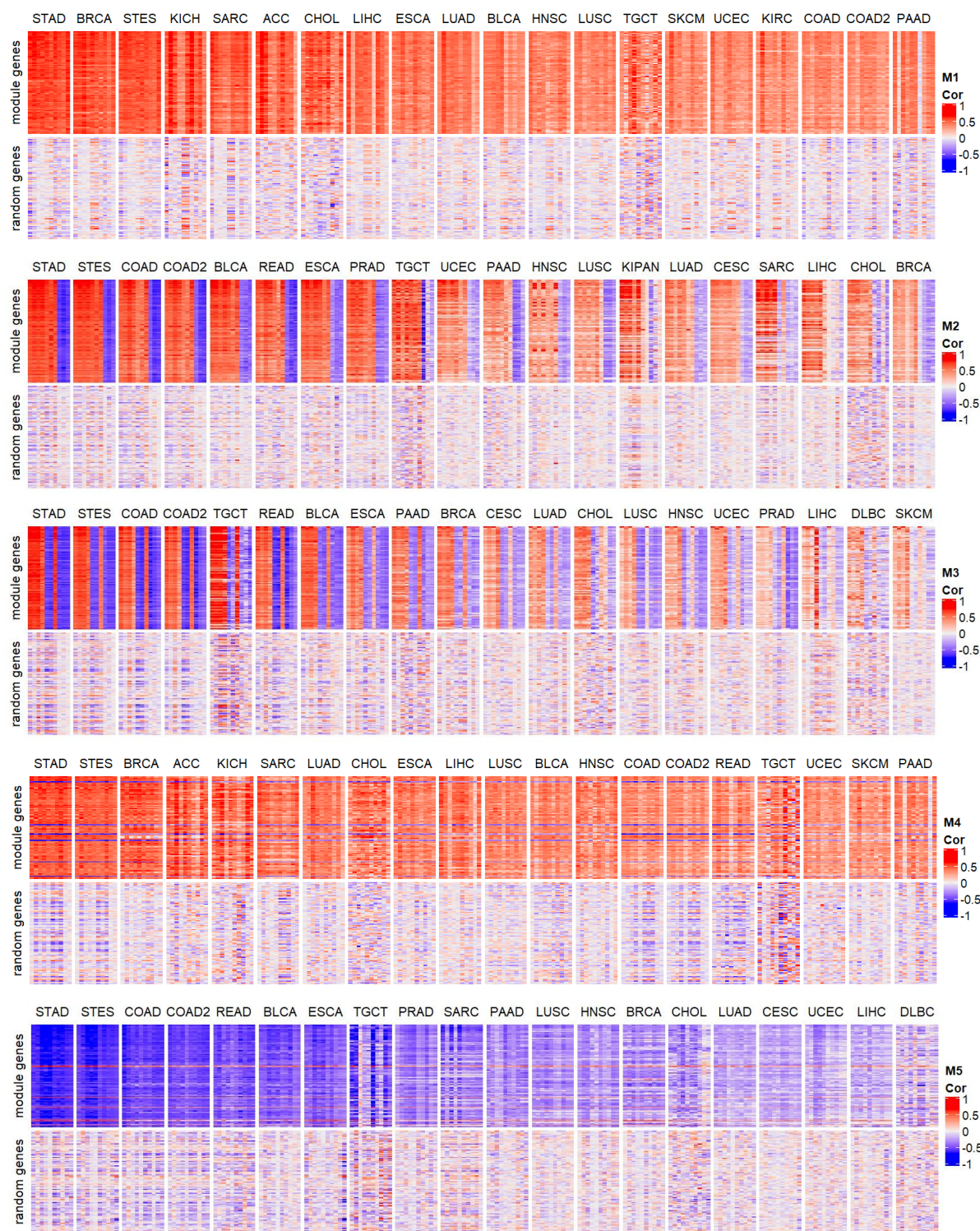

(B)

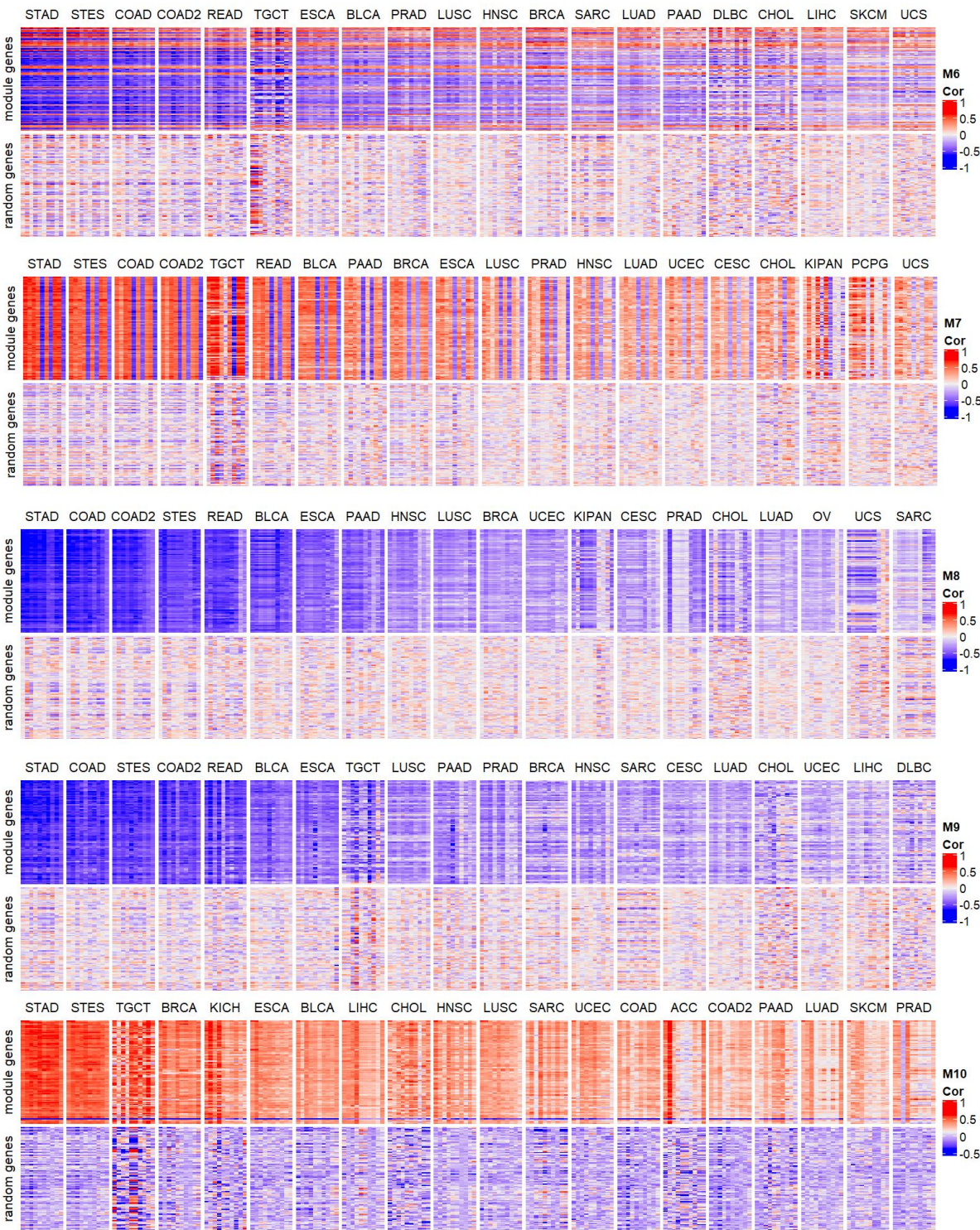

(C)

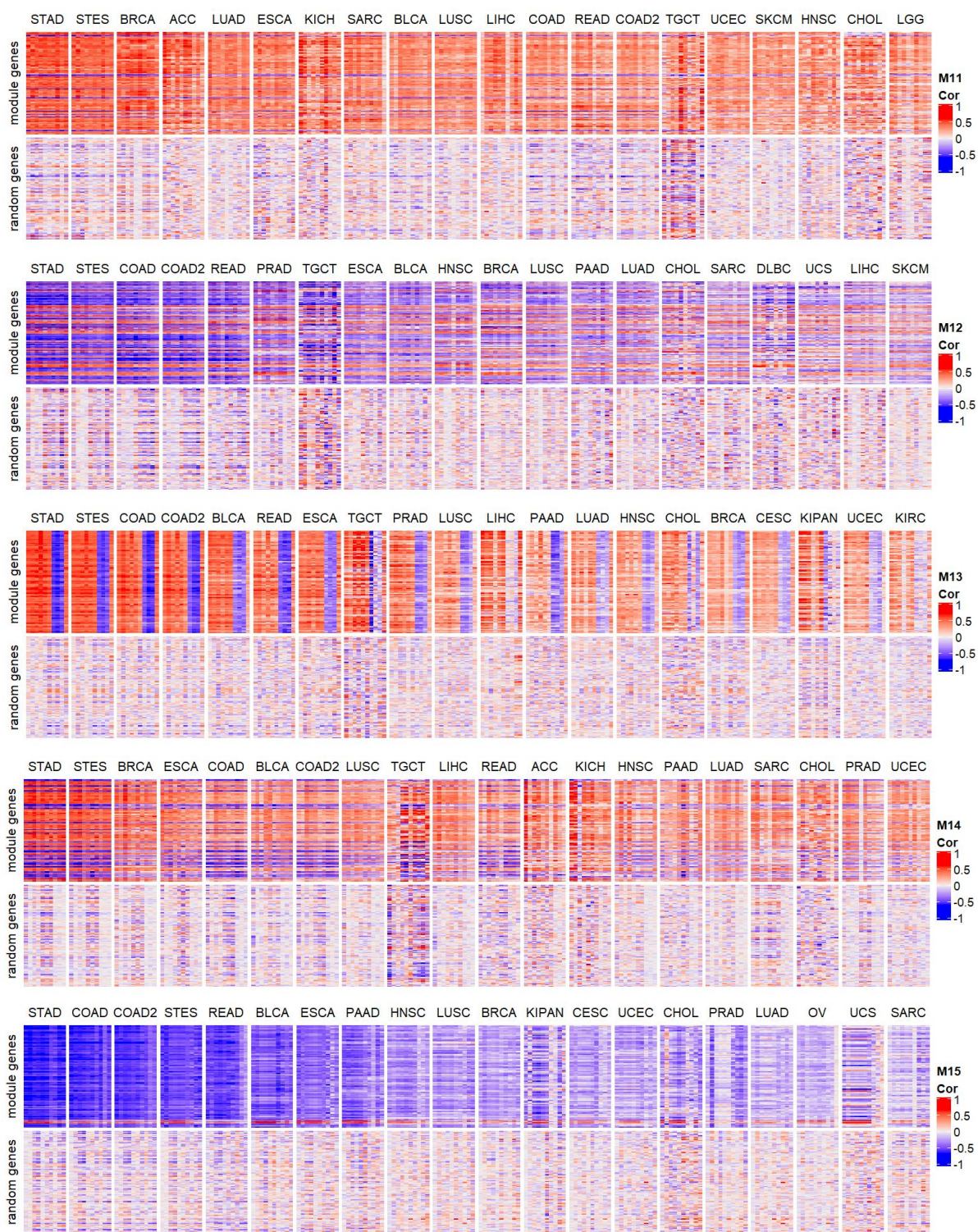

(D)

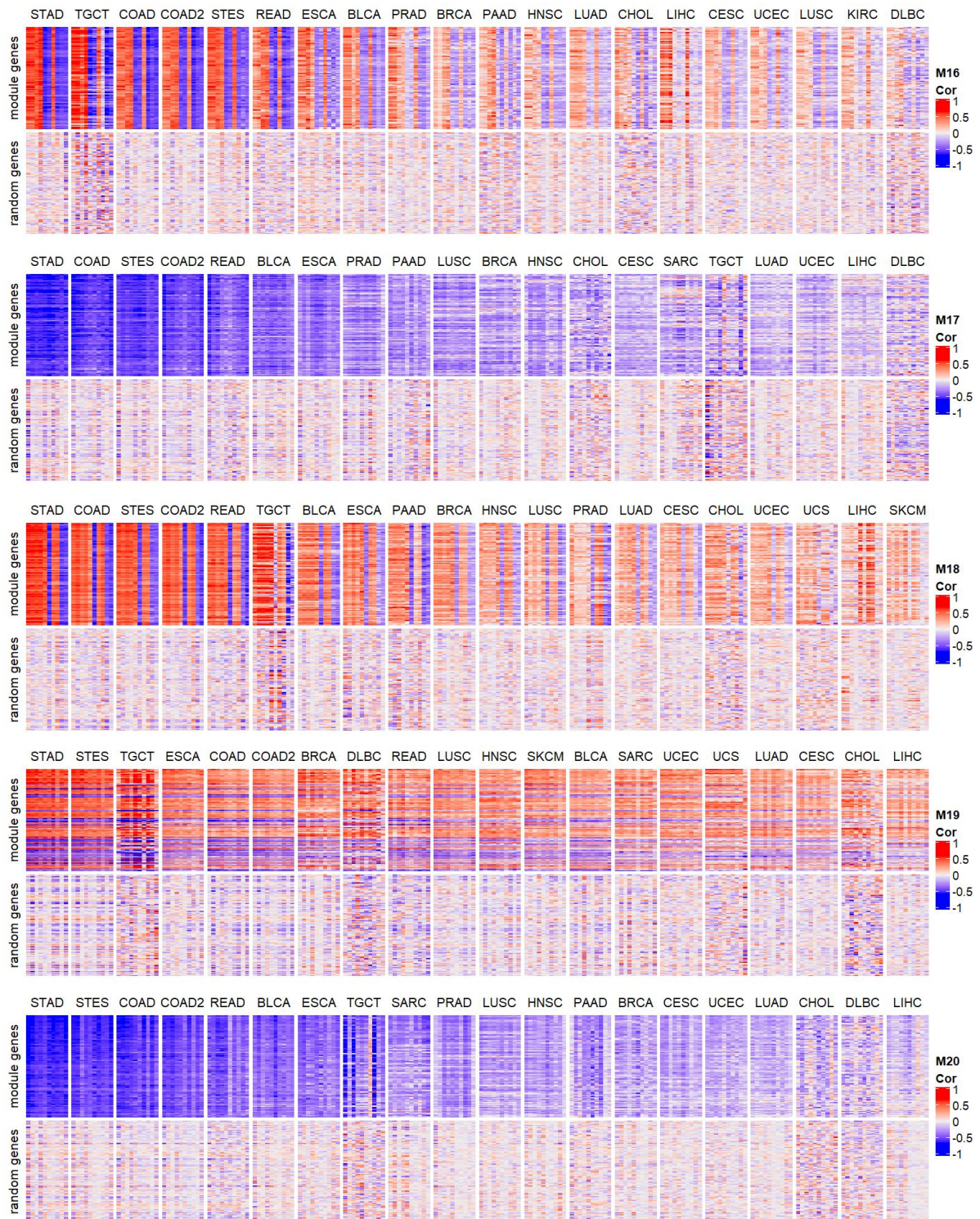

(E)

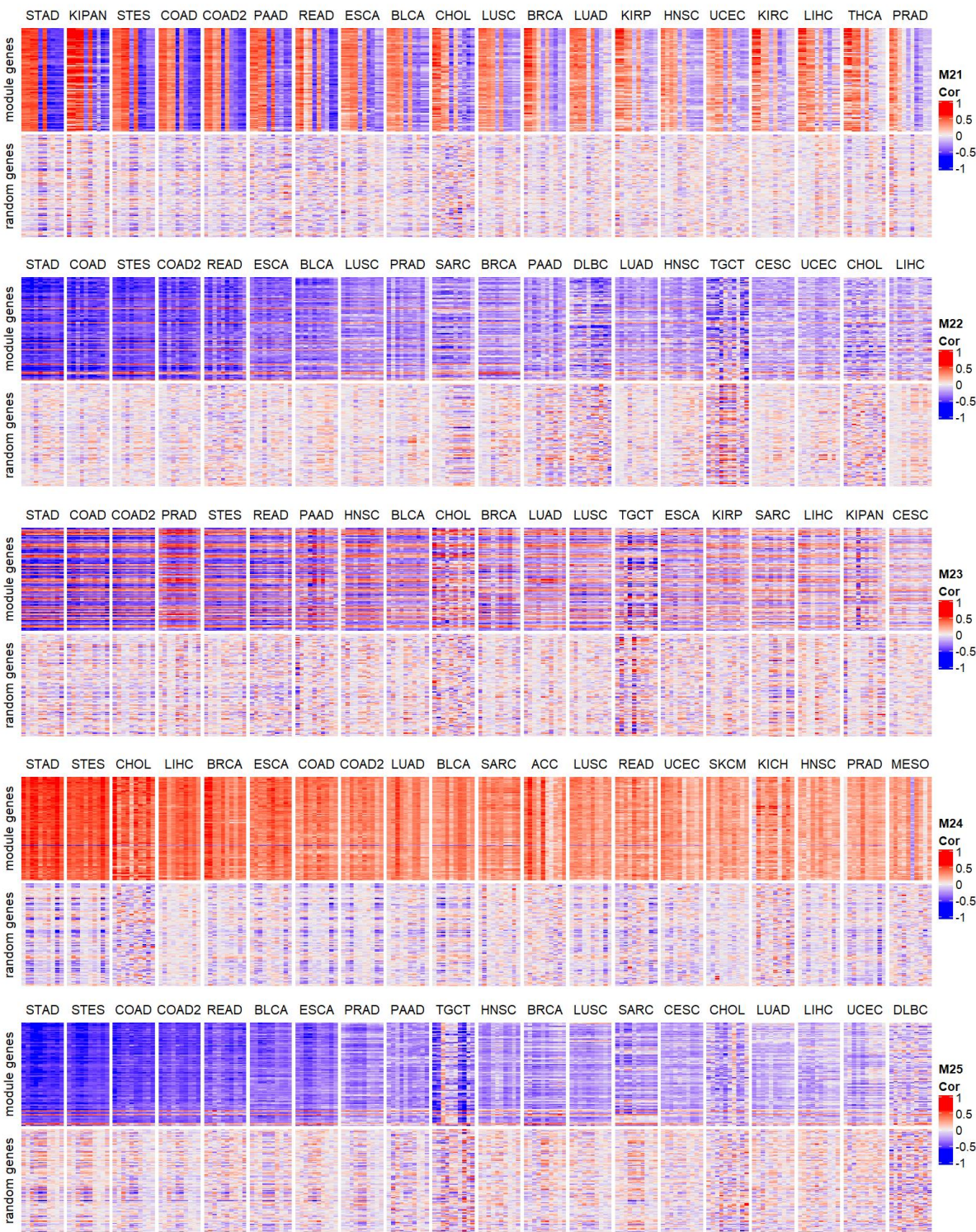

(F)

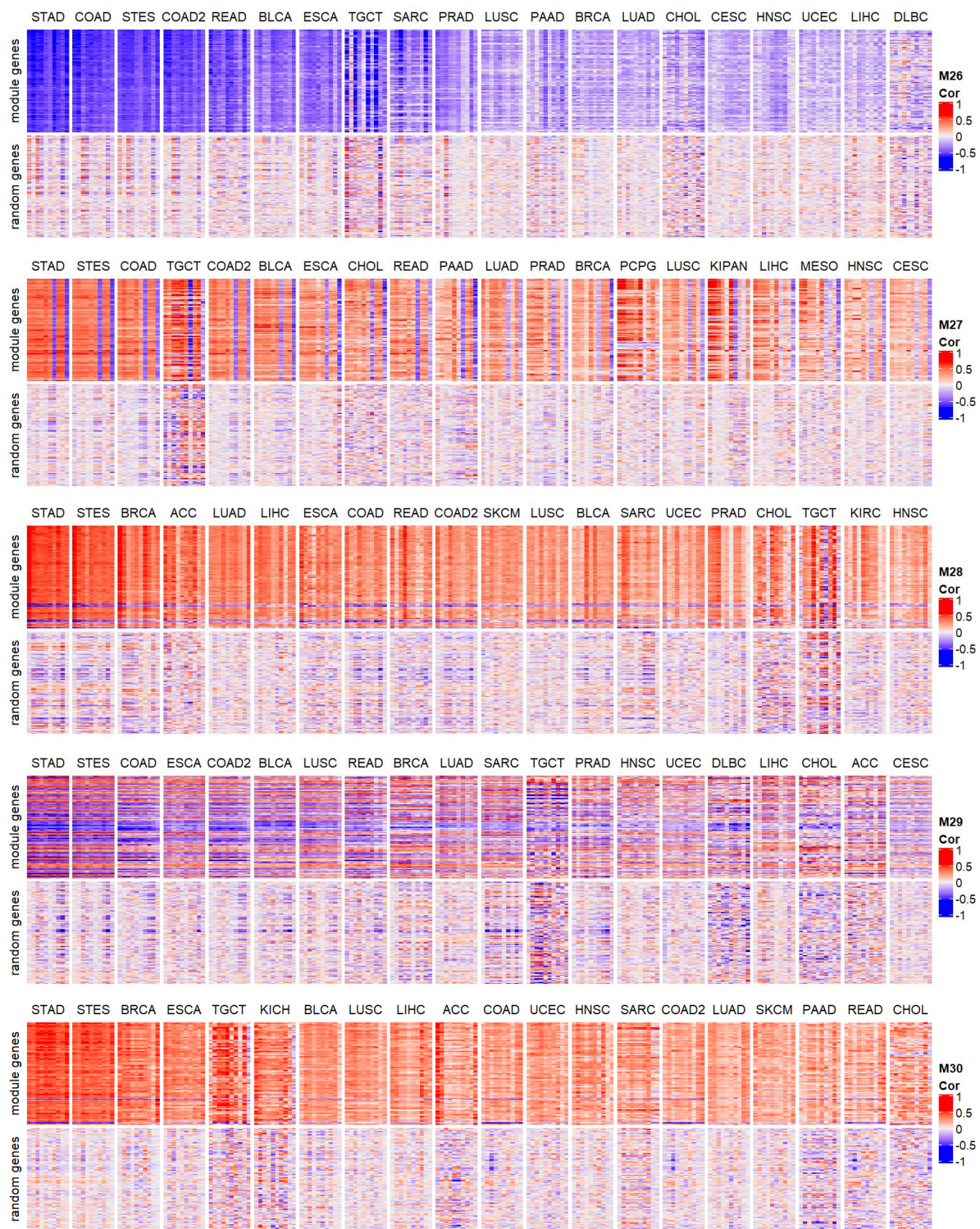

(G)

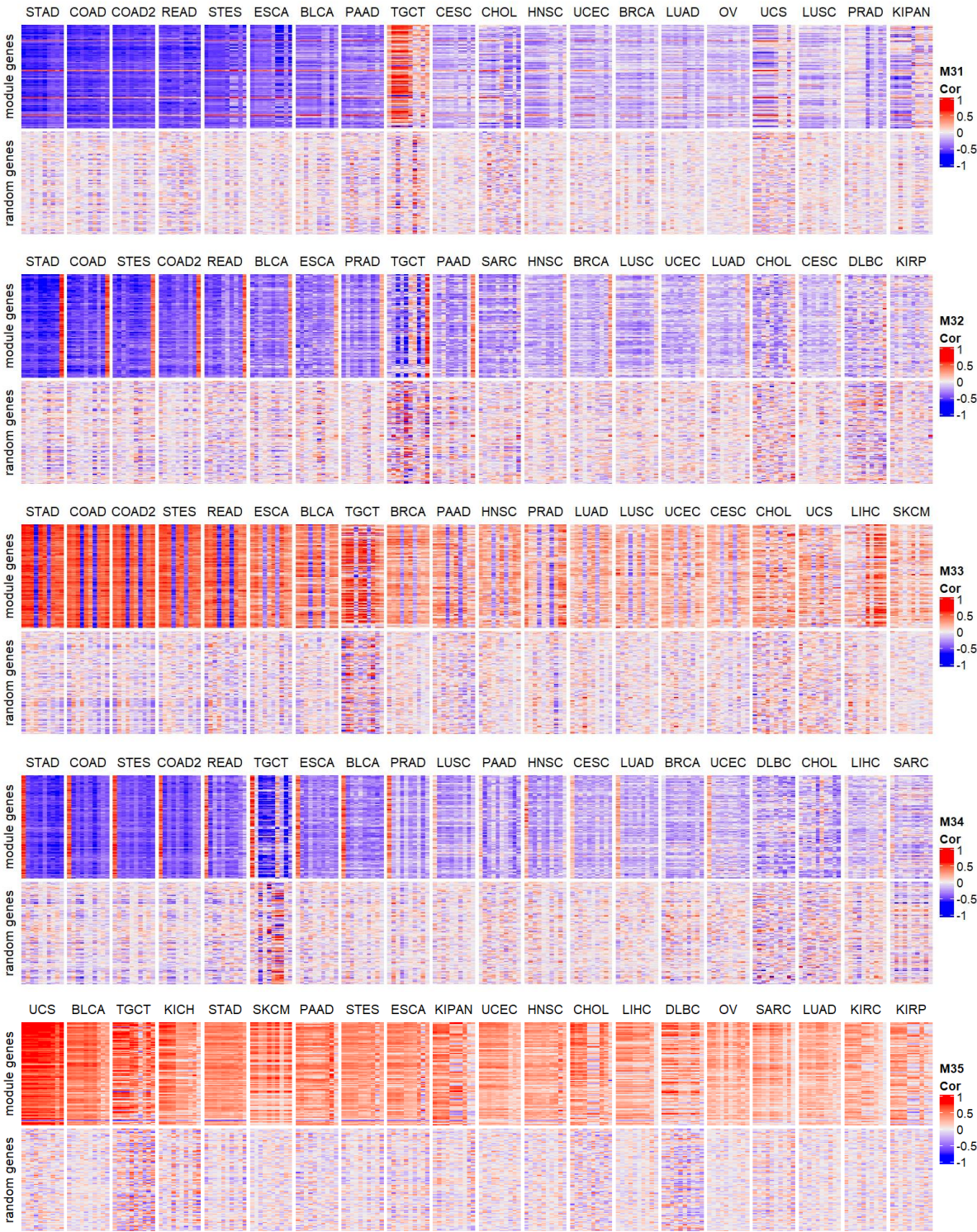

(H)

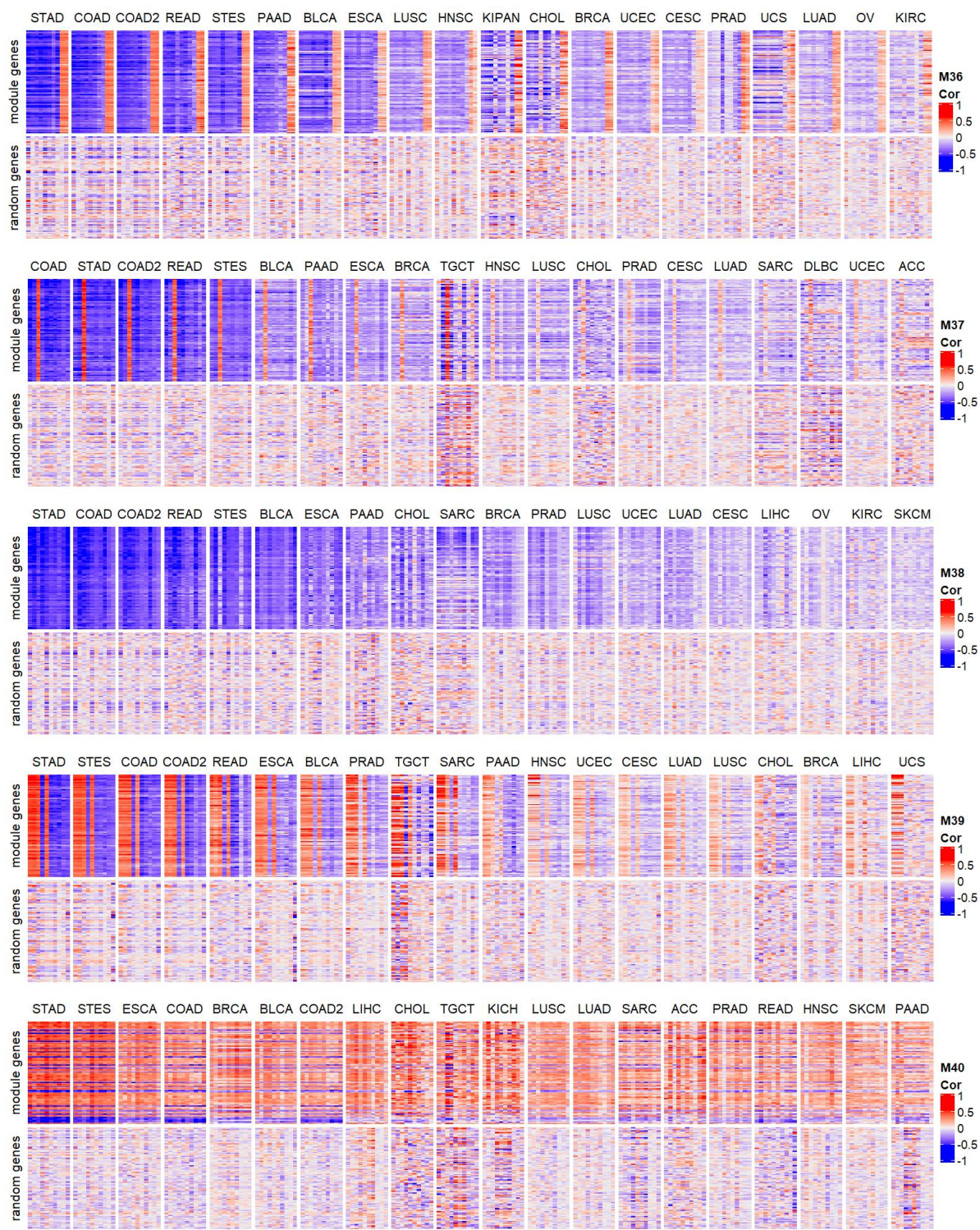

(I)

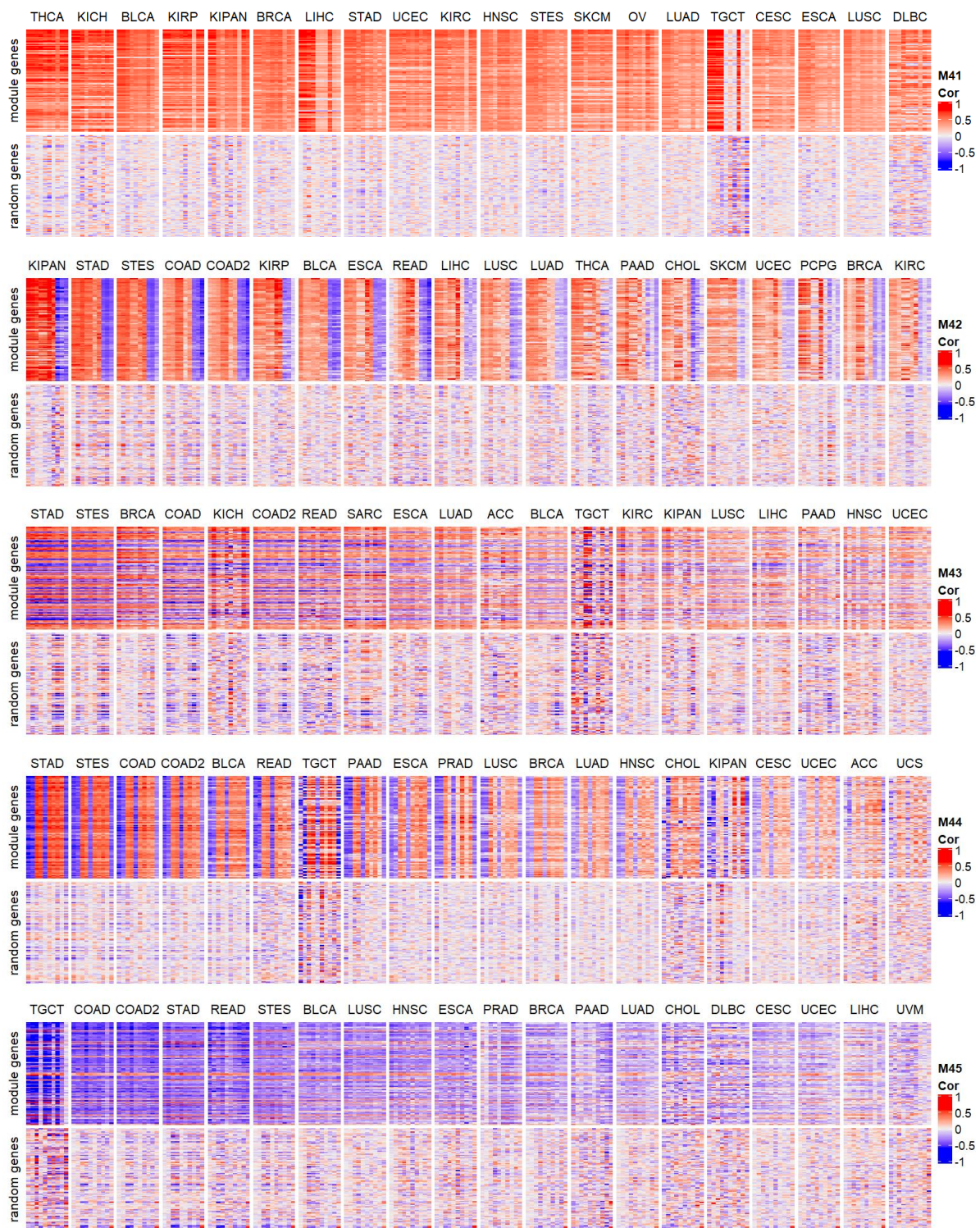

(J)

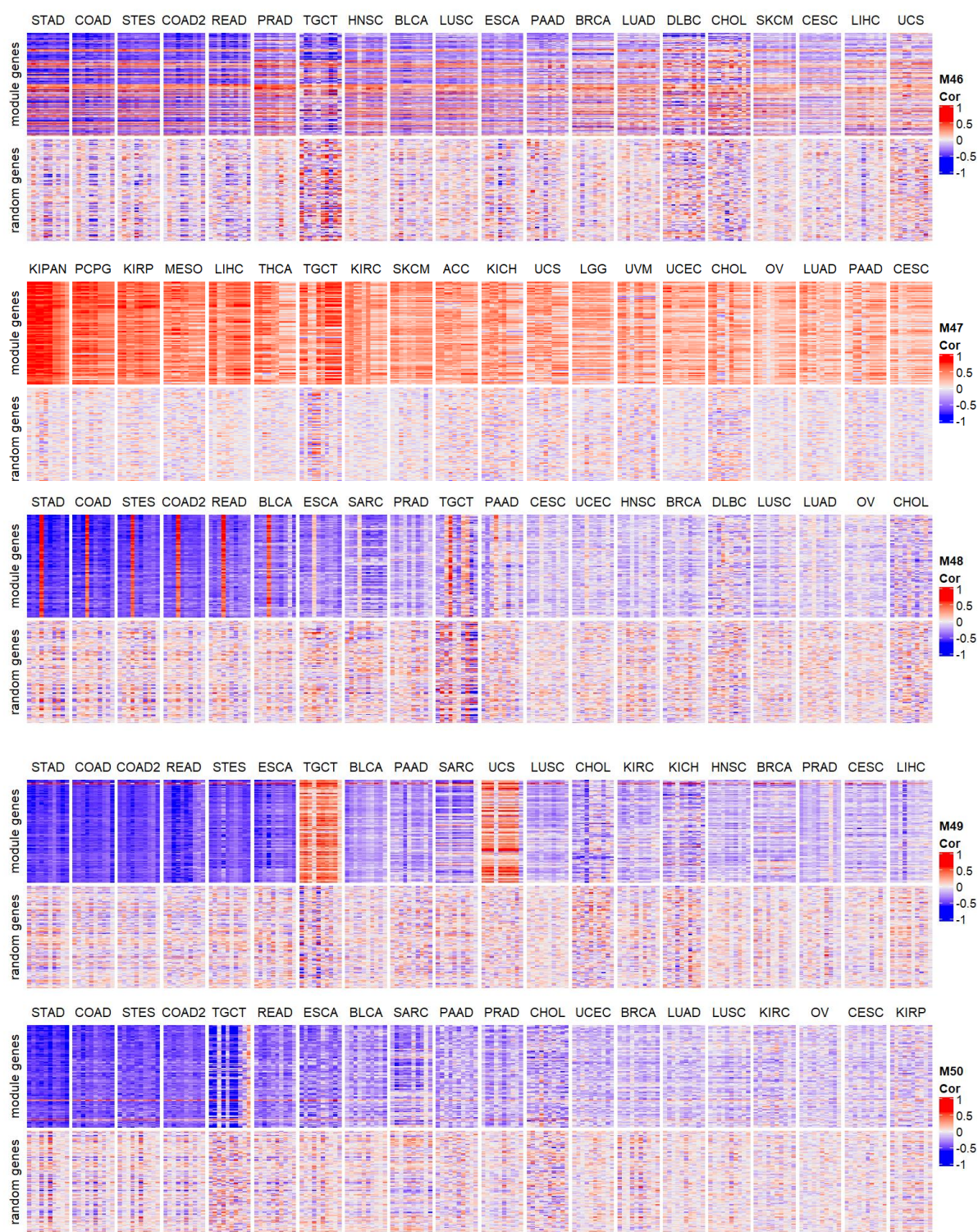

Supplement: S2 Fig — Each subfigure corresponds to an identified module and a random module. In each subfigure, the top half corresponds to the identified module (row corresponds to gene, column corresponds to miRNA) and the lower part of is a random module for comparison. (A) Showing the Heatmap of modules 1 to 5. (B) Showing the Heatmap of modules 6 to 10. (C) Showing the Heatmap of modules 11 to 15. (D) Showing the Heatmap of modules 16 to 20. (E) Showing the Heatmap of modules 21 to 25. (F) Showing the Heatmap of modules 26 to 30. (G) Showing the Heatmap of modules 31 to 35. (H) Showing the Heatmap of modules 36 to 40. (I) Showing the Heatmap of modules 41 to 45. (J) Showing the Heatmap of modules 46 to 50. (PDF) [file pcbi.1009044.s003.pdf]

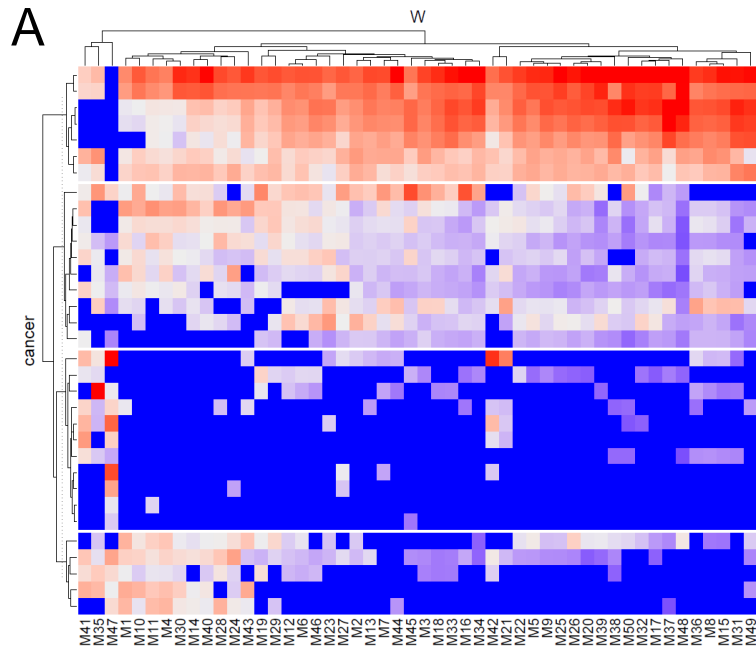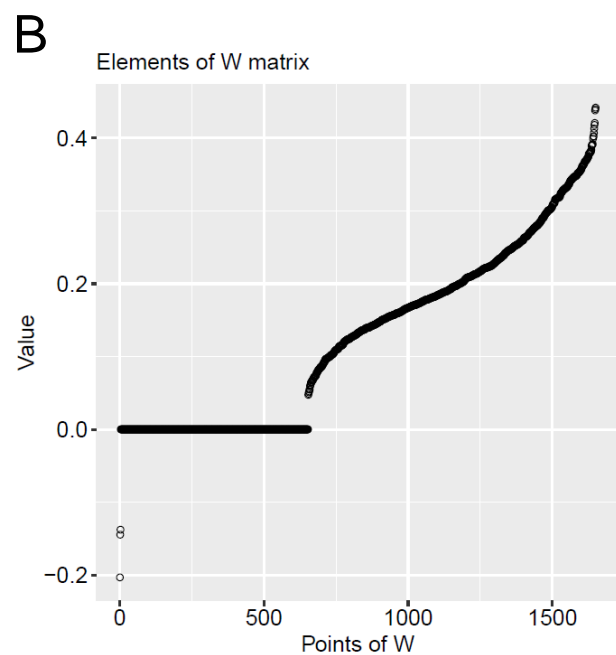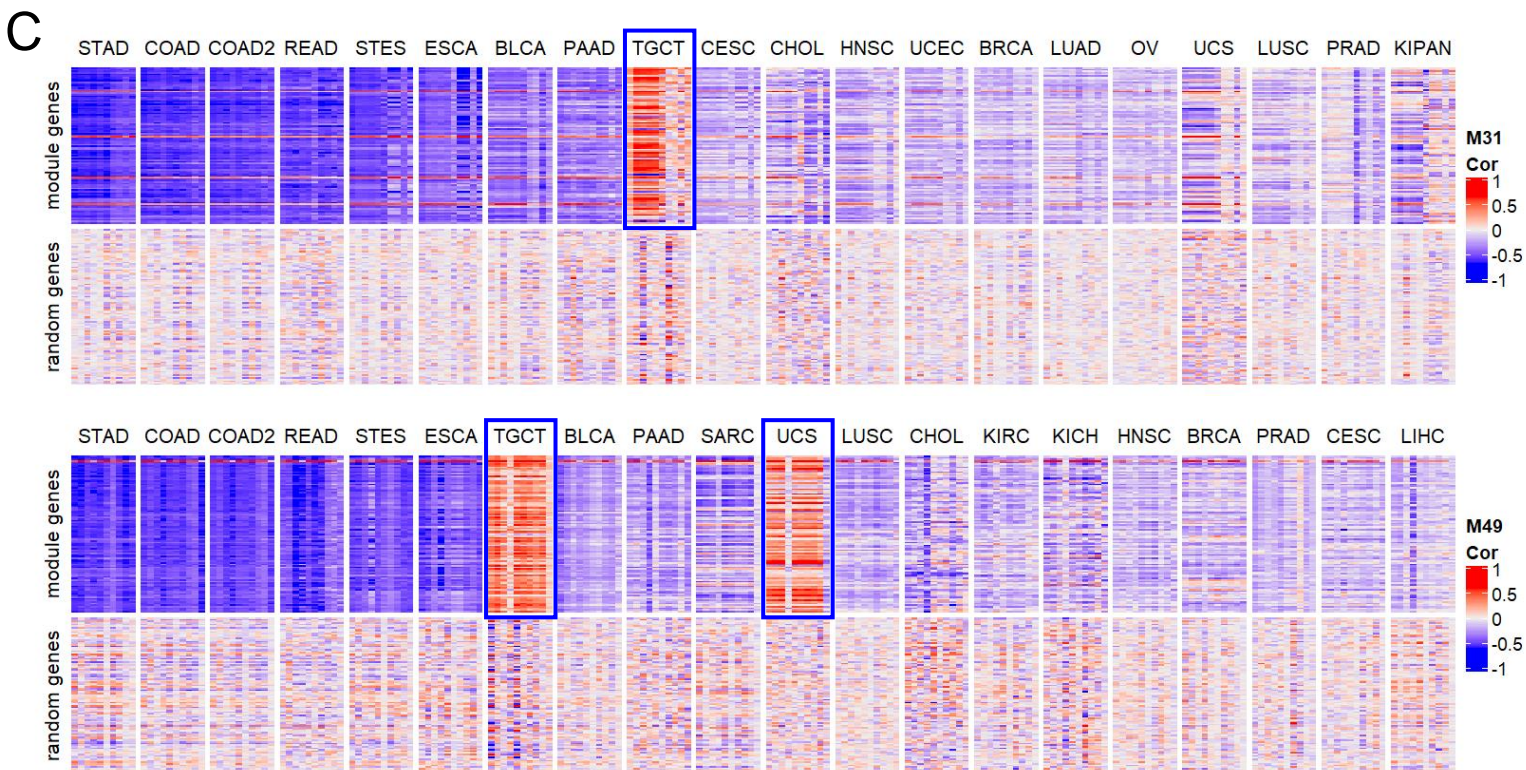

Supplement: S3 Fig — (A) Heatmap showing the output matrix W of Algorithm 2, when it was applied to the TCGA data. Each column corresponds to a module and each row corresponds to a cancer type and Wij reflects the co-expressed intensity between the genes and the miRNAs within the module j on the cancer i. A hierarchical clustering method was used to cluster the rows (cancer types) into four clusters. (B) Scatter plot for elements of the W matrix. There are three negative elements/pairs in W, where (Module 31, TGCT) is −0.145, (Module 49,TGCT) is −0.23 and (Module 49, UCS) is −0.138 and (C) Their heatmaps shown in the blue frame. (PDF) [file pcbi.1009044.s004.pdf]

A

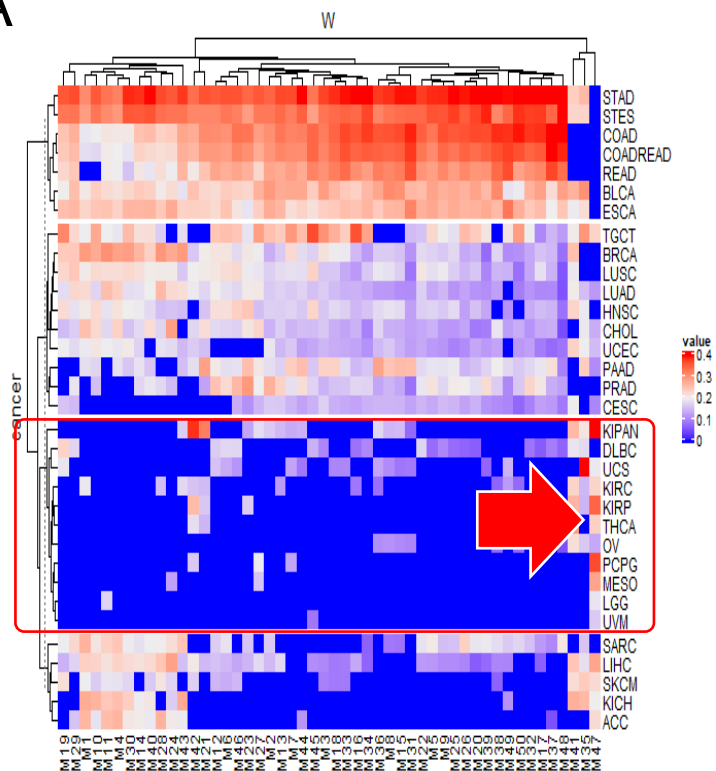

B

Result on a subset of cancers

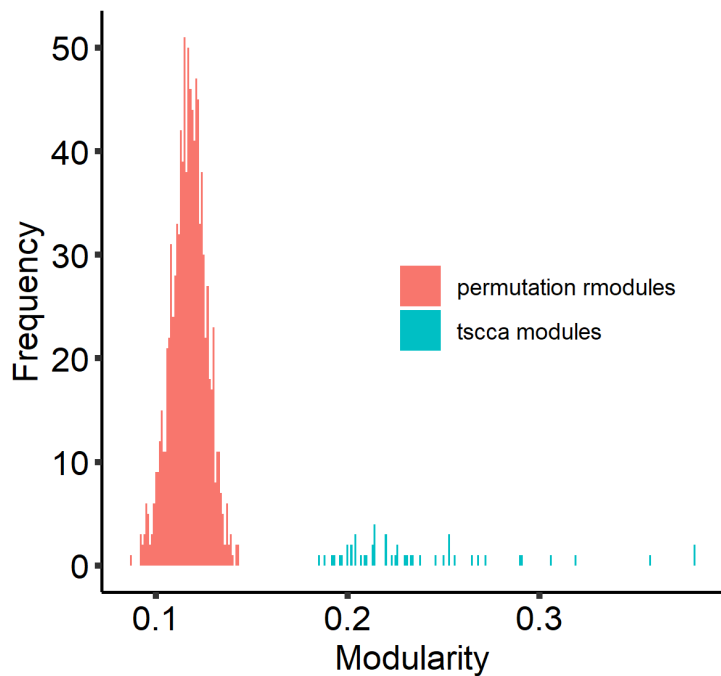

C

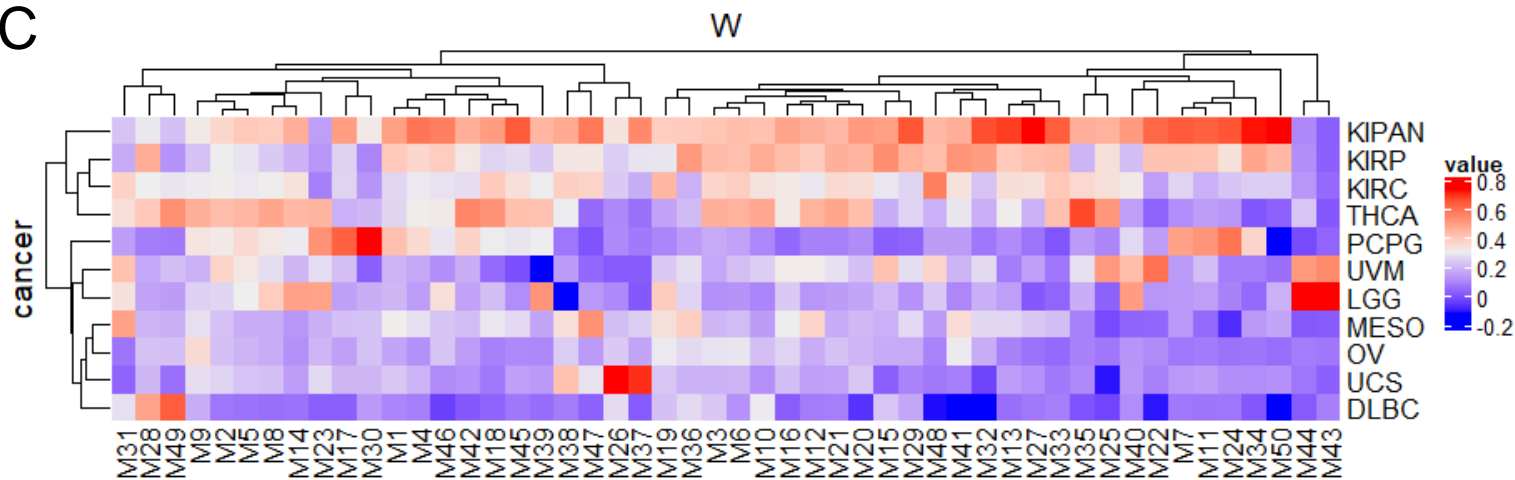

Supplement: S4 Fig — We first extracted a subset of cancers (A) and then re-used TSCCA to extract 50 modules on the subset of the previous data, and we found some new modules with significant modularity scores (B). Finally, we show the heatmap of the corresponding W matrix (C). (PDF) [file pcbi.1009044.s005.pdf]

# A. TSCCA

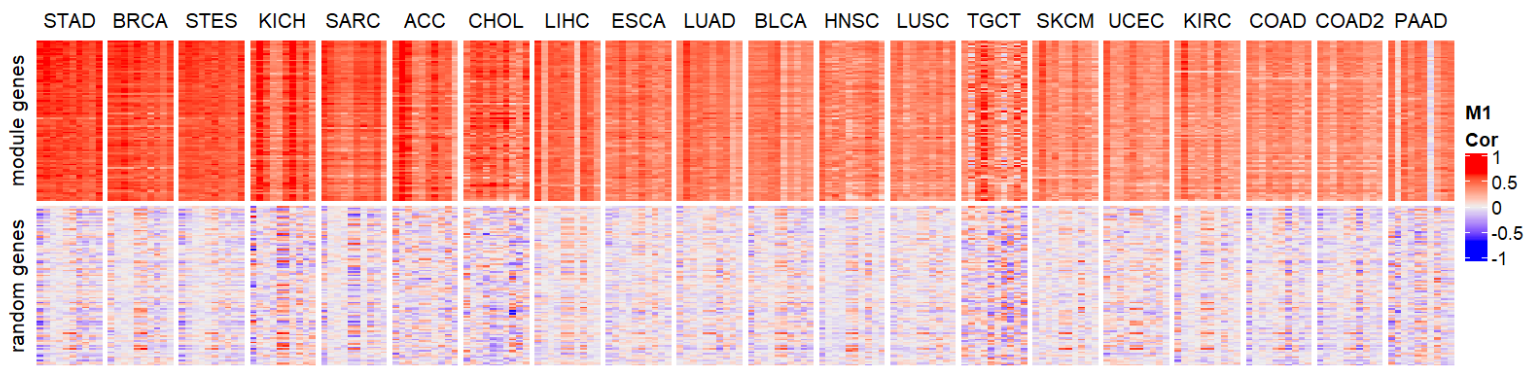

# B. Modularity\_SA

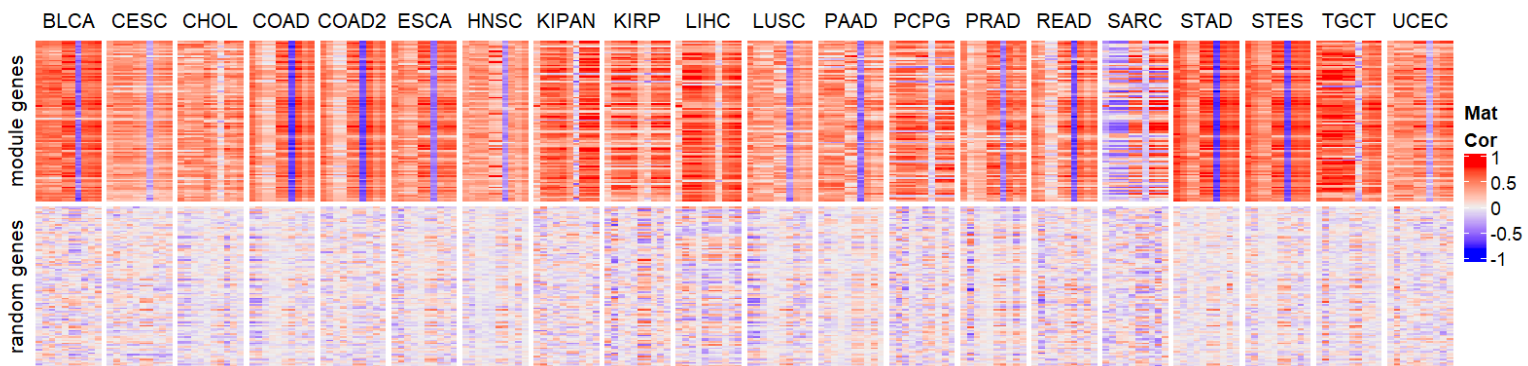

# C. Var\_SA

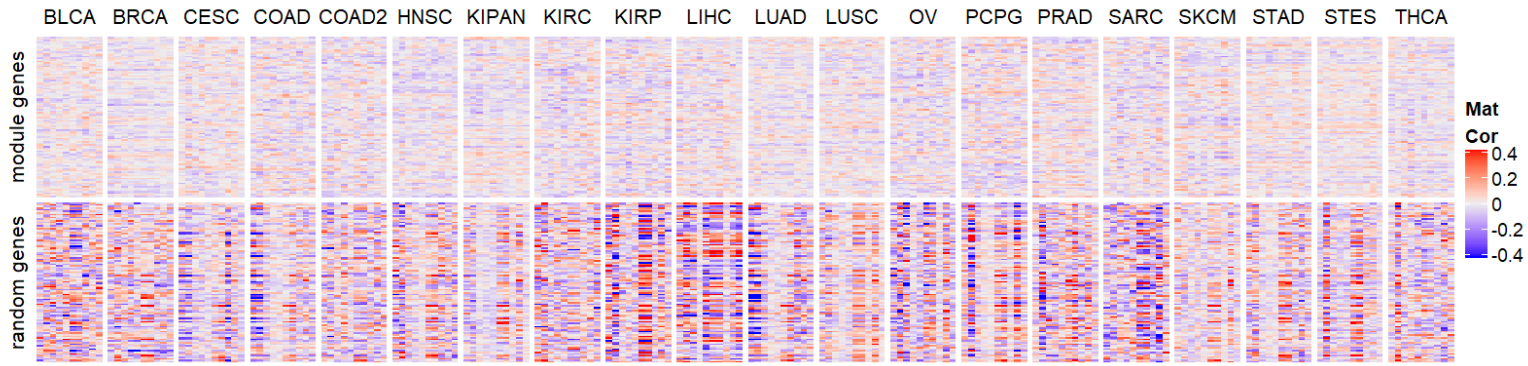

# D. MSR\_SA

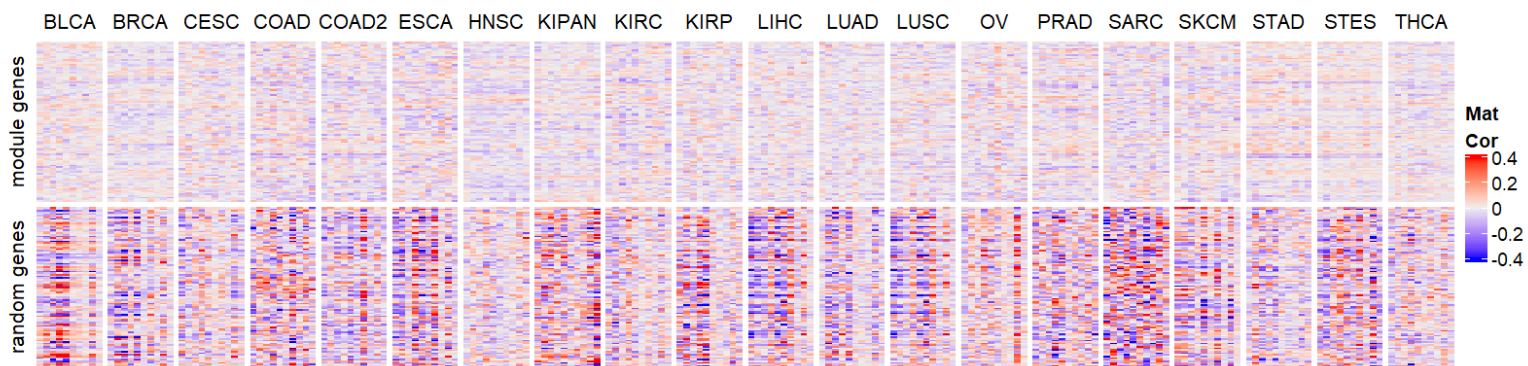

Supplement: S6 Fig — The top half of each heatmap corresponds to the module 1 (row corresponds to gene, column corresponds to miRNA) and the lower part is a random module for comparison. (PDF) [file pcbi.1009044.s007.pdf]

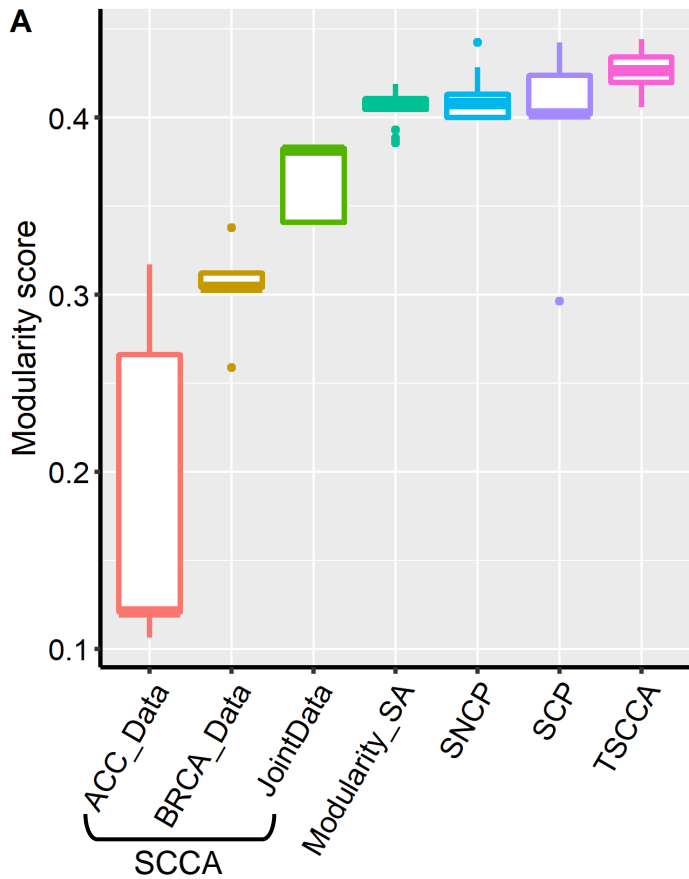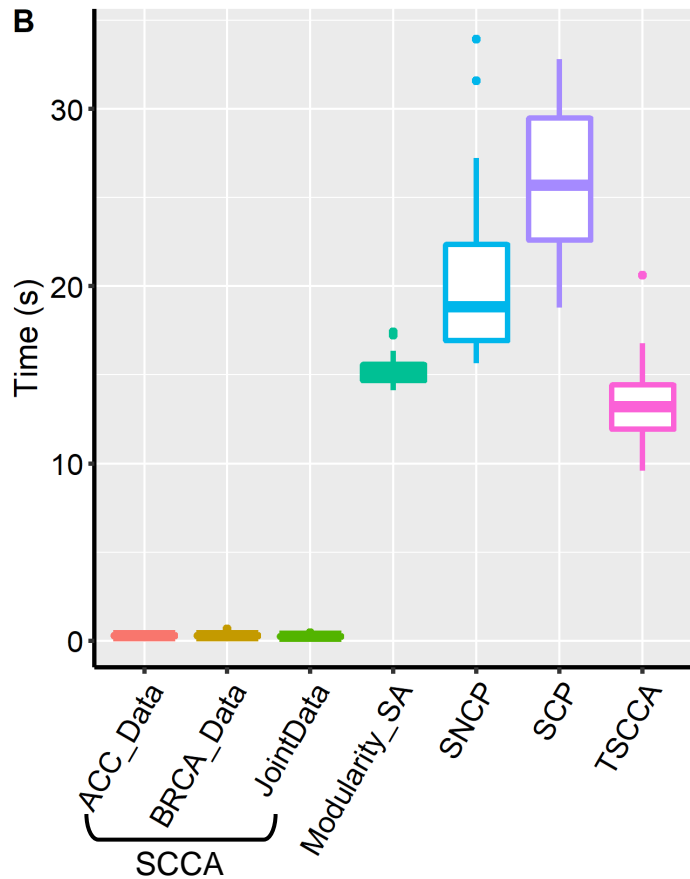

Supplement: S7 Fig — We also compared the running time of different methods on a personal laptop. Box-plots show results in terms of modularity scores and running time of algorithm based on 50 different initializations of each method. (PDF) [file pcbi.1009044.s008.pdf]

## A. Heatmap of pcModule

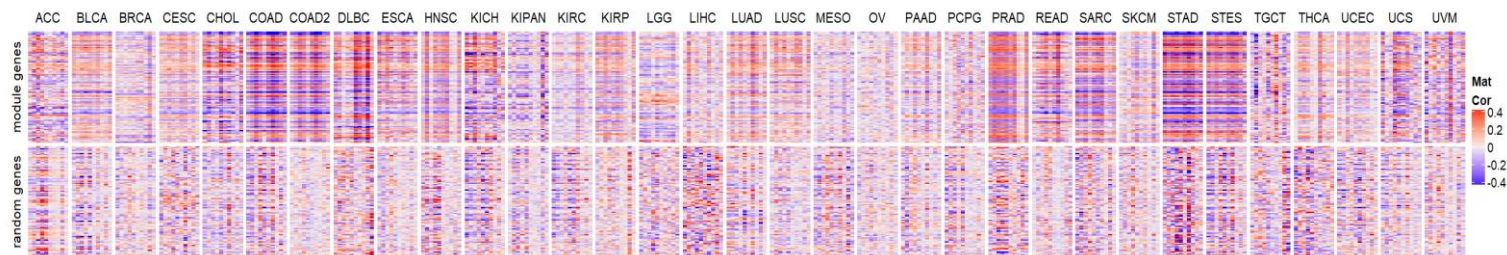

## B

Histogram

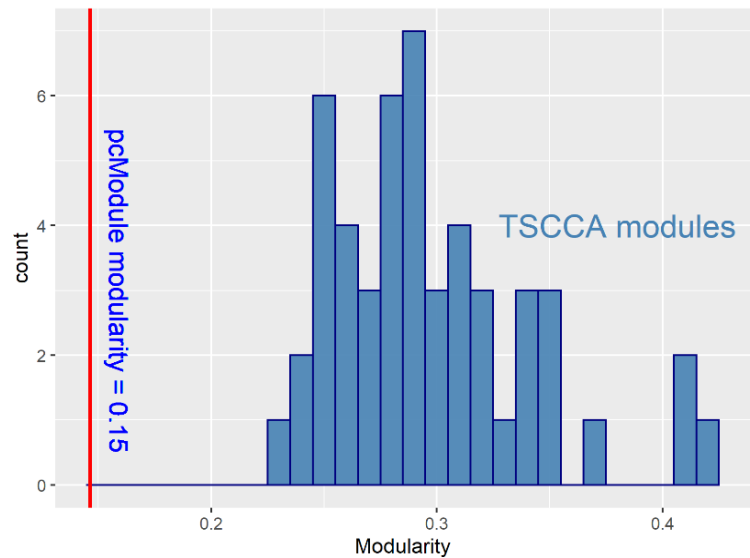

Supplement: S8 Fig — (A) Heatmap of pcModule. The top half of each heatmap corresponds to the module 1 (row corresponds to gene, column corresponds to miRNA) and the lower part is a random module for comparison. (B) Comparison of modularity scores of pcModule and TSCCA modules. (PDF) [file pcbi.1009044.s009.pdf]

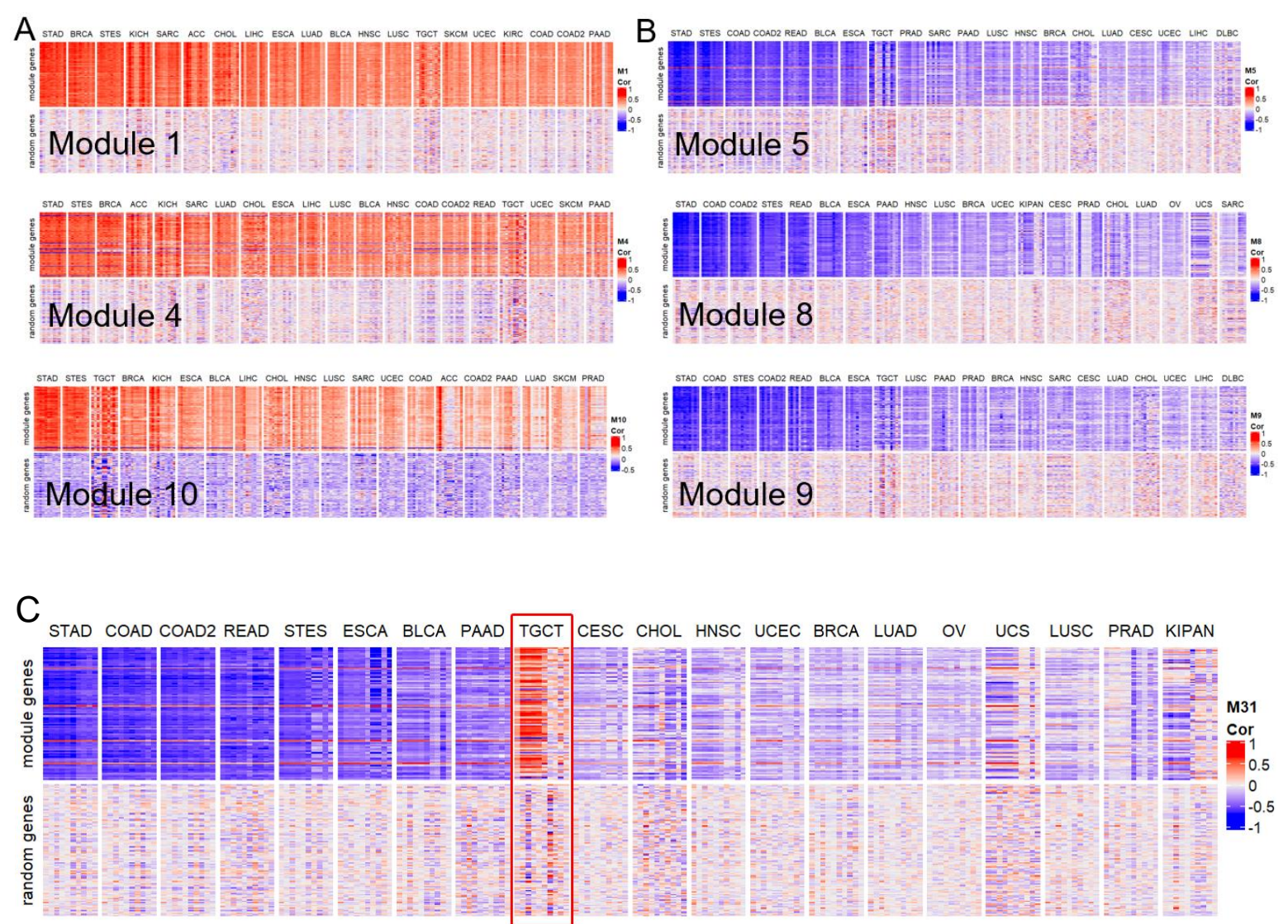

Supplement: S9 Fig — (A) Heatmap of modules 1, 4 and 10. (B) Heatmap of modules 5, 8 and 9. (C) Heatmap of cancer-miRNA-gene module 31 identified by TSCCA in the TCGA dataset. Module 31 is a TGCT-cancer-specific miRNA-gene co-expressed module. (PDF) [file pcbi.1009044.s010.pdf]
