# Supplementary material for: TSCCA: A tensor sparse CCA method for detecting microRNA-gene patterns from multiple cancers
Source: PLoS Comput Biol. 2021 Jun 1;17(6):e1009044. doi: 10.1371/journal.pcbi.1009044 (PMC8195367; doi:10.1371/journal.pcbi.1009044)
Supplement: S5 Fig — (A) For each identified module, a produce is developed to identify a largest connected subgraph, i.e., a three-layer miRNA-gene regulatory network, where the miRNA-gene interactions are from miRTarBase network and the gene-gene interactions are from the gene interaction network, and miRNAs regulate genes and these genes regulate the other genes with three-layer network. (B) A miRNA-gene network contains 3619 experimentally verified miRNA-gene interactions from miRTarBase network via combing all genes and miRNAs of modules identified by TSCCA (Hypergeometric test P = 3.5e-43). (PDF) [file pcbi.1009044.s006.pdf]

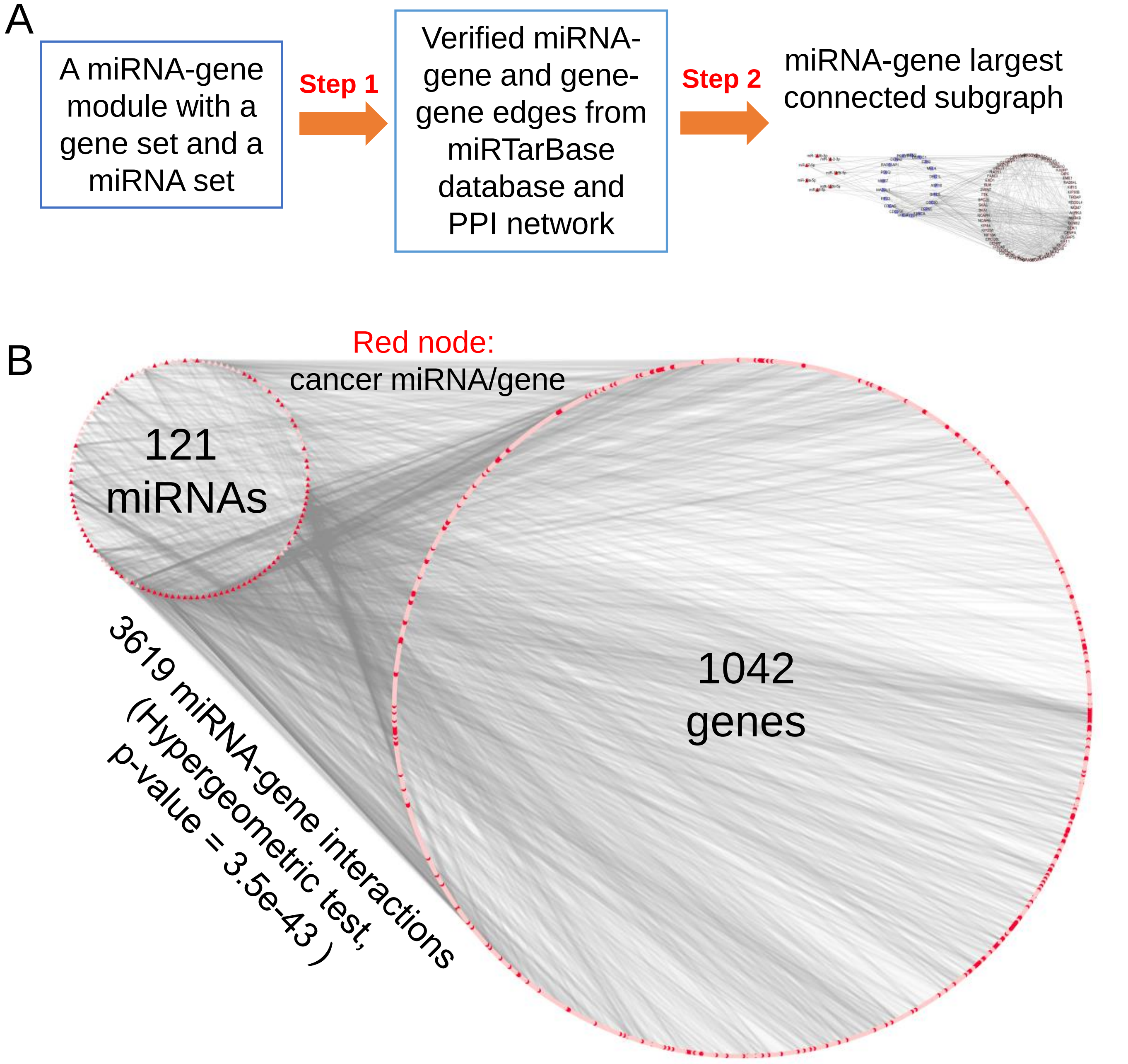
A
A miRNA-gene module with a gene set and a miRNA set
Step 1
Verified miRNA-gene and gene-gene edges from miRTarBase database and PPI network
Step 2
miRNA-gene largest connected subgraph
B
Red node:
cancer miRNA/gene
121 miRNAs
1042 genes
3619 miRNA-gene interactions
(Hypergeometric test,
p-value = 3.5e-43)
