# Supplementary material for: TSCCA: A tensor sparse CCA method for detecting microRNA-gene patterns from multiple cancers
Source: PLoS Comput Biol. 2021 Jun 1;17(6):e1009044. doi: 10.1371/journal.pcbi.1009044 (PMC8195367; doi:10.1371/journal.pcbi.1009044)
Supplement: Attachment — Submitted filename: Response_Letter.pdf [file pcbi.1009044.s039.pdf]

Response to Reviewer: 1

The paper presents TSCCA, a novel algorithm for identification of miRNA-gene modules across multiple cancer types from TCGA. TSCCA is based on CCA and extends it to tensors to support several cancer types. The authors apply the method to 33 cancers from TCGA, and show the biological relevance of the detected modules using several databases of biological knowledge, and by considering the survival implications of the selected genes and miRNA. The authors also compare TSCCA to Modularity SA and to SCCA on cancer and synthetic data. The task of detecting miRNA-gene modules in cancer is of high importance, given the known involvement of miRNA in cancer and the complex regulatory processes that are perturbed in cancer. We are not familiar with previous methods designed to find miRNA-gene modules across several datasets, so this method has potentially high value. The paper is well organized and well written. However, as previous methods detected miRNA-gene modules, and the main innovation of this method is in working on multiple datasets, the benefit of using multiple cancers should be shown in a more convincing manner. Additionally, while no algorithm was previously developed for the specific data used in the study, the problem of tri-clustering (detecting modules in tensors) was previously investigated, and the suggested method should be compared to other tri-clustering methods, in addition to modularity SA. Furthermore, the authors should provide an implementation of TSCCA. If these points are satisfactorily addressed, we think that this work would merit publication in PLOS Computational Biology.

Response: We deeply thank the reviewer for the very positive, constructive comments and suggestions. In the revised manuscript and the following response, we address the above points in detail.

Major comments

1. Previous methods were developed to detect miRNA-gene modules, and the main novelty of TSCCA is that it uses multiple datasets. The advantage of using multiple datasets is not clearly demonstrated. The criteria used to show the biological and clinical relevance of the detected modules (enrichment of cancer genes and miRNAs in the modules, enrichment of miRNA families, miRNA-gene regulatory networks, and survival analysis using the modules) are never compared to solutions obtained on a single cancer dataset. It is very likely that all of these criteria would also show biological relevance if SCCA would be applied to each cancer separately.

Response: Yes, we agree that SCCA could also identify functionally enriched modules. However, it cannot well capture multiple cancer shared or several cancer-specific modules in an effective one-time manner. In light of your comment, we have used SCCA to identify 50 modules on each cancer data set (**Table S24**). For a single cancer data, SCCA did ensure that the expression of

miRNAs and genes within each identified module are correlated in the specific cancer data (see **8th column in Table S24**), but it failed to ensure that the miRNAs and genes with the identified module are correlated in most cancer types (see **7th column in Table S24**). To clarify this, we have added this result into **Supplementary Section S22:** *"More details about comparison of TSCCA with other methods"*, and added the discussions into main text (**lines 455-465**).

"In this section, we compared TSCCA with SCCA and multiple tri-clustering methods on the TCGA data. Firstly, we used SCCA to identify 50 modules on each cancer data set and compared TSCCA with SCCA in terms of modularity scores and multiple biological indicators (S3 Appendix Table S24). The parameters of SCCA is consistent with the parameters of TSCCA with $k_u = 200$, $k_v = 10$ and $k_w = 20$ when applying to the TCGA data. For a single cancer data, SCCA also ensures that the expression of miRNAs and genes within the identified modules are correlated in the specific cancer data (see the eighth column in S3 Appendix Table S24), but it failed to ensure that the miRNAs and genes with the identified modules are correlated in most cancer types (see the seventh column in S3 Appendix Table S24). Thus, TSCCA is more suitable to multi-cancer data compared to SCCA."

Table S24. Performance comparison of TSCCA and SCCA, where we used SCCA to identify 50 modules on each cancer data set. "#cancer miR", "#cancer gene", "#gene edge" and "#miR-gene edge" denote the average of the number of cancer miRNAs, cancer genes, gene edges and miRNA-gene edges on all the identified modules. Since SCCA cannot select cancer types when applying to single cancer data, we assumed "Modularity" of SCCA is computed on all 33 cancer types, while "Single cancer modularity" of SCCA is computed on a cancer type.

Data	Method	#cancer miR	#cancer gene	#gene edge	#miR-gene edge	modularity	single cancer modularity
**33 cancers**	**TSCCA**	**5.98**	**19.56**	**106.10**	**22.70**	**0.30**	
**ACC**	SCCA	5.10	17.96	74.30	14.72	0.15	0.43
**BLCA**	SCCA	5.92	20.54	62.84	13.22	0.21	0.43
**BRCA**	SCCA	4.44	18.36	87.86	21.22	0.19	0.40
**CESC**	SCCA	5.00	18.42	48.90	14.20	0.18	0.33
**CHOL**	SCCA	5.26	19.04	53.14	14.38	0.16	0.44
**COAD**	SCCA	5.66	17.70	43.12	15.30	0.20	0.49
**COADREAD**	SCCA	5.54	17.60	44.32	14.92	0.20	0.47
**DLBC**	SCCA	5.04	16.52	55.70	16.96	0.14	0.47
**ESCA**	SCCA	5.46	16.52	48.36	15.14	0.18	0.47
**HNSC**	SCCA	5.94	18.24	80.16	20.32	0.19	0.36
**KICH**	SCCA	5.80	17.72	46.24	11.22	0.16	0.52
**KIPAN**	SCCA	6.26	17.96	40.62	15.32	0.17	0.53
**KIRC**	SCCA	6.42	19.42	56.20	17.68	0.18	0.35
**KIRP**	SCCA	5.86	17.98	51.22	13.48	0.18	0.39
**LGG**	SCCA	5.54	13.40	54.74	10.42	0.14	0.53
**LIHC**	SCCA	5.54	20.60	78.40	15.42	0.18	0.43
**LUAD**	SCCA	5.58	18.24	100.68	21.16	0.19	0.35
**LUSC**	SCCA	6.44	21.64	72.36	20.62	0.20	0.34
**MESO**	SCCA	6.02	19.60	61.96	12.40	0.16	0.39
**OV**	SCCA	5.66	19.16	63.58	19.12	0.18	0.29
**PAAD**	SCCA	6.22	20.12	55.64	13.54	0.16	0.46
**PCPG**	SCCA	6.10	18.72	54.20	15.18	0.17	0.44

**PRAD**	SCCA	6.96	17.04	49.34	21.14	0.19	0.40
**READ**	SCCA	5.52	17.52	43.70	16.32	0.19	0.46
**SARC**	SCCA	5.48	15.76	54.42	12.36	0.16	0.47
**SKCM**	SCCA	5.58	19.92	61.96	13.86	0.18	0.34
**STAD**	SCCA	5.68	17.26	58.28	19.38	0.22	0.53
**STES**	SCCA	5.66	17.28	65.94	18.28	0.22	0.47
**TGCT**	SCCA	5.36	18.30	37.84	12.86	0.16	0.66
**THCA**	SCCA	6.28	19.40	46.04	14.52	0.17	0.46
**UCEC**	SCCA	5.16	19.36	88.20	19.50	0.19	0.32
**UCS**	SCCA	6.20	15.92	42.22	10.32	0.15	0.46
**UVM**	SCCA	6.06	16.76	41.72	11.34	0.15	0.48

The multi-dataset nature of the algorithm is currently considered in two places. The first is Figure 4, where the W matrix is visualized. This matrix shows that the modules actually capture only a subset of the cancer types, while other cancer types have many zero or near-zero loadings in W. To these reviewers, it seems surprising that all these cancer types have such a small number of miRNA-gene modules. Is this a biological reality or a bias introduced by the algorithm? The authors should rerun TSCCA after excluding the few cancer types that are responsible for most of the modules, and examine the output of the algorithm. If new biologically significant modules emerge, this would suggest that dominant caner types overshadow the others. In this case this limitation of the algorithm should be clearly stated.

Response: This is a very interesting point. We think it could be possible to identify more modules. We note that TSCCA is an explorative tool, which identity the "strongest" modular patterns in the current data. In light of your suggestion, we first extracted a subset of cancers (from the cluster 3 of **Figure S16A**). We then re-used TSCCA to extract 50 modules on the subset of the previous data (across 18 cancers), and we found some new modules with significant modularity scores (**Figure S16B**). Further, we show the heatmap of the corresponding W matrix (**Figure S16C**), suggesting that TSCCA could still find new significant modules.

Finally, we have discussed this point into the main text (**lines 527-534**) as follows:

"We note that TSCCA is an explorative tool, which identity the "strongest" modular patterns in the current multiple cancer data. This means that in a subset of it, it could identify more significant modules. For example, most of the 50 modules identified by TSCCA on the TCGA dataset are enriched in 60% of cancers, while other cancers are rare. To this end, we may extract a subset of cancers from the cluster 3 in ***Fig 4*** *and then re-use TSCCA to extract some modules on a subset of the previous data (across 18 cancers). More details of and results are given in* ***Figure S16****."*

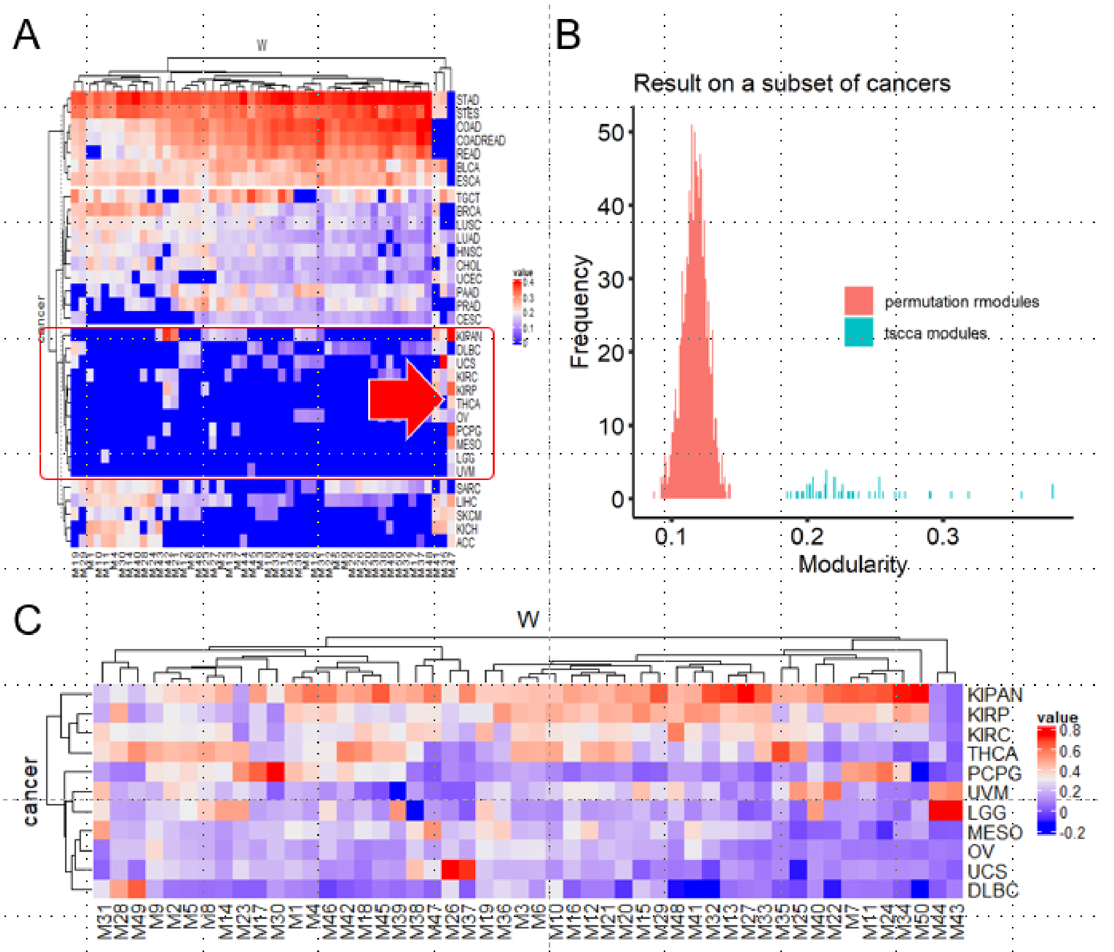

Figure S16. Application of the TSCCA onto the subset of TCGA cancer data from the cluster 3 in Fig. 4 and extract 50 modules.

The second place where the multi-dataset aspect is considered is the direct comparison to SCCA. This analysis is more convincing, but it should be expanded in two ways. First, biological criteria should be used for the comparison (enrichment of cancer genes etc.), and not only the modularity. Second, it is interesting to show the modularity when calculated only on one cancer type (e.g. ACC) when TSCCA is applied to all the data, and SCCA is applied only on ACC. This can show when using one dataset to detect miRNA-gene modules is preferable to using multiple datasets, highlighting the limitations of the algorithm.

Response: In light of your suggestion, we also have used SCCA to identify 50 modules on each cancer data. And we compared TSCCA with SCCA from multiple biological indicators (see above **Table S24**).

Firstly, compared with TSCCA, although SCCA can ensure that the average modularity score of identified modules on a single cancer is larger in that cancer, SCCA cannot ensure these discovered miRNA-gene modules are co-expressed in multiple cancers, i.e., the miRNAs and genes within the module are co-expressed in a single cancer, and it cannot ensure that they are co-

expressed on other cancers. This is the shortcoming of SCCA. Secondly, compared to SCCA, TSCCA has some advantages in multiple biological indicators, such as the average number of cancer miRNAs, cancer genes, gene edges and miRNA-gene edges (**Table S24**). For a single cancer data, SCCA did ensure that the expression of miRNAs and genes within each identified module are correlated in the specific cancer data (see **8th column in Table S24**), but it failed to ensure that the miRNAs and genes with the identified modules are correlated in most cancer types (see **7th column in Table S24**).

Finally, in the revised manuscript, we have added these new results into **Supplementary Section S22** "*More details about comparison of TSCCA with other methods*" and added more discussion in the main text (**lines 455-465**).

To the best of our knowledge, no previous method was developed to detect miRNA-gene modules across multiple datasets, but other methods were developed for a similar computational task – triclustering. The only triclustering algorithm TSCCA is compared to is Modularity_SA, which is a method that the authors developed themselves for the comparison. The authors should compare TSCCA to other triclustering methods, even if they do not perform l0 regularization. A survey of triclustering algorithms can be found in [1]. As stated in the previous point, the comparison should include biological criteria. The authors demonstrate convincingly that TSCCA's modules are biologically relevant, but biological criteria (e.g. enrichment of gene-gene interactions) should be compared to other methods.

Response: We thank the reviewer for comment. We have compared TSCCA with multiple tri-clustering methods including modularity_SA and Sparse Canonical Polyadic decomposition (SCP) which uses l1 regularization to force sparsity [1], and two merit-function based methods including "Variance" (Eq. 1 in [2]) and "Mean squared residue (MSR)" (Eq. 3 in [2]). These two merit-functions are optimized by using annealing algorithm. Var_SA is a variance based simulated annealing (Var_SA) method, which uses a simulated annealing algorithm to minimize the variance index for extracting a cancer-miRNA-gene module. Similarly, MSR_SA is an MSR based simulated annealing (MSR_SA) method, which uses a simulated annealing algorithm to minimize the MSR index for extracting a cancer-miRNA-gene module.

[1] G. Allen, "Sparse higher-order principal components analysis," in Artificial Intelligence and Statistics, 2012, pp. 27–36.
[2] R. Henriques and S. C. Madeira, "Triclustering algorithms for three-dimensional data analysis: a comprehensive survey," ACM Computing Surveys (CSUR), vol. 51, no. 5, pp. 1–43, 2018.

The comparison results are given in **Table S25** and show that TSCCA is superior to the other tri-clustering methods on multiple biological indicators and modularity score. Due to the definition of MSR, the MSR_SA method is very

consuming time. We found that MSR_SA took an hour to identify a module, while Var_SA only takes 5 seconds on a personal computer. Compared with the TSCCA and Modularity_SA, the corresponding sub-tensors of modules identified by Var_ SA or MSR_SA tend to be zero patterns (**Figure S14**). In short, TSCCA is superior to other tri-clustering methods in multiple indicators.

Table S25. Performance comparison of TSCCA and the triclustering methods. "#cancer miR", "#cancer gene", "#gene edge" and "#miR-gene edge" denote the average of the number of cancer miRNAs, cancer genes, gene edges and miRNA-gene edges on all the identified modules.

Method	#cancer_miR	#cancer_gene	#gene edge	#miR-gene edge	Modularity
**TSCCA**	**5.98**	**19.56**	**106.10**	**22.70**	**0.30**
**SCP**	5.80	12.54	67.98	14.92	0.29
**Modularity_SA**	8.08	21.14	47.52	11.62	0.29
**Var_SA**	3.40	13.58	32.04	4.94	0.04
**MSR_SA**	3.48	12.30	32.22	7.14	0.06

In the revised manuscript, we have added these new results into **Supplementary Section S22** *"More details about comparison of TSCCA with other methods"* and added the discussions into main text (**lines 466-483**).

"*Secondly, we also compared TSCCA with multiple tri-clustering methods including Modularity_SA and Sparse Canonical Polyadic decomposition (SCP) which uses ℓ_1-regularization to force sparse [49], and two merit-function based methods including "Variance" (see Eq. 1 in [50]) and "Mean squared residue (MSR)" (see Eq. 3 in [50]). The two merit-functions are optimized by using annealing algorithm. Var_SA is a variance-based simulated annealing (Var_SA) method, which uses a simulated annealing algorithm to minimize the variance merit-function for extracting a cancer-miRNA-gene module. Similarly, MSR_SA is an MSR-based simulated annealing (MSR_SA) method, which uses a simulated annealing algorithm to minimize the MSR merit-function for extracting a cancer-miRNA-gene module. The comparison results are given in S3 Appendix Table S25 and show that TSCCA is superior to the other tri-clustering methods in terms of multiple biological indicators and modularity score. Due to the definition of MSR, the MSR_SA method is very consuming time. We found that MSR_SA took an hour to identify a module, while Var_SA only takes 5 seconds on a personal computer. Compared with the TSCCA and Modularity_SA, the sub-tensors/modules identified by Var_SA or MSR_SA tend to be zero patterns (S2 Appendix Fig S14). In short, the TSCCA method is superior to other tri-clustering methods in terms of multiple indicators (S3 Appendix Table S25).*"

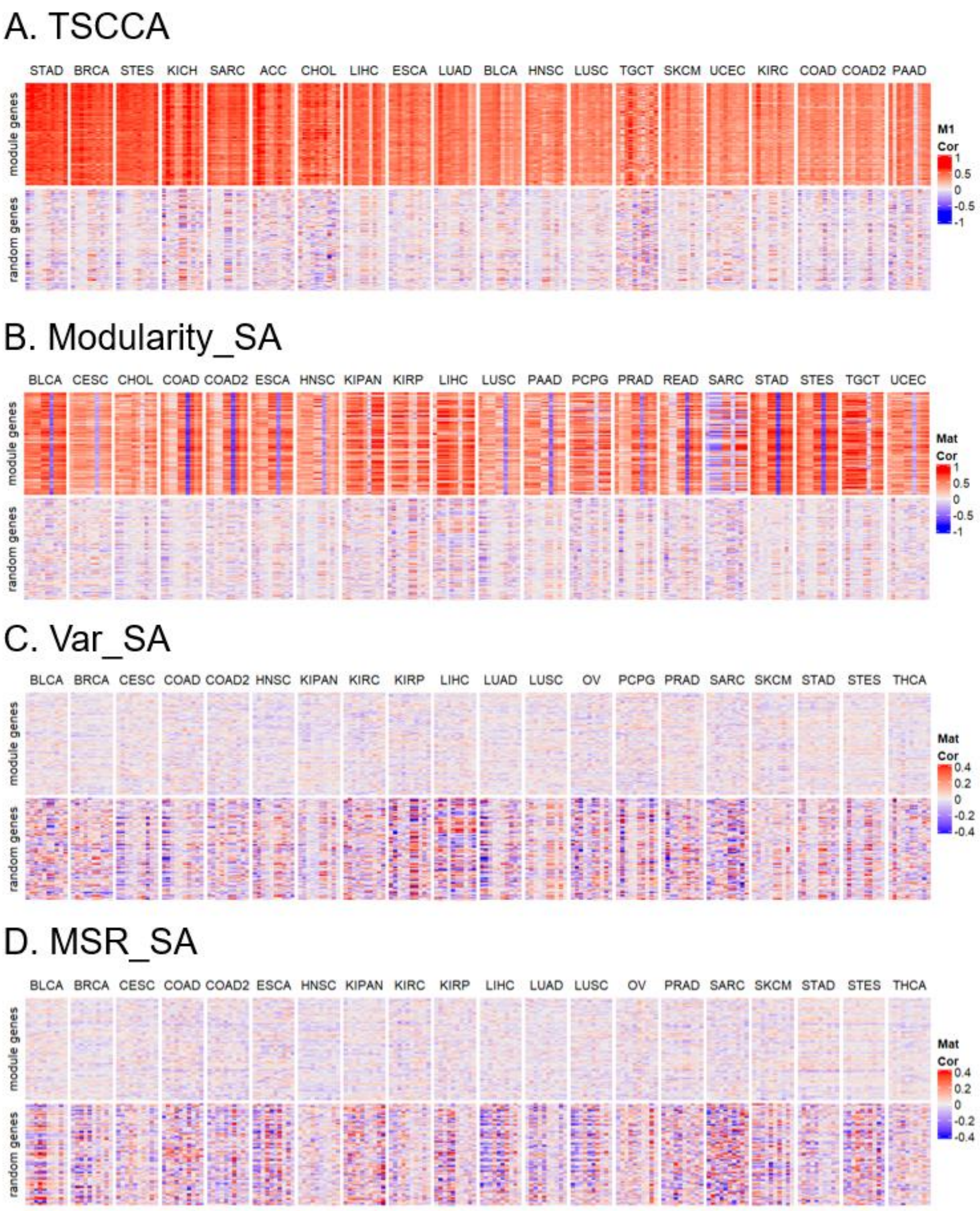

Figure S14. Heatmap of cancer-miRNA-gene modules identified by different methods in the TCGA dataset. The top half of each heatmap corresponds to the module 1 (row corresponds to gene, column corresponds to miRNA) and the lower part is a random module for comparison.

3. The authors should provide an implementation (or at least a well documented executable) of TSCCA, as the method, rather than the biological results, are the main contribution of this work.

Response: In light of your suggestion, we uploaded the R package for TSCCA at the GitHub repository https://github.com/wenwenmin/TSCCA and provided the guide for potential users.

Other important comments

4. In order to optimize TSCCA's objective function (equation (5)), the authors optimize for u, v and w separately. If w is held constant, the optimization problem is that of sparse diagonal CCA, which is solved by the work of Xu et al. that the authors cite (reference [38] in the paper). Their algorithm can be used for an improved optimization procedure.

Response: We appreciate very much for your comment. Since the TSCCA's objective function of the optimization problem we solved in (5) is a linear problem with respect to ***u*** or ***v***. In fact, the accelerated version of this algorithm based on reference [38] is the same as the algorithm we proposed. Below we prove this.

In the Proposition 3 of **Supplementary Section S3,** we proved that

$$\boldsymbol{u}^{k+1} := P_{R_u}\left(\boldsymbol{u}^k - \frac{1}{L_u}\nabla_u f(\boldsymbol{u}^k, \boldsymbol{v}^k, \boldsymbol{w}^k)\right) = P_{R_u}(-\bar{\boldsymbol{A}} \times_2 \boldsymbol{v}^k \times_3 \boldsymbol{w}^k) = \frac{\Pi(\boldsymbol{z}_u, k_u)}{\|\Pi(\boldsymbol{z}_u, k_u)\|}$$

where $\boldsymbol{z}_u = \bar{\boldsymbol{A}} \times_2 \boldsymbol{v}^k \times_3 \boldsymbol{w}^k$. In other words, we obtain the update formula of $\boldsymbol{u}$:

$$\boldsymbol{u}^{k+1} := \frac{\Pi(\boldsymbol{z}_u, k_u)}{\|\Pi(\boldsymbol{z}_u, k_u)\|} \quad (1)$$

Below we consider the acceleration situation in the reference [38]. Based on the proposed framework in reference [38], the improved algorithm with the extrapolation for $\boldsymbol{u}$ is

$$\boldsymbol{u}^{k+1} := P_{R_u}\left(\hat{\boldsymbol{u}}^k - \frac{1}{L_u}\nabla_u f(\boldsymbol{u}^k, \boldsymbol{v}^k, \boldsymbol{w}^k)\right) = P_{R_u}(-\bar{\boldsymbol{A}} \times_2 \boldsymbol{v}^k \times_3 \boldsymbol{w}^k) = \frac{\Pi(\boldsymbol{z}_u, k_u)}{\|\Pi(\boldsymbol{z}_u, k_u)\|}$$

where $\hat{\boldsymbol{u}}^k = \boldsymbol{u}^k + \omega_k(\boldsymbol{u}^k - \boldsymbol{u}^{k-1})$. L_u is the Lipschitz constant of $\nabla_u f(\boldsymbol{u}^k, \boldsymbol{v}^k, \boldsymbol{w}^k) = -\bar{\boldsymbol{A}} \times_2 \boldsymbol{v}^k \times_3 \boldsymbol{w}^k$. Since $\nabla_u^2 f = 0$, L_u can be any constant greater than zero. When $L_u \to 0$, we also have

$$\boldsymbol{u}^{k+1} := P_{R_u}\left(\hat{\boldsymbol{u}}^k - \frac{1}{L_u}\nabla_u f(\boldsymbol{u}^k, \boldsymbol{v}^k, \boldsymbol{w}^k)\right) = P_{R_u}(-\bar{\boldsymbol{A}} \times_2 \boldsymbol{v}^k \times_3 \boldsymbol{w}^k) = \frac{\Pi(\boldsymbol{z}_u, k_u)}{\|\Pi(\boldsymbol{z}_u, k_u)\|}$$

where $\boldsymbol{z}_u = \bar{\boldsymbol{A}} \times_2 \boldsymbol{v}^k \times_3 \boldsymbol{w}^k$. Thus, we obtain the update formula of $\boldsymbol{u}$ in the improved algorithm is

$$\boldsymbol{u}^{k+1} := \frac{\Pi(\boldsymbol{z}_u, k_u)}{\|\Pi(\boldsymbol{z}_u, k_u)\|} \quad (2)$$

We can see that Eqs (1) and (2) are the same. Therefore, the algorithm of the accelerated version is the same as the proposed Algorithm 1 for the problem (5) in this paper.

5. The analysis for whether the 50 modules significantly overlap is not clear.

a. Does the number of overlapping elements include genes, miRNA and cancer types? If so, it places a higher emphasis on gene expression, which is the most common feature. Or are three tests for overlap performed separately, one for genes, one for miRNA and one for cancer types? If the latter, how are these tests integrated?

b. The random modules are created by sampling 100 genes, 10 miRNA and 20

cancers. While these are the parameters used to run TSCCA, the authors state that not all modules include this number of genes, miRNA and cancers. The number of features in these random modules is therefore different from the number of features in the original modules, and the distribution of the size of the overlap is also different. The tests should be performed by conditioning on the size of the modules.
c. There seems to be a small error in the number of random modules. The appendix says 100 modules were generated, but the number of module pairs appears to be 1000 in the rest of the text.

Response: Regarding point (a), we feel sorry that we didn't show it clearly, and we clarify the definition of the overlapping elements between two modules as follows: Note each cancer-miRNA-gene module identified by TSCCA corresponds to a sub-tensor. To assess the overlap level of the two modules, we first define the overlapping elements between two modules/sub-tensors as follows: If there are two modules $(I_1, J_1, K_1) = \{(i,j,k)|\ i \in I_1, j \in J_1, i \in k_1\}$ and $(I_2, J_2, K_2) = \{(i,j,k)|\ i \in I_2, j \in J_2, i \in k_2\}$, then we defined their overlapping elements as:

$$(I_1, J_1, K_1) \cap (I_2, J_2, K_2) = \{(i,j,k)|\ (i,j,k) \in\ (I_1, J_1, K_1) \text{ and } (i,j,k) \in\ (I_2, J_2, K_2)\ \}$$

We then define the number of overlapping elements between the two modules as the number of the elements of the intersection $(I_1, J_1, K_1) \cap (I_2, J_2, K_2)$.

Regarding point (b), we have revised our program to ensure that the size of the pairs of random modules is consistent with the given pair of identified modules. Specifically, for a given pair of two identified modules (I_1, J_1, K_1) and (I_2, J_2, K_2), We first generate 1000 pairs of random modules from the original cancer-miRNA-gene correlation tensor with the same size of the given pair of modules and then compute a *p*-value for the give pair of modules using permutation test (see **Supplementary Section S7**).

Regarding point (c), Thanks. We have corrected this typo in the revised manuscript.

In light of your comments, we have revised this into **Supplementary Section S7** "Overlap analysis of any two modules using permutation test".

"7 Overlap analysis of any two modules using permutation test
Since each cancer-miRNA-gene module identified by TSCCA corresponds to a sub-tensor (Figure 1C). To assess the overlap level of the two modules, we first define the overlapping elements between two modules/sub-tensors as follows: If there are two modules $(I_1, J_1, K_1) = \{(i,j,k)|\ i \in I_1, j \in J_1, i \in k_1\}$ *and* $(I_2, J_2, K_2) = \{(i,j,k)|\ i \in I_2, j \in J_2, i \in k_2\}$*, then we defined their overlapping elements as:*

$$(I_1, J_1, K_1) \cap (I_2, J_2, K_2) = \{(i,j,k)|\ (i,j,k) \in\ (I_1, J_1, K_1)\ and\ (i,j,k) \in\ (I_2, J_2, K_2)\ \}$$

We then define the number of overlapping elements between the module

(I_1, J_1, K_1) and the module (I_2, J_2, K_2) as the number of the elements of the intersection $(I_1, J_1, K_1) \cap (I_2, J_2, K_2)$.

Based on the above definition, we adopt a permutation test method to assess the number of overlapping elements for a given pair of two identified modules (I_1, J_1, K_1) and (I_2, J_2, K_2):

Step 1. We first generate 1000 pairs of random modules from the original cancer-cancer-miRNA-gene correlation tensor with the same size of the given pair of modules.

Step 2. We then compute the number of overlapping elements between the given pair of modules, denoted by b_0. Moreover, we compute the number of overlapping elements between any pair of random modules, denoted by $b_1, \cdots, b_{1000}$.

Step 3. For the pair of two identified modules (I_1, J_1, K_1) and (I_2, J_2, K_2), a p-value is computed by using the formula:

$$p - value = \frac{\sum_{t=1}^{1000} 1(b_0 \geq b_t)}{1000}.”$$

6. In a couple of analyses the authors do not statistically show the merit of their results.
a. The authors count the number of modules with at least two miRNAs from the same family. Though 46 out of 50 sounds high, a statistical test should be performed to derive a p-value.
b. The authors found that 70% of the modules have at least 3 miRNAs participating in a three-layer network, but the significance of this observation is not clear without a statistical test.

Response: We thank the reviewer for the very detailed suggestion. For the given module set {M1, M2, …, M50} identified by TSCCA, we proposed a permutation test method to evaluate the significance level of the number of modules, which have at least two miRNAs from the same family (**Figure R1 A**).

Similarly, we also evaluated the significance level of 70% of the modules within {M1, M2, …, M50} identified by TSCCA, where each module has at least 3 miRNAs participating in a three-layer network (**Figure R1B**).

In the revised manuscript, we have rephrased the following sentence: “We found that 92% (46 out of 50) modules have at least two miRNAs in the same family (*P* < 2.2.0e-16, **S3 Appendix Table S15** and **S1 Appendix Section S17**).” and “We found that 70% modules have at least three miRNAs participating in a three-layer network (*P* < 2.2.0e-16, **S3 Appendix Table S16** and **S1 Appendix Section S17**).”

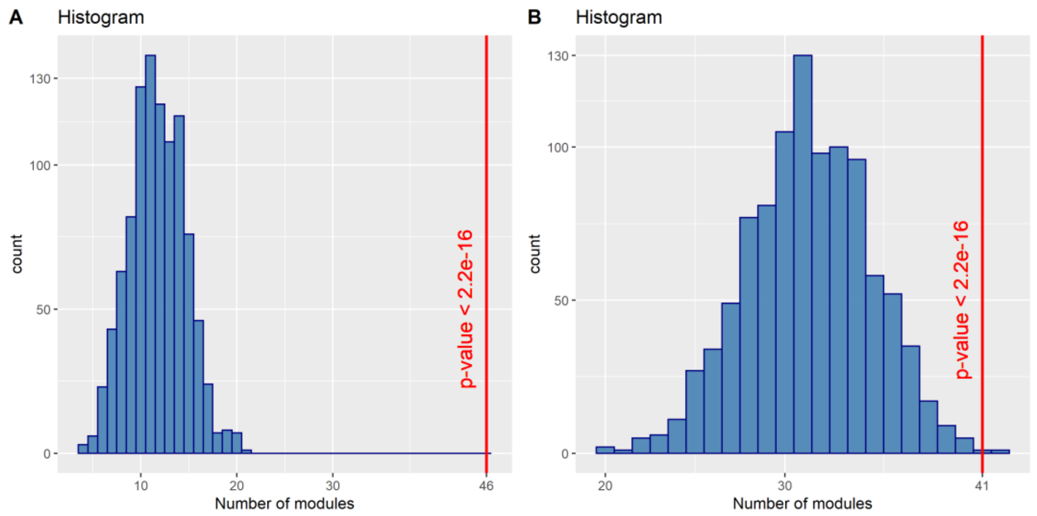

Figure R1. (A) Permutation test is used to evaluate a significance level of the number of modules, which have at least two miRNAs from the same family. (B) Permutation test is used to evaluate a significance level of 70% of the modules within {M1, M2, …, M50}, where each module has at least 3 miRNAs participating in a three-layer network

Finally, we have added this into **Supplementary Section S17** "Statistical significance analysis".

"17 Statistical significance analysis
For a given module set {M1, M2, …, M50}, we adopt a permutation test method to evaluate the significance of the number of modules from the module set with at least two miRNAs from the same family.

Step 1. Compute the number of modules, n_0*, from the given module set with at least two miRNAs from the same family.*

Step 2. We generate 1000 random module sets with the same size of the given set {M1, M2, …, M50} from the original cancer-miRNA-gene correlation tensor.

Step 3. For a random module set $i\ (i = 1,2,\dots,1000)$*, compute the number of modules,* n_i*, from the given module set with at least two miRNAs from the same family.*

Step 4. Combine n_0 *and* $n_1, \dots, n_{1000}$*, and use the one-sample Wilcoxon signed-rank test to compute the p-value of the given module set.*

Similarly, we also apply the above procedure to evaluate the significance of 70% of the modules, where each module has at least 3 miRNAs participating in a three-layer network."

7. The geometric tests used to calculate gene-gene and miRNA-gene interaction set enrichment (as described in sections 15 and 16 of the appendix) do not condition on the degree of the genes and miRNAs in the networks. In the way these tests are currently performed, it is possible to obtain significant *p*-values just because a chosen gene has high degree in the gene-gene

network (or similarly, a gene/miRNA has high degree in the gene-miRNA network), even if there's no enrichment of interaction in the module. Indeed, cancer genes are generally known to have high degrees in gene-gene networks. The tests should be performed by permuting genes/miRNAs conditioned on their degree in the networks.

Response: This is an interesting point. In light of your comment, we developed a statistical permutation test method, which was used to calculate gene-gene and miRNA-gene interaction set enrichment by permuting genes/miRNAs conditioned on their degree in the networks (**see supplementary sections S15 and S16).** We show the results of gene-gene interaction set enrichment analysis results for each identified module by TSCCA in **Table S27**. Similarly, we also show the results of miRNA-gene interaction set enrichment analysis results for each identified module by TSCCA in **Table S28**.

We have revised **Sections S15 and S16** into the Supplementary Materials and added the results into **Supplementary Section S23** "*Results of gene-gene and miRNA-gene interaction set enrichment*". In addition, we have added the discussions into main text on **lines 280-285 and 320-324**.

"In addition, to avoid the influence of degree in the gene interaction network, we developed a statistical permutation test method to perform the gene-gene interaction set enrichment, and we found that 70% (35 out of 50) modules contain significantly more gene interactions than expected by chance (permutation test P < 0.05, S1 Appendix Section S23)."

"In addition, to avoid the influence of degree for miRNAs in the miRNA-gene network, we developed a statistical permutation test method to perform the miRNA-gene interaction set enrichment (S1 Appendix Section S23). There are 20% (10 out of 50) modules, which contain significantly more miRNA-gene interactions than expected by chance (permutation test P < 0.05, S1 Appendix Section S23)."

Table S27. Gene-gene interaction set enrichment for the identified modules by TSCCA on the TCGA dataset.

Module	#gene edge	sum of gene degree	p-value by geometric test	*p*-value by permutation test
1	505	7835	0.00E+00	0.001
2	95	5144	1.13E-17	0.001
3	57	4605	2.47E-04	0.001
4	253	7738	1.03E-128	0.001
5	57	4672	2.47E-04	0.001
6	72	6906	1.31E-08	0.012
7	21	3721	9.94E-01	0.242
8	55	4608	7.03E-04	0.001
9	27	4363	9.16E-01	0.243
10	536	8688	0.00E+00	0.003

11	172	8282	1.88E-63	0.002
12	88	8939	1.25E-14	0.269
13	34	4175	5.51E-01	0.016
14	111	6268	1.88E-25	0.001
15	64	6008	3.80E-06	0.004
16	28	3524	8.84E-01	0.005
17	32	4389	6.83E-01	0.063
18	44	4302	6.41E-02	0.002
19	263	10223	1.12E-137	0.001
20	60	4532	4.53E-05	0.001
21	20	3441	9.97E-01	0.170
22	79	7991	4.49E-11	0.129
23	54	6407	1.16E-03	0.142
24	517	7827	0.00E+00	0.001
25	36	4679	4.15E-01	0.051
26	82	4869	3.26E-12	0.001
27	40	4868	1.90E-01	0.035
28	470	8471	0.00E+00	0.002
29	77	7808	2.43E-10	0.108
30	207	7845	6.50E-90	0.001
31	42	4373	1.15E-01	0.003
32	33	4370	6.18E-01	0.050
33	28	4632	8.84E-01	0.341
34	22	4293	9.90E-01	0.543
35	61	3748	2.49E-05	0.001
36	38	4624	2.91E-01	0.027
37	29	3999	8.44E-01	0.049
38	48	3926	1.60E-02	0.001
39	54	4586	1.16E-03	0.001
40	156	7338	2.20E-52	0.001
41	142	4267	2.69E-43	0.001
42	58	4965	1.42E-04	0.001
43	56	6146	4.20E-04	0.047
44	34	4509	5.51E-01	0.055
45	90	6367	1.78E-15	0.001
46	20	3682	9.97E-01	0.276
47	85	5536	2.12E-13	0.001
48	48	5003	1.60E-02	0.004
49	68	5283	2.50E-07	0.001
50	37	4779	3.51E-01	0.057

Table S28. miRNA-gene interaction set enrichment for the identified modules by TSCCA on the TCGA dataset.

Module	#miR-gene edge	sum of miR degree	***p*-value by geometric test**	*p*-value by permutation test
1	33	2363	2.55E-06	0.160
2	13	1084	5.56E-01	0.449
3	12	2465	6.65E-01	0.998
4	57	2363	1.46E-19	0.001

5	25	1909	2.18E-03	0.284
6	63	3711	1.13E-23	0.001
7	10	1031	8.47E-01	0.675
8	16	721	2.50E-01	0.147
9	42	2964	1.33E-10	0.151
10	44	2533	1.12E-11	0.014
11	36	2490	1.21E-07	0.101
12	65	3711	4.19E-25	0.001
13	12	1407	6.65E-01	0.779
14	33	1954	2.55E-06	0.048
15	10	721	8.47E-01	0.586
16	18	1295	1.17E-01	0.241
17	30	3714	4.10E-05	0.180
18	5	1031	9.97E-01	0.967
19	38	3081	1.39E-08	0.410
20	17	1750	1.75E-01	0.677
21	7	1122	9.77E-01	0.918
22	16	1018	2.50E-01	0.228
23	73	3701	4.25E-31	0.001
24	13	1192	5.56E-01	0.538
25	14	1909	4.45E-01	0.925
26	11	1073	7.64E-01	0.611
27	4	1295	9.99E-01	0.998
28	23	912	8.33E-03	0.020
29	55	3050	2.97E-18	0.022
30	56	2457	6.65E-19	0.001
31	18	1051	1.17E-01	0.133
32	25	1730	2.18E-03	0.178
33	8	1302	9.52E-01	0.936
34	9	918	9.09E-01	0.719
35	8	679	9.52E-01	0.756
36	12	678	6.65E-01	0.392
37	15	2464	3.41E-01	0.991
38	18	2097	1.17E-01	0.850
39	13	1002	5.56E-01	0.408
40	16	1228	2.50E-01	0.341
41	5	503	9.97E-01	0.918
42	2	957	1.00E+00	0.999
43	31	2261	1.68E-05	0.184
44	11	1055	7.64E-01	0.596
45	8	900	9.52E-01	0.800
46	43	3711	3.91E-11	0.001
47	15	750	3.41E-01	0.211
48	20	2128	4.62E-02	0.762

49	5	475	9.97E-01	0.915
50	2	669	1.00E+00	0.997

"15.1 Permutation test for gene-gene interaction set enrichment analysis
For a given module i, suppose it contains n_0 genes and m_0 validated gene interactions/edges and the sum of degrees of genes within the module in the original gene-gene interaction network is d_0.

Step 1. We first randomly generate 1000 modules to ensure that the sum of degree of the genes in each module is in $[d_0 - 50,\ d_0 + 50]$, which is to ensure that the sum of degree of the genes in each random module is similar to the given module, so as to eliminate the influence of the vertex degree on the result.

Step 2. We then compute the number of validated gene interactions of these random modules, denoted as $m_1, m_2, \dots, m_{1000}$.

Step 3. For the given module i and its p-value was computed by using the formula:

$$p - value\ = \frac{\sum_{i=1}^{1000} 1(m_i \geq m_0) + 1}{1000 + 1}.$$

where adding 1 to both numerator and denominator to avoid p-values of zero.

16.1 Permutation test for miRNA-gene interaction set enrichment analysis
For a given module i, suppose it contains n_0 genes and m_0 validated miRNA-gene interactions/edges and the sum of degrees of miRNAs within the module in the original miRNA-gene interaction network is d_0.

Step 1. We first randomly generate 1000 modules to ensure that the sum of degree of the genes in each module is in $[d_0 - 50,\ d_0 + 50]$, which is to ensure that the sum of degree of the miRNAs in each random module is similar to the given module, so as to eliminate the influence of the degree of miRNA on the result.

Step 2. We then compute the number of validated miRNA-gene interactions of these random modules, denoted as $m_1, m_2, \dots, m_{1000}$.

Step 3. For the given module i and its p-value is computed by using the formula:

$$p - value\ = \frac{\sum_{i=1}^{1000} 1(m_i \geq m_0) + 1}{1000 + 1}.$$

where adding 1 to both numerator and denominator to avoid p-values of zero."

8. The CE and Recovery score does not consider the cancers selected for each module. The authors need to add an additional metric (or change the current one) that considers the cancers. Additionally, because the number of miRNAs is smaller than the number of genes, it is of interest to report the CE and Recovery score when considering only genes or only miRNAs. Otherwise the current reporting places more emphasis on genes.

Response: In light of your comment, we have extended the "Recovery score"

and "CE score" to evaluate the similarity of two tri-clusters, bi-clusters and clusters. And the results of CE and recovery scores are shown in **Figure 8B**. More results are given in the **Tables S20 and S21**. For example, the results of CE and Recovery score when considering only genes or only miRNAs are given in the **2th and 3rd column of Tables S20 and S21**. In short, the TSCCA method is superior to the other methods in terms of Recovery and CE score.

In the revised manuscript, we have added more description and results into **Supplementary Section S21** "*More details about simulation study*", revised the definition of evaluation metrics into **Supplementary Section S20** "*Evaluation metrics*" and updated **Figure 8B** in main text.

Table S20. Comparison (in terms of CE ± std) on the simulated data. Since SCCA cannot select cancer types, we assumed each module identified by SCCA contained all cancer types for computing 3D-CE and 3D-recovery score.

Method	1D-CE ± std (gene)	1D-CE ± std (miR)	2D-CE ± std	3D-CE ± std
SCCA + cancer1	0.777±0.013	0.775±0.041	0.575±0.012	0.327±0.005
SCCA + cancer2	0.777±0.012	0.781±0.052	0.576±0.014	0.328±0.006
SCCA + cancer3	0.779±0.014	0.787±0.042	0.579±0.014	0.329±0.005
SCCA + cancer4	0.363±0.015	0.421±0.039	0.090±0.009	0.058±0.006
SCCA + JointData	0.370±0.015	0.429±0.039	0.090±0.009	0.058±0.006
Modularity_SA	0.596±0.159	0.653±0.202	0.506±0.153	0.506±0.153
TSCCA	**0.928±0.106**	**0.929±0.106**	**0.864±0.200**	**0.848±0.225**

Table S21. Comparison (in terms of Recovery ± std) on the simulated data.

Method	1D-recovery ±std (gene)	1D-recovery ±std (miR)	2D-recovery ±std	3D-recovery ±std
SCCA + cancer1	0.745±0.006	0.765±0.019	0.695±0.006	0.351±0.003
SCCA + cancer2	0.744±0.007	0.775±0.027	0.696±0.006	0.352±0.004
SCCA + cancer3	0.744±0.005	0.770±0.026	0.696±0.007	0.352±0.004
SCCA + cancer4	0.229±0.010	0.300±0.038	0.086±0.010	0.055±0.006
SCCA + JointData	0.232±0.009	0.300±0.032	0.084±0.008	0.055±0.005
Modularity_SA	0.770±0.019	1.000±0.000	0.770±0.019	0.770±0.019
TSCCA	**0.918±0.120**	**0.926±0.110**	**0.903±0.143**	**0.898±0.151**

The revised **Supplementary Section S20** "*Evaluation metrics*" as follows:

"20 Evaluation metrics

In the section, we use two evaluation metrics to assess module or subspace clusters, including recovery score [8] and clustering error (CE) score [9]. We also extend the "Recovery score" to evaluate the similarity of two tri-clusters, bi-clusters, and clusters. Their ranges are between 0 (low quality) and 1 (perfect).

20.1 Recovery score

Let M^{True} and M^{Pre} be two sets of modules, where M^{True} denotes the set of the k true modules and M^{Pre} denotes the set of the l predicted modules.

(i) 3D Recovery score for two tri-clusters

$$3D\ Recovery\ = \frac{1}{k} \cdot \sum_{i=1}^{k} \max_{1 \le j \le l} Jaccard(\ M_i^{True}, M_j^{Pre})$$

where M_i^{True} *and* M_j^{Pre} *are two subtensors/tri-clusters and* $Jaccard$ *is defined as*

$$Jaccard(\ M_i^{True}, M_j^{Pre}) = \frac{\left|\{C_{ijk} \mid C_{ijk} \in M_i^{True}\ and\ C_{ijk} \in M_j^{Pre}\}\right|}{\left|\{C_{ijk} \mid C_{ijk} \in M_i^{True}\ or\ C_{ijk} \in M_j^{Pre}\}\right|}$$

(ii) 2D Recovery score for two bi-clusters

$$2D\ Recovery\ = \frac{1}{k} \cdot \sum_{i=1}^{k} \max_{1 \le j \le l} Jaccard(\ M_i^{True}, M_j^{Pre})$$

where M_i^{True} *and* M_j^{Pre} *are two matrices/bi-clusters and Jaccard index is defined as*

$$Jaccard(\ M_i^{True}, M_j^{Pre}) = \frac{\left|\{C_{ij} \mid C_{ij} \in M_i^{True}\ and\ C_{ij} \in M_j^{Pre}\}\right|}{\left|\{C_{ij} \mid C_{ij} \in M_i^{True}\ or\ C_{ij} \in M_j^{Pre}\}\right|}$$

(iii) 1D Recovery score for two clusters

$$1D\ Recovery\ = \frac{1}{k} \cdot \sum_{i=1}^{k} \max_{1 \le j \le l} Jaccard(\ M_i^{True}, M_j^{Pre})$$

where M_i^{True} and M_j^{Pre} *are two clusters and Jaccard index is defined as*

$$Jaccard(\ M_i^{True}, M_j^{Pre}) = \frac{\left|\{C_i \mid C_i \in M_i^{True}\ and\ C_i \in M_j^{Pre}\}\right|}{\left|\{C_i \mid C_i \in M_i^{True}\ or\ C_i \in M_j^{Pre}\}\right|}$$

20.2 Clustering error (CE) score

An intuitive way to compare the clustering results is to calculate the clustering error (CE). It is the proportion of points which are clustered differently in M^{True} *and* M^{Pre} *after an optimal matching of clusters [9]. Let* $\{(t_i, y_i)\}_{i=1}^{\min\{k,l\}}$ *be a unique relation that maximizes* $d_{max} \triangleq \sum_{i=1}^{\min\{k,l\}} |\ M_{t_i}^{True} \cap M_{y_i}^{Pre}|$*. Then, the CE score is given by*

$$CE\ = 1 - \frac{d_{max}}{|U|}$$

where $|U| = |\{\cup_{i=1}^{k} M_i^{True}\} \cup \{\cup_{j=1}^{l} M_j^{pre}\}|$*. Similarly, we also extend the "CE score" to evaluate the similarity of two tri-clusters, bi-clusters or clusters.*

(i) 3D CE score for two tri-clusters

$$3D\ CE\ = 1 - \frac{d_{max}}{|U|}$$

where $|U| = |\{\cup_{i=1}^{k} M_i^{True}\} \cup \{\cup_{j=1}^{l} M_j^{pre}\}|$ *and* M_i^{True} *and* M_j^{Pre} *are two subtensors/tri-clusters.*

(ii) 2D CE score for two bi-clusters

$$2D\ CE\ = 1 - \frac{d_{max}}{|U|}$$

where $|U| = |\{\cup_{i=1}^{k} M_i^{True}\} \cup \{\cup_{j=1}^{l} M_j^{pre}\}|$ *and* M_i^{True} *and* M_j^{Pre} *are two matrices/bi-clusters.*

(iii) 1D CE score for two clusters

$$1D\ CE\ = 1 - \frac{d_{max}}{|U|}$$

where $|U| = |\{\cup_{i=1}^{k} M_i^{True}\} \cup \{\cup_{j=1}^{l} M_j^{pre}\}|$ *and* M_i^{True} *and* M_j^{Pre} *are two clusters."*

9. The work will be much improved if the biology behind several selected modules is described. How do the discovered modules improve our understanding of pan-cancer gene and miRNA regulation?

Response: This is a valuable suggestion. To this end, we first have shown the results of biological functional analysis for selected modules in **Table S19**. In short, we found that the modules present biological functions and meanings from different perspectives. For example, we found that the expression of miRNAs and genes within these modules are significantly correlated with patient survival in some cancer types (log-rank test BH adjusted $P < 0.05$). We took module 1, 4 and 11 as examples. We have added **a section "3.8 Case studies"** into the main text (**lines 371-423**).

"3.8 Case studies

*Based on the above functional analysis, we found that some identified modules show diverse biological functions and relevance from different views (**Table S19**). We took module 1, 4 and 11 as examples. The module 1 consists of 100 genes, 10 miRNAs and 20 cancers, of which 5 cancer miRNAs and 31 cancer genes (hypergeometric test, P = 2.59e-05). The correlations between miRNAs and genes across the selected cancer types are statistically significant compared to random ones (Permutation test, P < 0.001). For five cancer types (including STAD, BRCA, STES, KICH and SARC), the expression pattern of miRNAs and genes within the module is significantly related with their patient survival respectively (log-rank test BH adjusted P < 0.05, **Supplementary section S18**). Therefore, we may consider module 1 as a potential prognostic biomarker for these five cancer types. Moreover, the module genes are enriched with a large number of cancer-related functional terms including GOBP terms (cell cycle process, mitotic cell cycle, cell cycle, chromosome segregation and cell division) and KEGG pathways (cell cycle, oocyte meiosis, progesterone mediated oocyte maturation, homologous recombination and p53 signaling pathway), suggesting its strong cancer relevance. Recent studies have shown that these cell cycle-related functions are related to multiple cancer processes **[1,2]**. On the other hand, five module miRNAs (hsa-miR-17-5p, hsa-miR-18a-5p, hsa-miR-93-5p, hsa-miR-106b-5p and hsa-miR-106b-3p) belong to miR-17 family, which has been reported to be related to cancer **[3,4]**. Finally, we also found that 6 of 10 miRNAs is related with patient survival in at least one cancer type (log-rank test BH adjusted P < 0.05) **(S3 Appendix Table S18)**. For example, the expression of hsa-miR-130b-5p and hsa-miR-130b-3p are significantly related with ACC patient survival.*

The module 4 contains 5 cancer miRNAs and 25 cancer genes (hypergeometric test, P = 4.66E-03). The correlations between miRNAs and genes across the selected cancer types within this module are statistically significant compared to random ones (Permutation test, P < 0.001). This module is significantly related to the survival time in five cancer types (ACC, LIHC, LUAD, PAAD, KICH). The genes within the module are enriched with some cancer-related functional terms including GOBP terms (cell cycle, cell cycle process, chromosome organization, mitotic cell cycle and DNA metabolic

process) and KEGG pathways (DNA replication, base excision repair, nucleotide excision repair, cell cycle, pyrimidine metabolism). Boyer et al. have reported that DNA replication pathway plays an important role in cancer ***[5]****. More importantly, we found that 57 miRNA-gene interactions between the miRNAs and genes within this module were verified reported before. Collecting the gene-gene network from PPI network, we construct a miRNA-gene-gene regulatory sub-network where there are 7 miRNAs, 75 genes and 309 edges (****Fig 5H and S3 Appendix Table S16****).*

*The last example, module 11 exhibits distinct biological relevance with LGG (Brain Lower Grade Glioma) in terms of miRNAs and genes. Firstly, the miRNAs and genes across the selected cancer types within the module show strong correlations (Permutation test, P < 0.001). Secondly, the genes within this module are enriched with several cancer-related KEGG pathways including cell cycle, small cell lung cancer, DNA replication, mismatch repair. As mentioned earlier, cell cycle and DNA replication pathways have been reported to play an important role in cancer. Thirdly, 36 miRNA-gene interactions between the miRNAs and genes within this module were verified by miRTarBase database. We also construct a miRNA-gene-gene regulatory sub-network, which contains 7 miRNAs, 68 genes and 208 miRNA-gene edges (****S3 Appendix Table S16****). Importantly, two miRNAs (hsa-miR-130b-5p and hsa-miR-130b-3p) within the module belong to mir-130 family, which have been reported as potential biomarkers for brain cancer* ***[6,7,8]****. Especially, the expression pattern of miRNAs and genes within this module is significantly related with LGG patient survival (log-rank test BH adjusted P < 3.18E-06).”*

Overall, these modules identified by TSCCA first show that the miRNA-gene co-expressed patterns of these modules have some different forms of expression. For example, the miRNAs and genes within the module 1, 4 and 10 have strong positive correlations on all the selected cancer types (**Figure S17A**). While the miRNAs and genes in modules 5, 8 and 9 have strong negative correlations on all the selected cancer types (**Figure S17B**). By clustering the output matrix W, we can study the correlation of different cancer types. We found that there are four clusters of 33 cancer types. The first cluster (including STAD, STES, COAD, COADREAD, READ, BLCA and ESCA) has the strongest weight values in W. The second cluster contains TGCT, BRCA, LUSC, LUAD, HNSC, CHOL, UCEC, PAAD, PRAD and CESC, where the LUSC and LUAD show very similar patterns in different modules. The third cluster (including KIPAN, DLBC, UCS, KIRC, KIRP, THCA, OV, PCPG, MESO, LGG and UVM) has the lowest weight values. Several cancer types in the third cluster show module-specific characteristics. For example, we observed that the miRNAs and genes within module 31 are positively correlated in TGCT cancer type, but are negatively correlated in other cancer types, and module 49 are positively correlated in TGCT and UCS cancer types, but are negatively correlated in other cancer types (**Fig S12C**). These results provide a new perspective to improve our understanding of pan-cancer gene and miRNA regulation.

Table S19. Biological functional analysis of selected cancer-miRNA-gene modules. #CM/#CG/#GGE/#MGE, number of cancer miRNAs/cancer genes/gene-gene interaction edges/miRNA-gene interaction edges. Cancer

type: For a given module, these cancer types are in the module and they are clinically related to the module with log-rank test BH adjusted $P < 0.05$ (see **Supplementary Section S20**).

Module	#CM	#CG	#GGE	#MGE	Cancer type	Top enriched GOBP term
1	5	31	505	33	STAD, BRCA, STES, KICH, SARC	cell cycle process, mitotic cell cycle, cell cycle, chromosome segregation, cell division
3	4	19	57	12	BLCA	muscle system process, muscle contraction, muscle structure development, extracellular structure organization, regulation of muscle contraction
4	5	25	253	57	ACC, LIHC, LUAD, PAAD, KICH	cell cycle, cell cycle process, chromosome organization, mitotic cell cycle, DNA metabolic process
10	6	28	536	44	LUAD, PAAD, ACC, KICH	cell cycle, cell cycle process, mitotic cell cycle, organelle fission, mitotic nuclear division
11	5	19	172	36	LGG, ACC, SKCM, KICH	cell cycle, cell cycle process, DNA metabolic process, DNA replication, mitotic cell cycle
19	6	18	263	38	SARC, LIHC	RNA processing, RNA localization, RNA splicing, mRNA processing, cell-cell recognition
30	5	22	207	56	LUAD, PAAD, KICH, SKCM, READ	cell cycle, cell cycle process, mitotic cell cycle, chromosome organization, DNA metabolic process
36	7	19	38	12	KIPAN, BLCA, KIRC	regulation of sequestering of calcium ion, G-protein coupled receptor signaling pathway, regulation of ion homeostasis, cytosolic calcium ion transport

[1] Otto, Tobias, and Piotr Sicinski. "Cell cycle proteins as promising targets in cancer therapy." Nature Reviews Cancer 17.2 (2017): 93.
[2] Sanchez-Vega F, Mina M, Armenia J, Chatila WK, Luna A, La KC, et al. Oncogenic signaling pathways in the cancer genome atlas. Cell. 2018.
[3] Jansson, Martin D., and Anders H. Lund. "MicroRNA and cancer." Molecular oncology 6.6 (2012): 590-610.
[4] Liu, Feifei, et al. "Prognostic role of miR-17-92 family in human cancers: evaluation of multiple prognostic outcomes." Oncotarget 8.40 (2017): 69125.
[5] Boyer, Anne-Sophie, David Walter, and Claus Storgaard Sørensen. "DNA replication and cancer: From dysfunctional replication origin activities to therapeutic opportunities." Seminars in cancer biology. Vol. 37. Academic Press, 2016.
[6] Petrescu, George ED, et al. "MicroRNA based theranostics for brain cancer:

basic principles." Journal of Experimental & Clinical Cancer Research 38.1 (2019): 231.
[7] Gu, Jian-Jun, et al. "Suppression of microRNA-130b inhibits glioma cell proliferation and invasion, and induces apoptosis by PTEN/AKT signaling." International journal of molecular medicine 41.1 (2018): 284-292.
[8]. Huang, Saihua, et al. "Exosomal miR-130b-3p targets SIK1 to inhibit medulloblastoma tumorigenesis." Cell Death & Disease 11.6 (2020): 1-16.

Minor comments

10. Prior work by Tan et al. recently investigated miRNA-gene modules across cancers [2]. Their computational approach is quite naïve, but this work should be mentioned.

Response: In light of your suggestion, we have added the citation and discussion of [2].

[2] Hua Tan, Shan Huang, Zhigang Zhang, Xiaohua Qian, Peiqing Sun, and Xiaobo Zhou. 2019. Pan-cancer analysis on microRNA-associated gene activation. EBioMedicine. 2019 May; 43: 82–97.

11. Though the appendix states where corrections for multiple hypotheses were performed, we think that for clarity, these corrections should be also mentioned in the main text when they are used, and whether reported p-values are after correction.

Response: In light of your suggestion, we have also included it properly.

12. The external datasets used in this study are mentioned at the beginning, but it would be helpful if they are also mentioned when they are being used. For example, in section 3.3 it would be helpful if the databases for cancer genes and miRNA are mentioned.

Response: In light of your suggestion, we have mentioned properly.

13. The text describing Figure 3C is not clear. It resembles the text for 3B, even though these two examples demonstrate very different phenomena.

Response: Thanks for your reminder. In the revised manuscript, we have rephrased it: *"whereas those within module 5 show strong negative correlation on all selected cancers (Fig 3C)."*

14. The objective functions for TSCCA allows for negative W values. It is therefore interesting to visualize W, in addition to the current visualization in Figure 4 of |W|. Are there miRNA-gene modules that are correlated in one cancer type, but are anti-correlated in another? This is briefly discussed in the

discussion, and is seen in the supplementary figures, but will be more easily

Response: In light of your comment, we first show the heatmap of the original W in the **Figure S12A**. There are only three negative elements in W (**Figure S12B**), i.e., (Module 31, TGCT) is -0.145, (Module 49, TGCT) is -0.23 and (Module 49, UCS) is -0.138 and their heatmaps are shown in **Figure S12C**. We observed that the miRNAs and genes within module 31 are positively correlated in TGCT cancer type, but are negatively correlated in other cancer types, and module 49 are positively correlated in TGCT and UCS cancer types, but are negatively correlated in other cancer types (**Figure S12C**).

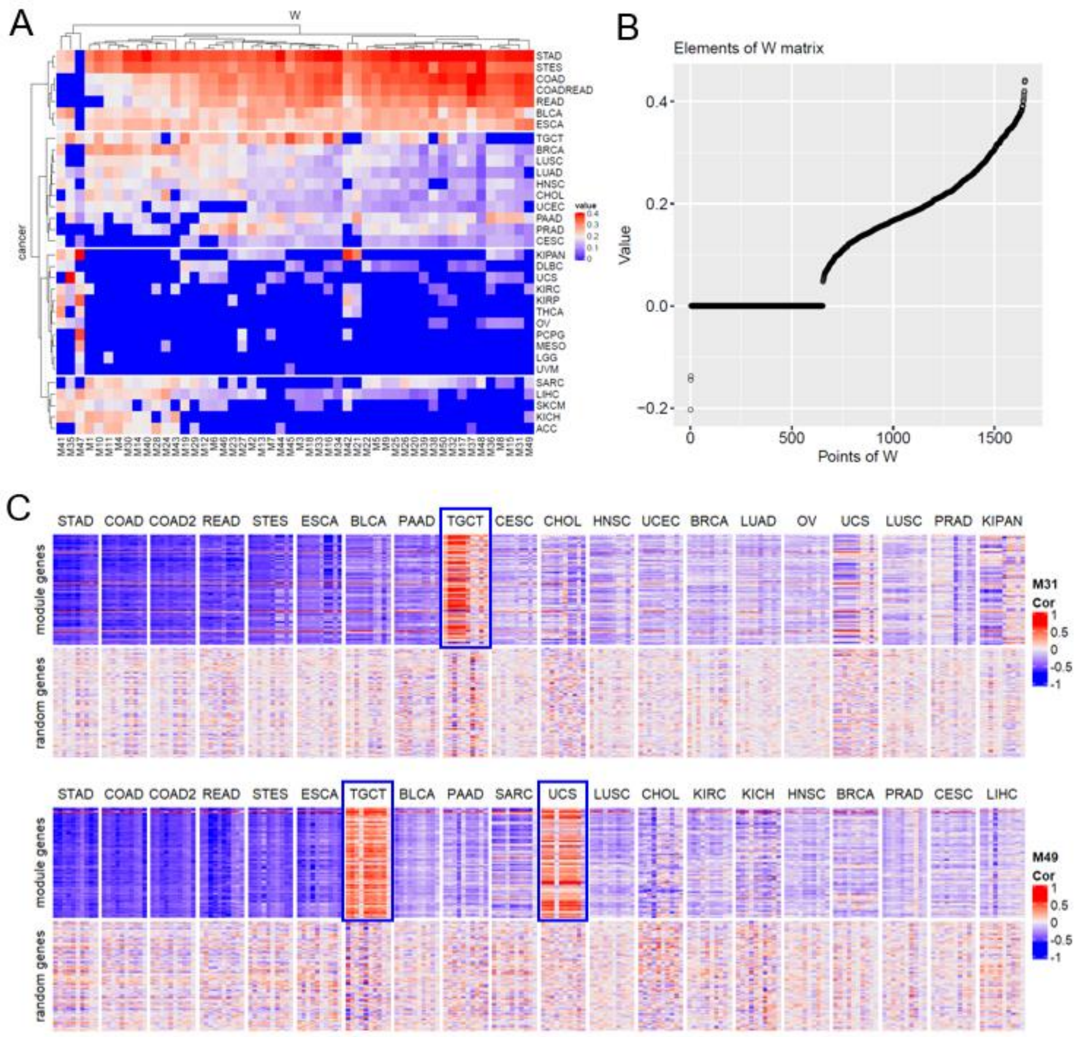

Figure S12. (A) Heatmap showing the output matrix W of Algorithm 2, when it was applied to the TCGA data. Each column corresponds to a module and each row corresponds to a cancer type and W_{ij} reflects the co-expressed intensity between the genes and the miRNAs within the module j on the cancer i. A hierarchical clustering method was used to cluster the rows (cancer types) into four clusters. (B) Scatter plot for elements of the W matrix. There are three negative elements/pairs in W, where (Module 31, TGCT) is -0.145, (Module 49, TGCT) is -0.23 and (Module 49, UCS) is -0.138 and (C) Their heatmaps shown in the blue frame.

To avoid ambiguity, we have updated **Figure 4** which is the heatmap of the original W and added the discussions into main text (**lines 235-241**).

"We first observed that there are only three negative elements in W (Figure S12B), i.e., (Module 31, TGCT) is -0.145, (Module 49, TGCT) is -0.23 and (Module 49, UCS) is -0.138. Interestingly, we also observed that the miRNAs and genes within module 31 are positively correlated in TGCT cancer type, but are negatively correlated in other cancer types, and module 49 are positively correlated in TGCT and UCS cancer types, but are negatively correlated in other cancer types (S2 Appendix Fig S12C)."

15. "For each miRNA-gene module, … have not been verified" – this sentence is not clear. What verification was performed?

Response: This means the miRNA-gene interaction is verified by the miRTarBase database. In light of your comment, we have revised the original sentence as follows:

"For each identified miRNA-gene module, we have confirmed that some miRNA-gene interactions are verified by the miRTarBase database, while there are also many miRNA-gene pairs are not verified by the database."

16. Because each module has more genes than miRNAs, the 1st PC will likely mainly represent the variance in gene expression. It will be interesting to repeat this analysis by taking the 1ST PC using genes only, or the 1st PC using miRNAs only.

Response: In light of your comment, we first obtained the joint gene expression and miRNA expression data: $X = [X_1, X_2, \dots, X_{33}] \in R^{p\times(n1+\cdots+n33)}$ and $Y = [Y_1, Y_2, \dots, Y_{33}] \in R^{q\times(n1+\cdots+n33)}$. We then extracted the 1ST PC (denoted as ***u***) using the gene expression X and the 1ST PC (denoted as ***v***) using the miRNA expression Y. For comparison with TSCCA, the top 100 genes with the largest absolute values of ***u*** and the top 10 miRNAs with the largest absolute values of ***v*** are considered as a module, denoted as pcModule.

We then analyzed the biological functionality for the pcModule from multiple biological perspectives (**Table S26**). We first found that the miRNAs and genes within the module are not strongly correlated on most cancers types (**Figure S15A**). In the pcModule, there are five cancer miRNAs including hsa-miR-324-5p, hsa-miR-484, hsa-miR-186-5p, hsa-miR-590-5p, and hsa-miR-423-3p and the cancer miRNA set enrichment analysis shows the result is not significant.

In addition, there are 15 cancer genes within the module, including IL6ST, GADD45GIP1, NAA10, AFF4, BRMS1, NDUFA13, CHD9, RUVBL2, LNPEP, STAG1, ETV3, ARHGEF12, ATRX, PPTC7, PPP1R14B. Cancer gene set enrichment analysis shows the result is also not significant. More results of pcModule are shown in **Table S26**. We also compared the difference of modularity scores between the pcModule and TSCCA modules, and we found that the modularity value of pcModule is significantly smaller than that of all TSCCA modules (Wilcoxon rank-sum test $P < 0.05$, **Figure S15B**).

Finally, we have added the comparison results into **Supplementary Section S22** "*22.3 Comparison of TSCCA with PCA*" and added the discussions into main text (**lines 484-487**).

Table S26. Results of pcModule. "#cancer miR", "#cancer gene", "#gene edge" and "#miR-gene edge" denote the number of cancer miRNAs, cancer genes, gene edges and miRNA-gene edges. "*" stands for *p*-value < 0.05.

	#cancer miR	#cancer gene	#gene edge	#miR-gene edge	modularity
pcModule	5	15	63*	32	0.15

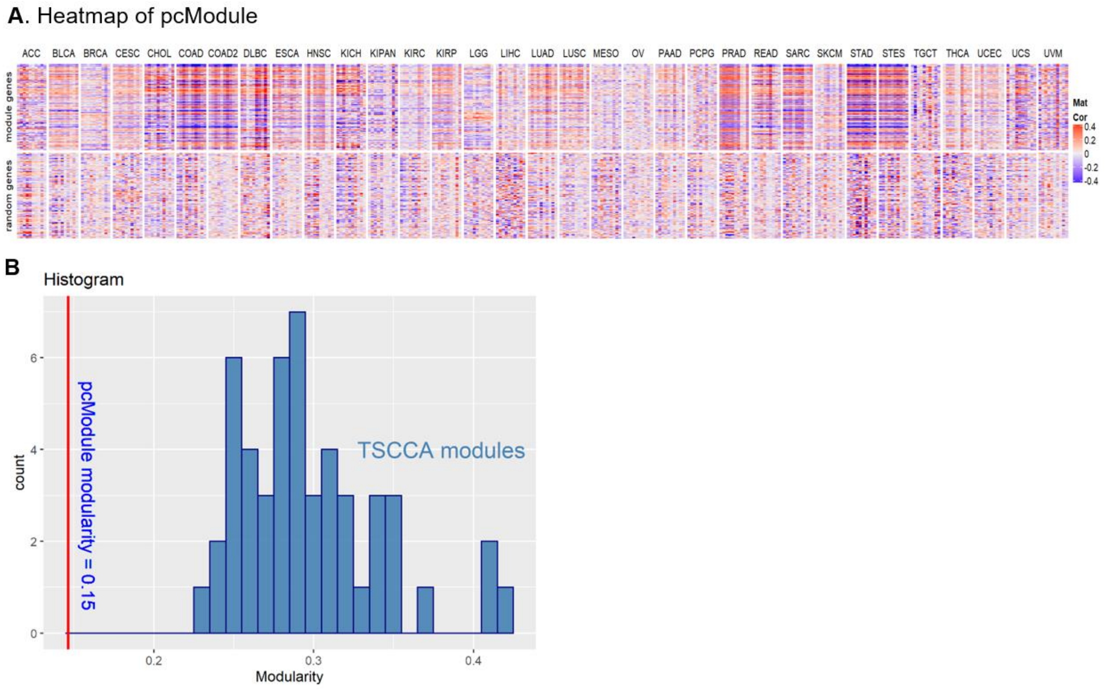

Figure S15. (A) Heatmap of pcModule. The top half of heatmap corresponds to the module (row corresponds to gene, column corresponds to miRNA) and the lower part is a random module for comparison. (B) Comparison of modularity scores of pcModule and TSCCA modules.

17. When describing the survival analysis, modules 11 and 36 are specifically mentioned. We understand that this is because these modules had non-zero entries in W in the cancer types for which they were linked to survival. This should be explicitly stated, as otherwise it is not clear why these modules are mentioned.

Response: Modules 11 and 36 are specifically mentioned because M11-LGG and M36-KIPAN edges have the largest weight value (i.e., smallest *p*-value) in the bipartite graph between the identified modules and the different cancer types (**Figure 7A**). In light of your comment, before modules 11 and 36 are specifically mentioned, we added a sentence into main text (**lines 355-356**):

"We found that M11-LGG and M36-KIPAN edges have the largest weight value (i.e., smallest p-value)."

18. Typos:
a. "For each cancer types, we downloaded..." – should be "type" (p. 3).
b. "We found that 7889…" – "that" should be removed (p. 3).
c. In equation (6), the l0 constraint should be on v, not on u.
d. "While we also found that the modularity…" – remove "While" (p. 7).
e. "negative correlation on all cancers on all cancers". (p. 9).
f. "from a experimentally validated…" – replace "a" with "an" (p. 12).
g. When describing the simulation, the distribution for A_3 is written twice. The second time should be A_4 (p. 15).
h. "it miss some real members" – should be "misses" (p. 15). There are several times "miss" should be replaced with "misses" in this page.

Response: We thank the reviewer for your detailed suggestions. We have carefully polished our manuscript again, and have addressed these typos.

References

[1] - Rui Henriques and Sara C. Madeira. 2018. Triclustering Algorithms for Three-Dimensional Data Analysis: A Comprehensive Survey. ACM Comput. Surv. 51, 5, Article 95 (January 2019), 43 pages. DOI:https://doi.org/10.1145/3195833

[2] - Hua Tan, Shan Huang, Zhigang Zhang, Xiaohua Qian, Peiqing Sun, and Xiaobo Zhou. 2019. Pan-cancer analysis on microRNA-associated gene activation. EBioMedicine. 2019 May; 43: 82–97. doi: 10.1016/j.ebiom.2019.03.082

Response to Reviewer: 2

To identify cancer-specific and shared miRNA-gene co-expressed modules, the authors proposed a tensor sparse canonical correlation analysis (TSCCA) method to analysis of matched miRNA and gene expression data of multiple cancers. The authors first constructed a tensor of gene, miRNA and cancers. Then they decomposed the correlation tensor into a number of latent factors. Finally, based on the non-zero latent factors, they identified cancer-miRNA-gene modules. Application to 33 TCGA cancer types identified novel cancer-related miRNA-gene modules. Here are my comments:

Response: We thank the reviewer for evaluating our work.

1. In simulation, the data was generated from normal distributions with fixed variances. I wonder what would happen if the values were generated with larger variances, instead of 0.04?

Response: In light of your comment, we have reapplied TSCCA to the simulated data with larger variances. We considered the variance (σ^2) with different values including 0.1, 0.3, 0.5 and 1. We then applied TSCCA and SCCA to these generated simulated data with these different variances. Similarly, we found that TSCCA is superior to other methods in terms of CE and recovery scores (**Tables S22 and S23**).

In addition, we have added this analysis and results into **Supplementary Section S21** *"More details about simulation study"* and added the discussion of this into main text (**Lines 445-446**).

Table S22. Comparison (in terms of CE ± std) on the simulated data with different variances. Since SCCA cannot select cancer types, we assumed each module identified by SCCA contained all cancer types for computing 3D-CE.

variance = 0.1	**1D-CE (gene)**	**1D-CE (miR)**	**2D-CE**	**3D-CE**
cancer1	0.769±0.013	0.779±0.041	0.567±0.014	0.323±0.005
cancer2	0.768±0.011	0.778±0.052	0.565±0.015	0.322±0.006
cancer3	0.770±0.014	0.786±0.044	0.569±0.015	0.324±0.006
cancer4	0.363±0.015	0.421±0.039	0.090±0.009	0.058±0.006
Sum	0.370±0.015	0.429±0.039	0.090±0.009	0.058±0.006
Modularity_SA	0.582±0.142	0.667±0.202	0.468±0.137	0.468±0.137
TSCCA	**0.897±0.113**	**0.898±0.115**	**0.805±0.214**	**0.785±0.236**
variance = 0.3	**1D-CE (gene)**	**1D-CE (miR)**	**2D-CE**	**3D-CE**
cancer1	0.682±0.018	0.777±0.041	0.466±0.014	0.273±0.007
cancer2	0.676±0.041	0.768±0.062	0.458±0.042	0.269±0.023
cancer3	0.680±0.017	0.784±0.045	0.465±0.018	0.272±0.009
cancer4	0.363±0.015	0.421±0.039	0.090±0.009	0.058±0.006
Sum	0.370±0.015	0.429±0.039	0.090±0.009	0.058±0.006
Modularity_SA	0.482±0.085	0.625±0.184	0.307±0.085	0.306±0.086
TSCCA	**0.802±0.098**	**0.832±0.109**	**0.637±0.175**	**0.609±0.193**
variance = 0.5	**1D-CE (gene)**	**1D-CE (miR)**	**2D-CE**	**3D-CE**

cancer1	0.595±0.050	0.753±0.083	0.372±0.061	0.222±0.035
cancer2	0.597±0.039	0.761±0.074	0.374±0.046	0.224±0.025
cancer3	0.603±0.040	0.765±0.071	0.380±0.045	0.227±0.025
cancer4	0.363±0.015	0.421±0.039	0.090±0.009	0.058±0.006
Sum	0.370±0.015	0.429±0.039	0.090±0.009	0.058±0.006
Modularity_SA	0.434±0.053	0.575±0.122	0.207±0.051	0.197±0.053
TSCCA	**0.688±0.076**	**0.752±0.094**	**0.467±0.108**	**0.434±0.116**
variance = 1.0	**1D-CE (gene)**	**1D-CE (miR)**	**2D-CE**	**3D-CE**
cancer1	0.463±0.046	0.633±0.104	0.215±0.059	0.134±0.035
cancer2	0.463±0.042	0.637±0.111	0.214±0.057	0.134±0.034
cancer3	0.470±0.037	0.654±0.081	0.222±0.043	0.138±0.026
cancer4	0.363±0.015	0.421±0.039	0.090±0.009	0.058±0.006
Sum	0.370±0.015	0.429±0.039	0.090±0.009	0.058±0.006
Modularity_SA	0.373±0.017	0.455±0.051	0.113±0.018	0.090±0.023
TSCCA	0.424±0.078	0.507±0.114	0.155±0.089	0.106±0.095

Table S23. Comparison of the (in terms of recovery ± std) on the simulated data with different variables. Since SCCA cannot select cancer types, we assumed each module identified by SCCA contained all cancer types for computing 3D-recovery score.

variance = 0.1	**1D-recovery (gene)**	**1D-recovery (miR)**	**2D-recovery**	**3D-recovery**
cancer1	0.728±0.009	0.768±0.022	0.680±0.009	0.346±0.005
cancer2	0.726±0.010	0.773±0.026	0.679±0.009	0.346±0.005
cancer3	0.728±0.009	0.770±0.026	0.681±0.011	0.347±0.005
cancer4	0.229±0.010	0.300±0.038	0.086±0.010	0.055±0.006
Sum	0.232±0.009	0.300±0.032	0.084±0.008	0.055±0.005
Modularity_SA	0.694±0.021	1.000±0.000	0.694±0.021	0.694±0.021
TSCCA	**0.882±0.130**	**0.894±0.117**	**0.860±0.153**	**0.854±0.160**
variance = 0.3	**1D-recovery (gene)**	**1D-recovery (miR)**	**2D-recovery**	**3D-recovery**
cancer1	0.578±0.019	0.766±0.021	0.530±0.018	0.285±0.008
cancer2	0.572±0.033	0.766±0.047	0.522±0.047	0.282±0.023
cancer3	0.572±0.019	0.770±0.026	0.525±0.021	0.284±0.010
cancer4	0.229±0.010	0.300±0.038	0.086±0.010	0.055±0.006
Sum	0.232±0.009	0.300±0.032	0.084±0.008	0.055±0.005
Modularity_SA	0.481±0.022	0.948±0.062	0.463±0.037	0.463±0.037
TSCCA	**0.748±0.110**	**0.823±0.113**	**0.711±0.133**	**0.702±0.139**
variance = 0.5	**1D-recovery (gene)**	**1D-recovery (miR)**	**2D-recovery**	**3D-recovery**
cancer1	0.467±0.033	0.739±0.078	0.408±0.059	0.230±0.030
cancer2	0.468±0.025	0.752±0.063	0.412±0.046	0.233±0.022
cancer3	0.471±0.032	0.751±0.051	0.416±0.044	0.235±0.022
cancer4	0.229±0.010	0.300±0.038	0.086±0.010	0.055±0.006
Sum	0.232±0.009	0.300±0.032	0.084±0.008	0.055±0.005
Modularity_SA	0.358±0.030	0.680±0.111	0.274±0.051	0.274±0.051
TSCCA	**0.592±0.081**	**0.743±0.097**	**0.536±0.102**	**0.524±0.108**
variance = 1.0	**1D-recovery (gene)**	**1D-recovery (miR)**	**2D-recovery**	**3D-recovery**
cancer1	0.322±0.039	0.560±0.119	0.220±0.062	0.134±0.035
cancer2	0.321±0.037	0.563±0.119	0.219±0.059	0.133±0.033
cancer3	0.329±0.028	0.579±0.093	0.228±0.045	0.139±0.025
cancer4	0.229±0.010	0.300±0.038	0.086±0.010	0.055±0.006
Sum	0.232±0.009	0.300±0.032	0.084±0.008	0.055±0.005

Modularity_SA	0.247±0.017	0.363±0.064	0.114±0.023	0.105±0.030
TSCCA	0.292±0.076	0.407±0.144	0.163±0.104	0.124±0.114

2. It is claimed that TSCCA method can identify both cancer-specific and shared miRNA-gene co-expressed modules. I wonder whether TSCCA identified any shared miRNA-gene co-expressed modules across 33 TCGA cancer types. If so, what properties do the shared modules have?

Response: We first explain some properties cancer-specific and shared modules. The so-called miRNA-gene co-expressed shared module means that the genes and miRNAs in this module are strongly correlated in expression on most cancers, and it is a relative definition in our study. For example, the miRNAs and genes in modules 1, 4, and 10 have a strong positive correlation on all the cancer types (**Figure S17A**). The miRNAs and genes in modules 5, 8, and 9 have a strong negative correlation on all the selected cancer types (**Figure S17B**). We call these modules shared modules.

Cancer is a complex and heterogeneous disease. Each cancer type has specificity and commonality with other cancers. The purpose of the paper is to use the TSCCA model to discover these patterns. If there is a shared module in all 33 cancers, we believe our model is capable to detect this module. Finally, we have added a **Supplementary Section S24** to introduce what is specific and shared modules.

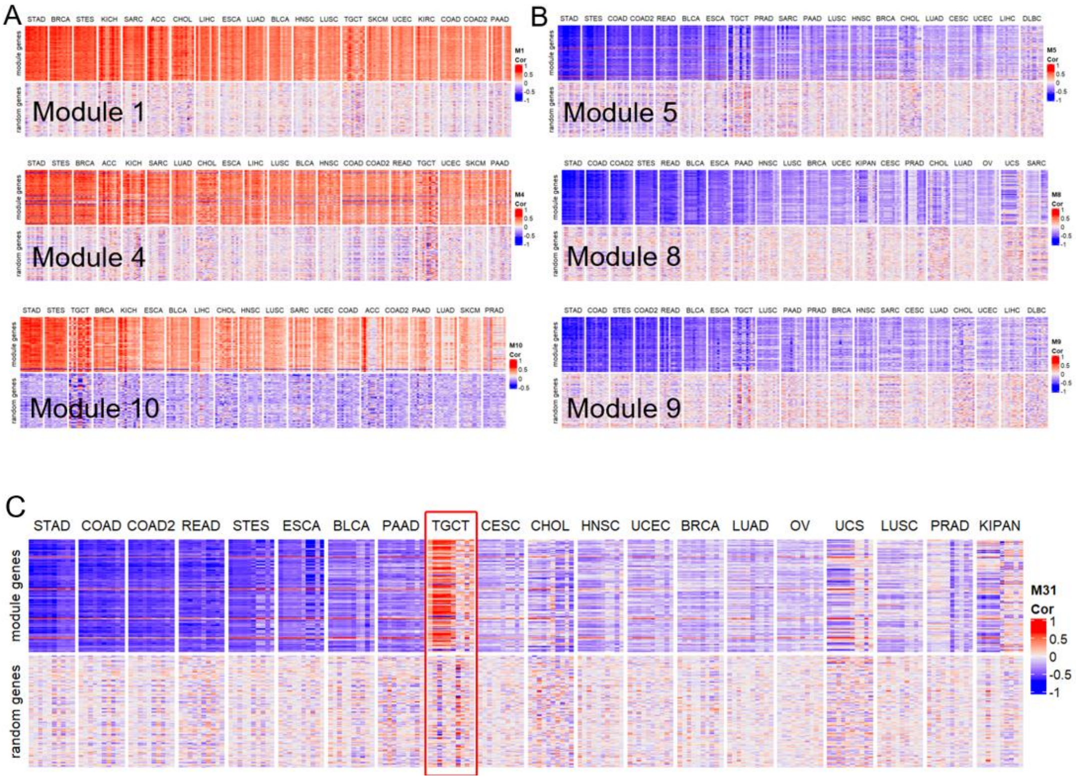

Figure S17. Heatmap of some modules identified by TSCCA in the TCGA dataset, where (A) corresponds to modules 1, 4 and 10 (B) corresponds to modules 5, 8 and 9. (C) Heatmap of cancer-miRNA-gene module 31 identified by TSCCA in the TCGA dataset. Module 31 is a TGCT-cancer-specific miRNA-gene co-expressed module.

3. The authors evaluated the performance of TSCCA with other methods on the TCGA data mainly using the modularity score. They may want to evaluate the modules identified by the comparison methods using other measurements, for example, they may check the enrichment of cancer related genes/miRNAs among these modules.

Response: In light of your suggestion, we have compared TSCCA with other methods in terms of multiple biological indicators (see **Table S25**). In short, we found that TSCCA has some advantages in multiple biological indicators, such as the average number of cancer miRNAs, cancer genes, gene edges and miRNA-gene edges.

Table S25. Performance comparison of TSCCA and the triclustering methods. "#cancer miR", "#cancer gene", "#gene edge" and "#miR-gene edge" denote the average of the number of cancer miRNAs, cancer genes, gene interactions/edges and miRNA-gene interactions/edges on all the identified modules.

Method	#cancer_miR	#cancer_gene	#gene edge	#miR-gene edge	Modularity
**TSCCA**	**5.98**	**19.56**	**106.10**	**22.70**	**0.30**
**SCP**	5.80	12.54	67.98	14.92	0.29
**Modularity_SA**	8.08	21.14	47.52	11.62	0.29
**Var_SA**	3.40	13.58	32.04	4.94	0.04
**MSR_SA**	3.48	12.30	32.22	7.14	0.06

Finally, we have added these new results into **Supplementary Section S22** *"More details about comparison of TSCCA with other methods"*, and added discussion of this into the main text (**lines 466-483**).

"Secondly, we also compared TSCCA with multiple tri-clustering methods including Modularity_SA and Sparse Canonical Polyadic decomposition (SCP) which uses ℓ_1-regularization to force sparse [49], and two merit-function based methods including "Variance" (see Eq. 1 in [50]) and "Mean squared residue (MSR)" (see Eq. 3 in [50]). The two merit-functions are optimized by using annealing algorithm. Var_SA is a variance-based simulated annealing (Var_SA) method, which uses a simulated annealing algorithm to minimize the variance merit-function for extracting a cancer-miRNA-gene module. Similarly, MSR_SA is an MSR-based simulated annealing (MSR_SA) method, which uses a simulated annealing algorithm to minimize the MSR merit-function for extracting a cancer-miRNA-gene module. The comparison results are given in S3 Appendix Table S25 and show that TSCCA is superior to the other tri-clustering methods in terms of multiple biological indicators and modularity score. Due to the definition of MSR, the MSR_SA method is very consuming time. We found that MSR_SA took an hour to identify a module, while Var_SA only takes 5 seconds on a personal computer. Compared with the TSCCA and Modularity_SA, the sub-tensors/modules identified by Var_SA or MSR_SA tend to be zero patterns (S2 Appendix Fig S14). In short, the TSCCA method is superior to other tri-clustering methods in terms of multiple indicators (S3 Appendix Table S25)."
