# Supplementary material for: TSCCA: A tensor sparse CCA method for detecting microRNA-gene patterns from multiple cancers
Source: PLoS Comput Biol. 2021 Jun 1;17(6):e1009044. doi: 10.1371/journal.pcbi.1009044 (PMC8195367; doi:10.1371/journal.pcbi.1009044)
Supplement: Attachment — Submitted filename: Response_Letter_0314.pdf [file pcbi.1009044.s040.pdf]

# Response to Reviewer #1

**Major comment**

The one-sample Wilcoxon signed-rank test is used several times in the manuscript, e.g. in the analysis in section 3.2. We do not think this test is appropriate here. The null hypothesis for the Wilcoxon test is that the modularity score for a module is higher than the median for the randomly generated modules. This p-value can be very low even if the module's modularity score is not very extreme in comparison to the modularity scores of the random modules. For example, consider a module with modularity score 2000, and 1000 random modules with scores 1401, 1402, …, 2400. The p-value for Wilcoxon will be very small (<2e-16 according to our test in R), because we can confidently say that the modularity of the original module (2000) is higher than the median modularity of the random modules. However, 40% of the random modules have a higher score. A better way to calculate an empirical p-value is to count the number of randomly permuted modules with modularity score equal or greater than 2000. In the same section (3.2), it is not clear how a single p-value is calculated. Section S8 in the appendix only describes how to calculate a p-value for a single module, and it is not clear how these p-values are integrated into a single one (which is stated in the paper as "P < 0.001"). This comment doesn't apply only to section 3.2, but to other cases in which the one-sample Wilcoxon signed-rank test is used (e.g. the number of modules with at least 2 miRNAs from the same family).

**Response:** Thanks for this kind suggestion. To this end, we have made the following changes:

(1) We modified the calculation of p-value for permutation test in Section 3.2, as follows:

*"Step 3. Combine m0 and m1, …, m1000, and compute the p-value of the module using the following formula:*

$$p - value \ = \frac{\sum_{i=1}^{1000} 1(m_i \geq m_0)+1}{1000+1}.$$

*where adding 1 to both numerator and denominator to avoid p-value of zero."*

(2) Regarding the question "Section S8 in the appendix only describes how to calculate a p-value for a single module, and it is not clear how these p-values are integrated into a single one", we are sorry for the unclear description. For multiple p-values, to avoid false positives caused by multiple tests, these identified modules with p-values smaller than 0.05/k were considered as significant ones, where k is the number of identified modules. We revised the sentence into the main text (lines 191-192):

*"The identified modules with p-values smaller than 0.05/k were considered as significant ones, where k is the number of the identified modules. We found that the modularity scores of these modules are significantly larger than those of the random ones (permutation test P < 0.05/50) (Fig 2C and S1 Appendix Section S8)."*

Additionally, we also revised the sentences in the legend of Figure 2 (C):

*"(C) Distribution of modularity scores. The modularity scores of identified modules are significantly greater than those of random ones (permutation test P < 0.05/50 for each identified module)."*

(3) We modified the method of calculating the p-value for permutation test in Appendix Section S17. In the revised manuscript, we rephrased the results with the following sentences:

*"We found that 92% (46 out of 50) modules have at least two miRNAs in the same family (Permutation test P < 0.01, S3 Appendix Table S15 and S1 Appendix Section **S17**)." and "We found that 70% modules have at least three miRNAs participating in a three-layer network (Permutation test P < 0.01, S3 Appendix Table S16 and S1 Appendix Section S17)."*

(4) Lastly, we also made the similar changes regarding the p-value calculation for permutation test in Supplementary Sections 8, 9 and 17.

Other important comments

- To perform permutation testing while conditioning on the degrees in a network, the authors sample genes such that the sum of their degrees is close to the sum of degrees in the original gene set. This conditions on the sum of degrees, but not on the full degree distribution. The common way to perform permutations while conditioning on the degree is to permute gene names only between genes with the same degree. For example, a gene with degree 5 can only be replaced in the permutation with another gene of degree 5. (If sample is insufficient, this can be done by forming bins of genes by the degree, and permuting between genes from the same bin).

**Response:** In light of your suggestion, we adopted this common way to perform permutations. We revised Sections S15 and S16 in the Supplementary Materials and added the results into Supplementary Section S23 "Results of gene-gene and miRNA-gene interaction set enrichment". Accordingly, we revised the discussion in main text (line 291-294 and 331-335):

*"In addition, to avoid the influence of degree in the gene interaction network, we developed a statistical permutation test method to perform the gene-gene interaction set enrichment, and found that 88% (44 out of 50) modules contain significantly more gene interactions than expected by chance (permutation test P < 0.05, S1 Appendix Section S23)."*

*"In addition, to avoid the influence of degree for miRNAs in the miRNA-gene network, we developed a statistical permutation test method to perform the miRNA-gene interaction set enrichment (S1 Appendix Section S23). There are 28% (14 out of 50) modules, which contain significantly more miRNA-gene interactions than expected by chance (permutation test P < 0.05, S1 Appendix Section S23)."*

*Finally, we have revised Sections S15 and S16 into the Supplementary Materials (see*

*the underlined text here).*

***"15.1 Permutation test for gene-gene interaction set enrichment analysis***
*For a given module $i$, suppose it contains $n_0$ genes and $m_0$ validated gene interactions/edges and the sum of degrees of genes within the module in the original gene-gene interaction network is $d_0$.*

*Step 1. We first randomly generate 1000 modules by permuting gene names only between genes with the same degree, so as to eliminate the influence of the vertex degree on the result.*

*Step 2. We then compute the number of validated gene interactions of these random modules, denoted as $m_1, m_2, \dots, m_{1000}$.*

*Step 3. For the given module $i$ and its p-value was computed by using the formula:*

$$p - value = \frac{\sum_{i=1}^{1000} 1(m_i \geq m_0)+1}{1000+1}.$$

*where adding 1 to both numerator and denominator to avoid p-value of zero.*

***16.1 Permutation test for miRNA-gene interaction set enrichment analysis***
*For a given module $i$, suppose it contains $n_0$ genes and $m_0$ validated miRNA-gene interactions/edges and the sum of degrees of miRNAs within the module in the original miRNA-gene interaction network is $d_0$.*

*Step 1. We first randomly generate 1000 miRNA-gene modules by permuting gene/miRNA names only between genes/miRNAs with the same degree, so as to eliminate the influence of the degree on the result.*

*Step 2. We then compute the number of validated miRNA-gene interactions of these random modules, denoted as $m_1, m_2, \dots, m_{1000}$.*

*Step 3. For the given module $i$ and its p-value is computed by using the formula:*

$$p - value = \frac{\sum_{i=1}^{1000} 1(m_i \geq m_0)+1}{1000+1}.$$

*where adding 1 to both numerator and denominator to avoid p-value of zero."*

- Two of our major comments were that further comparison to triclustering methods is required, and that the advantage of using multiple cancers is not sufficiently shown. The authors addressed these points, but some of the results from these new analyses are only mentioned briefly in the discussion, while they should be stated more clearly:

o The W matrix visualization shows that a few cancers dominate all the created modules. In the discussion the authors mention this, and show another analysis in which these dominant cancers are removed (in appendix figure S16). This point, that a small number of cancers may dominate the results, is a major caveat of the analysis,

and as such it should be mentioned when first presenting TSCCA's results, and not briefly referred to in the discussion.

**Response:** Thanks for your kind suggestion. We have added the following discussions in the main text (lines 255-264).

*"We note that TSCCA is an explorative tool, which identifies the "strongest" modular patterns in the current multiple cancer data. This means that in a subset of cancer data, it could identify other significant modules. For example, most of the 50 modules identified by TSCCA on the TCGA dataset are enriched in 60% of cancers, while other cancers are rare. To this end, we may extract a subset of cancers from the cluster 3 in Fig 4 and then re-use TSCCA to extract some modules on a subset of the previous data (across 18 cancers). We found some new modules with significant modularity scores, and more details were given in S2 Appendix Fig S16. This procedure will overcome the limit that a small number of cancers may dominate the results for TSCCA."*

o Modularity_SA has very good results in terms of the number of cancer genes and miRs, while TSCCA is better in terms of the number of gene-gene and miR-gene edges. The good performance of Modularity_SA, and its advantages in comparison to TSCCA, should be mentioned in the main text.

**Response:** In light of your suggestion, we added the following discussion in the main text (lines 493-500):

*"We found that Modularity_SA has good performance in terms of the number of cancer genes and miRNAs, while TSCCA is better in terms of the modularity score and the number of gene-gene and miRNA-gene edges (S3 Appendix Table S25). In addition, we also compared the performance of TSCCA and Modularity_SA under the same input data. Compared with Modularity_SA, TSCCA obtained higher modularity scores and consumed less time (S2 Appendix Fig S16). Therefore, from the perspective of maximizing the modularity score, TSCCA is still better than the Modularity_SA."*

Minor comment

In tables reporting the results of TSCCA, the font in the rows representing TSCCA's results is bold. It is common practice to mark in bold the best result in each column, and we suggest the authors do the same here, or remove the bold font from TSCCA's row. Otherwise, it looks as if TSCCA always has the best performance.

**Response:** Thanks for the suggestion. We removed the bold font in the Table S25.

Typos:

- "within this module were verified reported before" (p. 12) – remove "verified" or "reported".
- "We assessed the similarity of between the true modules" (p. 13) – remove "of".

- “miRNA-gene correlation patterns are heterogeneity” (p. 15) – should be “heterogeneous”.
- “explorative tool, which identity” (p. 15) – should be “identify”.

**Response:** We corrected these typos and further polished the manuscript again.

# Response to Reviewer #2

The authors conducted simulation and real data studies to address the questions. There is a question about Table S25. The authors compared TSCCA with other methods using multiple biological indicators and modularity score and concluded TSCCA is superior to the other tri-clustering methods. However, Modularity_SA identified more cancer_miR and cancer_gene than the TSCCA. The authors may want to discuss this before directly concluding that TSCCA is superior to the other tri-clustering methods.

**Response:** We appreciate very much for your suggestion. Modularity_SA has good performance in terms of the number of cancer genes and miRNAs, while TSCCA is better in terms of the number of gene-gene and miRNA-gene edges. In addition, we also compared the performance of TSCCA and Modularity_SA under the same input data to identify a module using different starting points. We found that the modularity scores of these identified modules by TSCCA are significantly greater than that of these identified modules by Modularity_SA (Fig S16). Therefore, from the perspective of maximizing the modularity score, TSCCA is still better than the Modularity_SA. We added the following discussion about their differences in detail (lines 493-500):

*"We found that Modularity_SA has good performance in terms of the number of cancer genes and miRNAs, while TSCCA is better in terms of the modularity score and the number of gene-gene and miRNA-gene edges (S3 Appendix Table S25). In addition, we also compared the performance of TSCCA and Modularity_SA under the same input data. Compared with Modularity_SA, TSCCA obtained higher modularity scores and consumed less time (S2 Appendix Fig S16). Therefore, from the perspective of maximizing the modularity score, TSCCA is still better than the Modularity_SA."*

Additionally, we also revised the following section in the Supplementary Materials.

***"22.3 Comparison between TSCCA and Modularity_SA on the TCGA data in terms of modularity score***

*Based on the TCGA data, we generated a joint gene expression and miRNA expression data:* $X = [X_1, X_2, \dots, X_{33}] \in R^{p\times(n1+\cdots+n33)}$ *and* $Y = [Y_1, Y_2, \dots, Y_{33}] \in R^{q\times(n1+\cdots+n33)}$ *(referred to as "JointData" data). Since we don't know the true modules on TCGA data, we cannot rely on the CE or Recovery scores to judge the superiority of different methods. We used the modularity score (Eq. 9) to judge the quality of the tested methods. We applied TSCCA and Modularity_SA to identify the first module on the TCGA data with 50 different initializations. To make a fair comparison of TSCCA and SCCA, we also applied SCCA to each single cancer data (*$X^i$ *and* $Y^i$*) and the "JointData" data (*$X$ *and* $Y$*). The parameters of SCCA and Modularity_SA are consistent with the parameters of TSCCA with* $k_u = 200$*,* $k_v = 10$ *and* $k_w = 20$*. Compared with SCCA and Modularity_SA, TSCCA obtained higher modularity scores*

*(Figure S16A). We also compared the running time of different methods on a personal laptop. TSCCA took about 12 seconds to identify a module on average, while SCCA took the least time (Figure S16B)."*

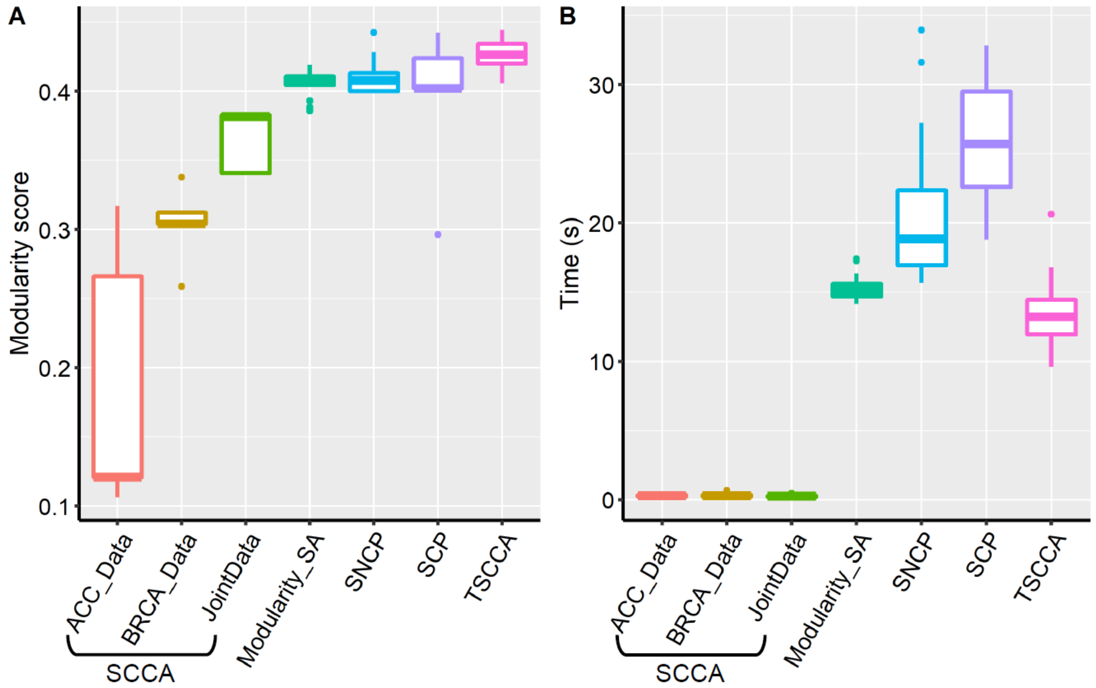


***Figure S16.*** *Comparison of different methods on the TCGA data in terms of Modularity score (A) and time (B). We also compared the running time of different methods on a personal laptop. Box-plots show results in terms of modularity scores and running time of algorithm based on 50 different initializations of each method.*

Lastly, we uploaded the R scripts and the corresponding data for the figures and results presented in our manuscript at the GitHub repository https://github.com/wenwenmin/TSCCA and provided the guide for potential users.
